# Arylisoquinoline-derived organoboron dyes with a triaryl skeleton show dual fluorescence

**DOI:** 10.3762/bjoc.15.254

**Published:** 2019-11-04

**Authors:** Vânia F Pais, Tristan Neumann, Ignacio Vayá, M Consuelo Jiménez, Abel Ros, Uwe Pischel

**Affiliations:** 1 CIQSO – Centre for Research in Sustainable Chemistry and Department of Chemistry, University of Huelva, Campus de El Carmen s/n, 21071 Huelva, Spain; 2 Department of Chemistry/Institute of Chemical Technology UPV-CSIC, Universitat Politècnica de València, Camino de Vera s/n, 46022 Valencia, Spain; 3 Institute for Chemical Research (CSIC-US) and Innovation Center in Advanced Chemistry (ORFEO−CINQA), C/ Américo Vespucio 49, 41092 Seville, Spain; 4 Department of Organic Chemistry, University of Seville, C/ Prof. García González 1, 41012 Seville, Spain

**Keywords:** anions, dyes, fluorescence, laser-flash photolysis, organoboron

## Abstract

Four new dyes that derive from borylated arylisoquinolines were prepared, containing a third aryl residue (naphthyl, 4-methoxynaphthyl, pyrenyl or anthryl) that is linked via an additional stereogenic axis. The triaryl cores were synthesized by Suzuki couplings and then transformed into boronic acid esters by employing an Ir(I)-catalyzed reaction. The chromophores show dual emission behavior, where the long-wavelength emission band can reach maxima close to 600 nm in polar solvents. The fluorescence quantum yields of the dyes are generally in the range of 0.2–0.4, reaching in some cases values as high as 0.5–0.6. Laser-flash photolysis provided evidence for the existence of excited triplet states. The dyes form fluoroboronate complexes with fluoride anions, leading to the observation of the quenching of the long-wavelength emission band and ratiometric response by the build-up of a hypsochromically shifted emission signal.

## Introduction

Boron-containing tri- and tetra-coordinated chromophores have attracted considerable interest due to their often peculiar and highly advantageous photophysical properties that include spectrally tunable and highly intense fluorescence [1–2]. On the one hand, those compounds that contain the boron atom in a valence-saturated situation corresponding to sp^3^ hybridization (such as Bodipy dyes [3–4], N,C-chelate organoboron dyes [5–9], BASHY dyes [10–11] or Boranils [12–13]) often feature quite rigid structures which contribute to high fluorescence quantum yields. These dyes have been applied for example in optoelectronics [14–16], sensing [17–20], and bioimaging [6,20–26]. On the other hand, boron with sp^2^ hybridization, such as in triarylboranes, offers the possibility to modulate fluorescence properties by the addition of Lewis bases (e.g., fluoride ions [27–31]) or by exploring the electron-accepting properties of the boron, including charge-transfer and photoinduced electron-transfer phenomena or two-photon absorption [32–36].

As part of our research program we have developed arylisoquinolines that integrate a boronic acid ester [37–39] or a BMes_2_ unit [6,40]. The presence of the boron-substituent confers interesting photophysical properties to these dyes such as intramolecular charge-transfer processes and tunable red-shifted emission bands. Generally, the so far investigated borylated arylisoquinoline dyes show principally fluorescence quenching (on-off switching) on the formation of the corresponding fluoroboronate complexes [37].

**Figure 1:**
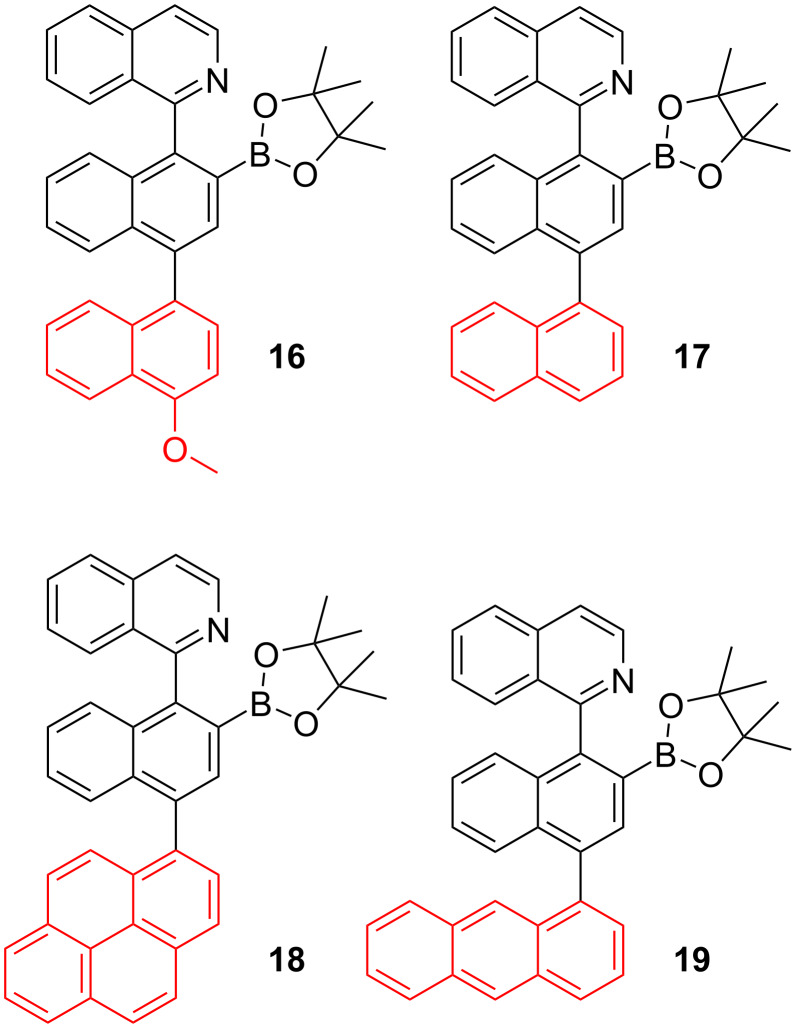
Structures of the dyes **16**–**19**.

Herein, we extended our previously reported arylisoquinoline-derived organoboron dye platform with an additional axially linked aryl residue (see structures **16**–**19** in Figure 1) in the expectation to modulate the fluorescence properties and fluoride response of these dyes. The additional aryl residues allow the verification of the effect of aromatic conjugation (naphthyl, anthryl, pyrenyl) and electron-donor strength (naphthyl versus 4-methoxynaphthyl) on the photophysical properties. Beside the observation of interesting dual emission properties for these dyes, some showed a pronounced ratiometric fluorescence response on fluoride ion addition.

## Results and Discussion

### Synthesis of the borylated dyes **16–19**

**Scheme 1:**
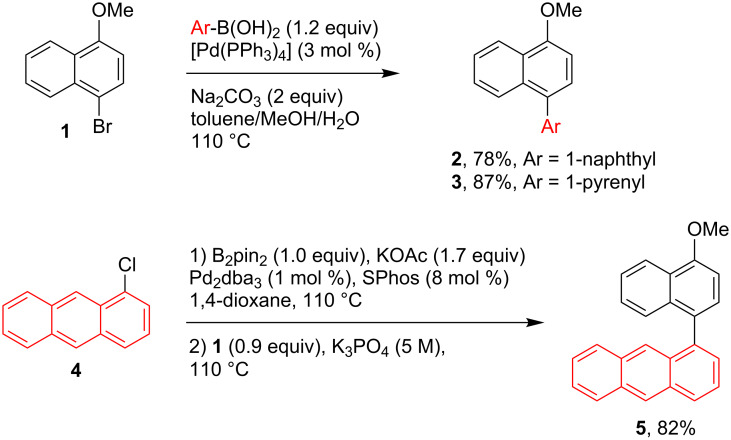
Synthesis of the precursors **2**, **3**, and **5**.

**Scheme 2:**
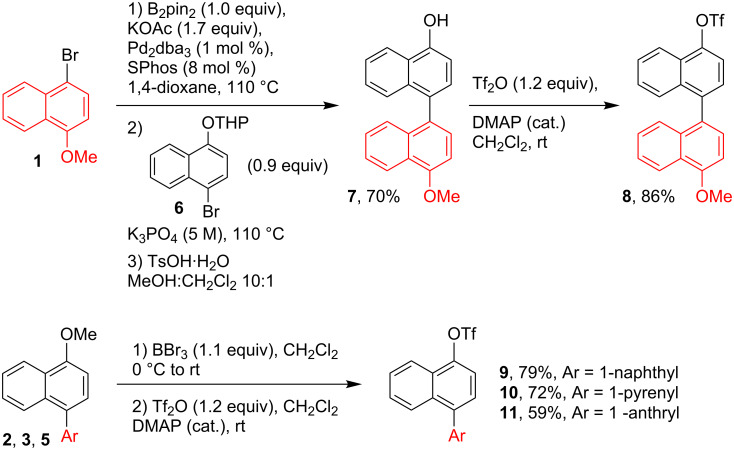
Synthesis of the precursor triflates **8**–**11**.

For the synthesis of the triaryl systems **12**–**15**, precursors of the organoboron dyes **16**–**19**, the construction of two stereogenic axes was required. Therefore, a synthetic route based on consecutive cross-coupling reactions was planned. Starting from 1-bromo-4-methoxynaphthalene (**1**), the Pd-catalyzed Suzuki coupling reaction with commercial boronic acids afforded the naphthyl and pyrenyl derived methyl ethers **2** and **3** in 78% and 87% yield, respectively (Scheme 1). For the synthesis of the anthryl derivative **5** a Pd-catalyzed one-pot reaction consisting of a borylation and Suzuki coupling was applied. Thus, starting from 1-chloroanthracene (**4**) and using SPhos/Pd_2_dba_3_ (8:1) as the catalyst, a full conversion to the Miyaura-type borylated intermediate was achieved (TLC analysis) after 5 hours at 110 ºC. The addition of 1-bromo-4-methoxynaphthalene (**1**, 0.9 equiv) and K_3_PO_4_, and stirring overnight at 110 ºC, afforded the biaryl methyl ether **5** in an 82% yield (Scheme 1). Similarly, Buchwald´s methodology [41] was applied in the synthesis of **7,** which was obtained in 70% yield after tetrahydropyran (THP) group deprotection in MeOH/CH_2_Cl_2_ using TsOH·H_2_O as the catalyst (Scheme 2). In a conventional triflation (Tf_2_O, DMAP cat.), **7** was converted into **8** with a yield of 86%. For the synthesis the triflates **9**–**11** a one-pot demethylation–triflation sequence was followed (Scheme 2). The treatment of biaryl methyl ethers **2**, **3** or **5** with BBr_3_ (1.1 equiv) in anhydrous CH_2_Cl_2_ (0 ºC→rt) allowed the transformation into the alcohol intermediates, which were treated with triflic anhydride (Tf_2_O) in dry dichloromethane to afford **9**–**11** in 59–79% yield.

**Scheme 3:**
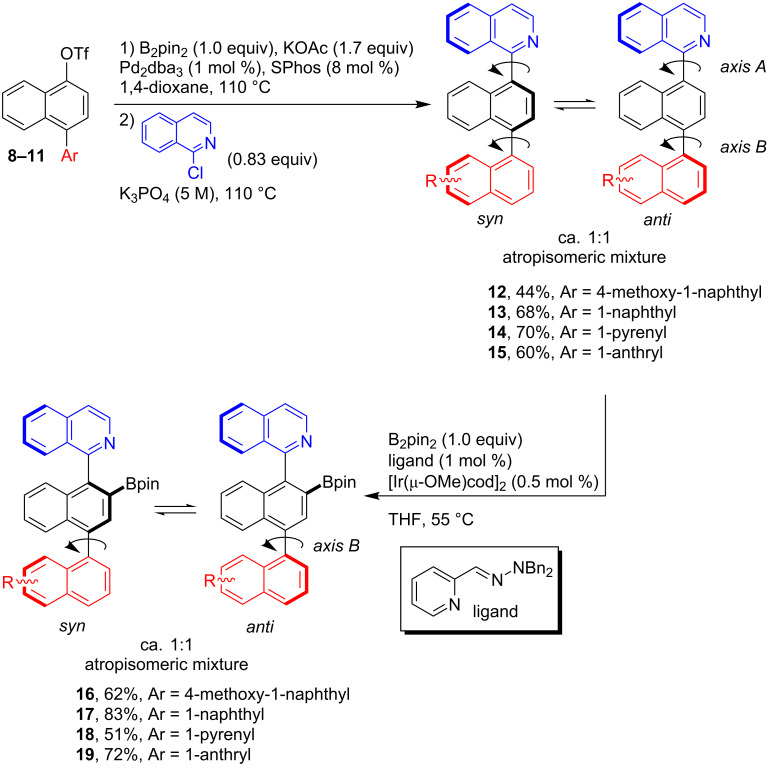
Synthesis of **12**–**15** and the organoboron dyes **16**–**19**.

With the triflates **8**–**11** at hand, these were transformed into the triaryl systems **12**–**15** following a similar Pd-catalyzed one-pot borylation-Suzuki coupling strategy as mentioned above, using 1-chloroisoquinoline as the coupling partner (Scheme 3). The desired compounds **12**–**15** were obtained in 44–70% yield. The ^1^H NMR spectra, recorded at 25 °C, showed the coexistence of the *syn* and *anti* atropisomers because of the slow rotation around the chiral axis at this temperature. Free rotation around the C–C bond was observed at 80 °C and hence, variable-temperature ^1^H NMR studies showed coalescence of the signals to give an average spectrum (see Supporting Information File 1).

The synthesis of the borylated dyes **16**–**19** was carried out following a methodology that was previously reported by some of us [42] and that is based on the Ir-catalyzed nitrogen-directed *ortho*-borylation of arylisoquinolines [37–38]. Despite of the presence of many aromatic C–H bonds which could be borylated, the choice of a suitable pyridine-hydrazone ligand [42] allowed to perform the borylation reactions at 55 °C, showing complete regioselectivity in the C–H borylation. This procedure afforded the dyes **16**–**19** in good to very good yields of 51–83% (Scheme 3). The introduction of the Bpin moiety hinders the free rotation around axis A (Scheme 3) of the compounds **16**–**19**; therefore, complex mixtures of the *syn*/*anti* atropoisomers (0.45:0.55; *syn*:*anti*) were observed in NMR spectroscopy. To facilitate the C–C bond rotation around axis B (Scheme 3) and simplify the NMR spectra, the measurements were undertaken at 80 °C in C_6_D_6_ using a screw-cap NMR tube. Although significant changes were registered, a complete coalescence of the signals was not observed. The chiral HPLC analysis (see HPLC traces in Supporting Information File 1) demonstrated the high purity of compounds **16**–**19**. The sharp peaks and separation times higher than 2 minutes are in accordance with a high rotation barrier. All compounds were identified by their ^1^H and ^13^C NMR spectra. The sp^2^ character of the boron was confirmed by ^11^B NMR spectroscopy, revealing a typical resonance signal at 31–32 ppm [43]. Hence, the isoquinoline nitrogen does not engage in the formation of an intramolecular Lewis pair, akin to related borylated arylisoquinolines [37–38].

### UV–vis absorption and fluorescence properties

**Figure 2:**
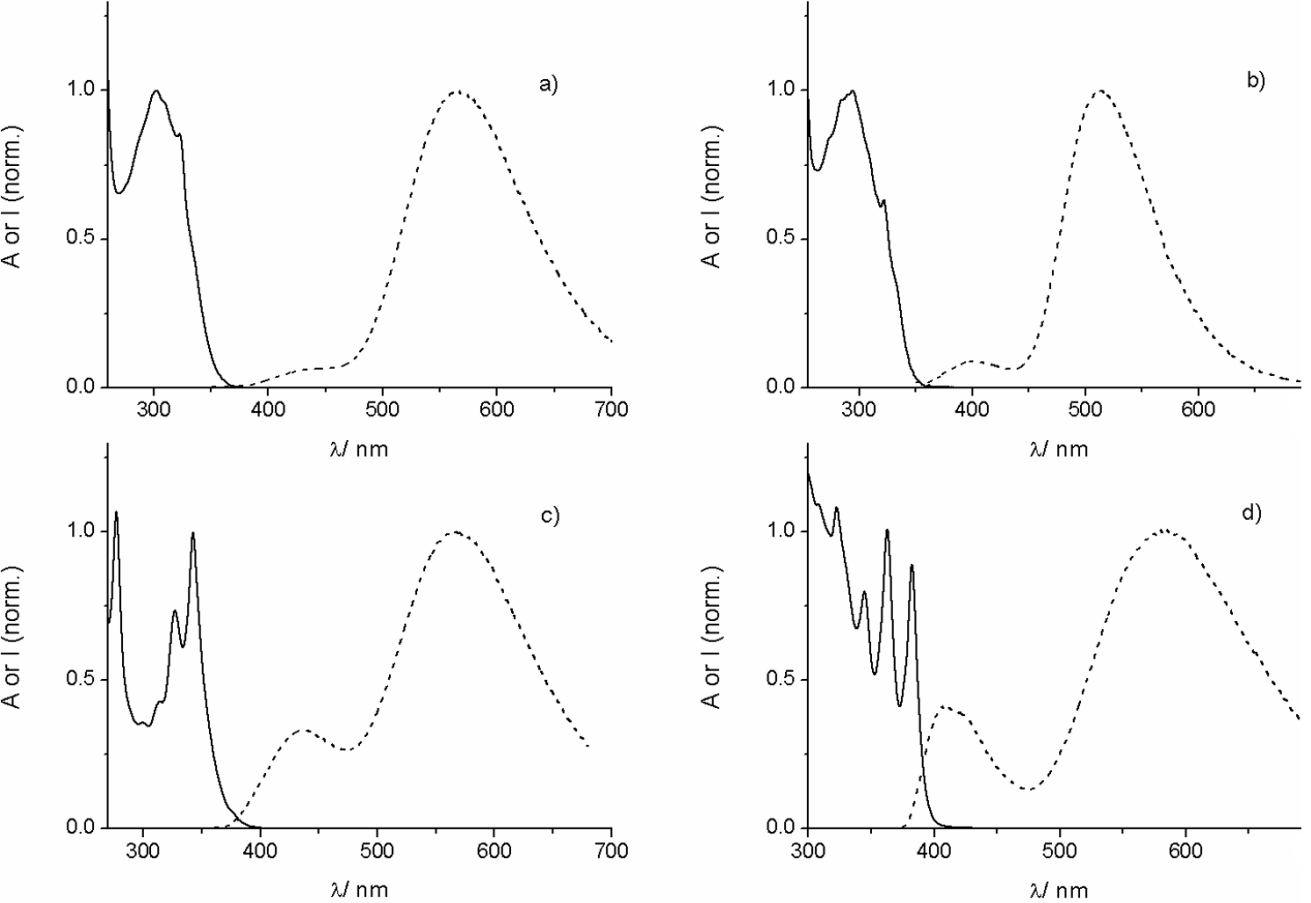
UV–vis absorption (solid line) and fluorescence (dashed line) spectra of a) **16**, b) **17**, c) **18**, and d) **19** in air-equilibrated acetonitrile (containing 0.4 vol % DMF as co-solvent).

The absorption and fluorescence properties of the herein investigated dyes **16**–**19** in air-equilibrated solutions, using three solvents (dichloromethane, acetonitrile, dimethyl sulfoxide), are summarized in Table 1. A first inspection of these data showed that the UV–vis absorption spectra feature the typical bands corresponding to their aromatic moieties (see Figure 2 for the spectra in acetonitrile). For example, for the dyes **18** and **19** π–π* transition bands in the wavelength range of 330–400 nm with characteristic vibronic fine structure were observed. Further, the dyes have a sharp peak at 322 nm that is assigned to the isoquinoline chromophore. The only exception is dye **18** where this peak is hidden under a strong absorption band corresponding to the pyrenyl moiety.

**Table 1:** UV–vis and fluorescence properties of the dyes **16**–**19** in various solvents.

	λ_abs,max_ (nm) [ε (M^−1^cm^−1^)]	λ_fluo,max_ (nm) SW/LW	*I*_LW_/*I*_SW_	Φ_fluo_	τ_fluo_ (ns) SW/LW

CH_2_Cl_2_					

**16**	303 [10800]	429/555	7.1	0.59	0.43/6.11
**17**	296 [11500]	397/512	7.1	0.17	0.16/3.96
**18**	345 [36900]	431/549	4.6	0.48	0.91/4.02
**19**	365 [6900]	409/551	2.3	0.30	0.57/5.22

CH_3_CN (0.4 vol % DMF as co-solvent)					

**16**	302 [10100]	437/565	15.7	0.48	0.40/6.03
**17**	294 [16000]	400/514	11.2	0.14	0.13/3.26
**18**	343 [33000]	435/565	3.0	0.35	0.39/4.74
**19**	363 [13300]	408/582	2.4	0.15	0.32/4.83

(CH_3_)_2_SO					

**16**	304 [10500]	451/577	3.7	0.41	0.72/4.91
**17**	296 [17400]	402/519	5.3	0.20	0.22/3.63
**18**	346 [29700]	444/569	1.8	0.47	0.55/4.81
**19**	366 [6500]	413/592	1.0	0.22	0.60/4.70

Most interesting are the fluorescence properties of the dyes (see spectra in Figure 2), which revealed a dual emission phenomenon (see ratio *I*_LW_/*I*_SW_ of the intensities *I* of the long-wavelength (LW) and short-wavelength (SW) emission band; Table 1). The monitoring of the emission corresponding to both bands yields identical excitation spectra which also coincide with the absorption spectra of the dyes. This underpins the authenticity of the emission signals. The appearance of the LW emission for all investigated dyes can be clearly linked to the presence of the boron-containing substituent. This follows from the observation that the corresponding arylisoquinolines without boron substitution feature only one blue-shifted emission band that is very similar to the SW band of the borylated dye, e.g., the non-borylated analogues of the dyes **17**, **18**, and **19** feature a single emission band with a maximum at 401, 442, and 420 nm, in acetonitrile, respectively. These are tentatively assigned to π–π* transitions of the variable aryl moiety. Interestingly, in tetrahydrofuran, containing oxygen as donor atom, only the SW emission band is seen, i.e., λ_fluo,max_ = 409 nm (**16**), 402 nm (**17**), 426 nm (**18**), 425 nm (**19**). This points to the interpretation that the SW emission has its origin in a Lewis adduct between the boron center as acceptor and the solvent as donor. The maxima of the rather broad LW bands of the dyes are observed between 510 and 590 nm in acetonitrile, corresponding to maximal apparent Stokes shifts of ca. 190–270 nm. As demonstrated previously for other borylated arylisoquinoline dyes [37–38], the emission energy of the LW band is tightly linked with the redox potential of the aryl residue. Having in mind that the borylated naphthyl is present in all four dyes it is instructive to compare the oxidation potentials (*E*_ox_) of the additional aryl residues. This leads to the following order: naphthyl (*E*_ox_ = 1.70 V vs SCE in acetonitrile) > 4-methoxynaphthyl (1.38 V) > pyrenyl (1.16 V) > anthryl (1.09 V) [44]. On the one hand, the dye with the easiest oxidizable aromatic residue (dye **19**) has the most red-shifted emission maximum, being at 582 nm in acetonitrile. On the other hand, dye **17** with a naphthyl, that is harder to oxidize, shows the most blue-shifted LW emission (maximum at 514 nm in acetonitrile). The LW emission maxima of other dyes (**16** and **18**) are situated in between. These trends support that for the herein investigated dyes intramolecular charge-transfer (ICT) phenomena might play a role in the observation of the LW emission features. According to our previous observations the electron-acceptor moiety is likely constituted by the isoquinolinyl moiety [37–38], while the donor is related to the electronically variable aryl residue. Comparing the emission maxima of the dyes in the less polar dichloromethane with those in the highly polar dimethyl sulfoxide, additional trends can be seen. Thus, dye **17** shows only a slight bathochromic shift of the emission maximum on changing to the polar solvent (Δλ = +7 nm). However, dye **19** features a solvatofluorochromic effect of Δλ = +41 nm under the same conditions. The dyes **16** and **18** show somewhat smaller bathochromic shifts on increasing the solvent polarity (Δλ = +20–22 nm).

**Scheme 4:**
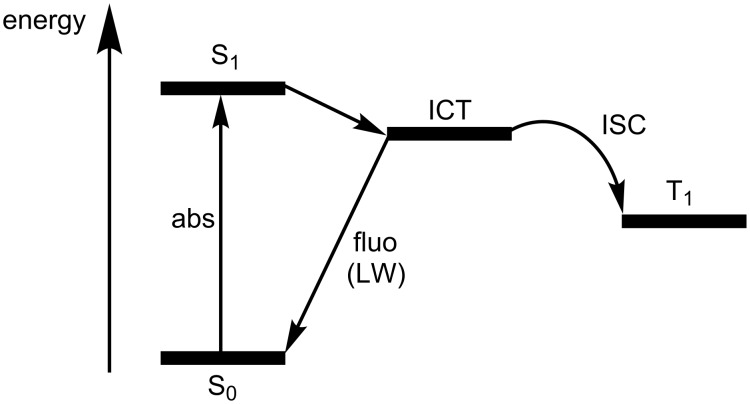
Jablonski diagram representing the photophysical processes in the dyes **16**–**19**.

Regarding the fluorescence quantum yields (Φ_fluo_) of the dyes, the highest values were determined for the compounds **16** and **18**, being in the range of 0.35–0.59 in the investigated solvents. The dyes **17** and **19** show smaller values for Φ_fluo_ (ca. 0.15–0.30). The fluorescence lifetime of the SW emission was measured as 300–900 ps, being in some cases very close to the resolution limit of our time-correlated single-photon-counting setup. The LW emission showed considerably longer lifetimes in the 3–6 ns range. The photophysical behavior of the dyes is tentatively summarized in Scheme 4.

### Laser-flash photolysis

**Figure 3:**
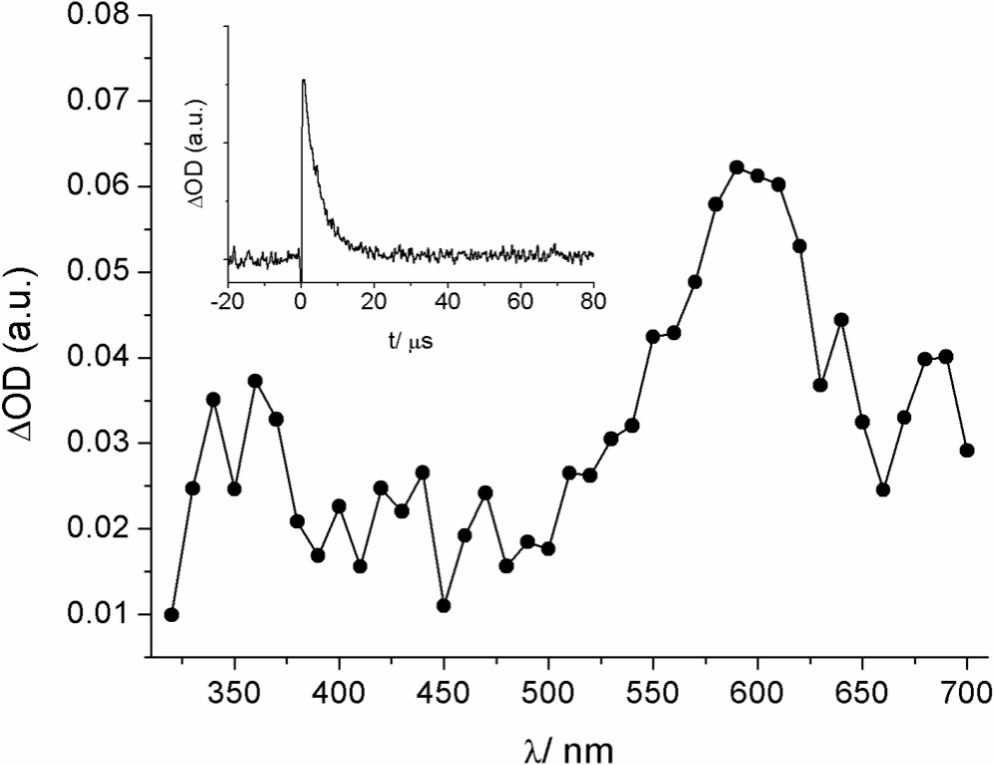
Transient absorption spectrum (600 ns delay) of dye **17** in nitrogen-purged acetonitrile on excitation at 308 nm. The inset shows the corresponding kinetics at 600 nm.

The photophysical characterization of the dyes **16**–**19** was completed by nanosecond laser-flash photolysis experiments in acetonitrile [45]. The laser excitation (λ_exc_ = 308 nm) of the dyes **16** and **17** in nitrogen-purged solution yielded transient absorption spectra with a broad band at λ_max_ = 610 and 600 nm, respectively (see Figure 3 for dye **17**). These transients showed lifetimes in the microsecond range (τ_T_ = 4.2 μs (**16**) and 4.4 μs (**17**)), were efficiently quenched by oxygen (bimolecular quenching constant *k*_q_ ca. 1.1–1.2 × 10^9^ M^−1^s^−1^), and led to the energy-transfer triplet-sensitization of β-carotene (observation of the triplet–triplet absorption band at 520 nm). The experimental results corroborate the assignment of the transients to excited triplet states of **16** and **17**. Noteworthy, the dyes **18** and **19** are characterized by distinct transient absorption spectra (excitation at λ_exc_ = 355 nm) with signals at shorter wavelengths. Based on the microsecond lifetime (τ_T_ = 3.1 μs (**18**) and 2.4 μs (**19**)), oxygen quenching (*k*_q_ ca. 2.9-3.1 × 10^9^ M^−1^s^−1^), and β-carotene triplet sensitization experiments the signals at 410 nm (dye **18**) and 430 nm (dye **19**) were assigned to excited triplet states as well. An additional signal at 470 nm for dye **18** is insensitive to oxygen and was tentatively attributed to the formation of a pyrene-based radical cation, resulting from photoionization [46].

### Interaction with fluoride anions

**Figure 4:**
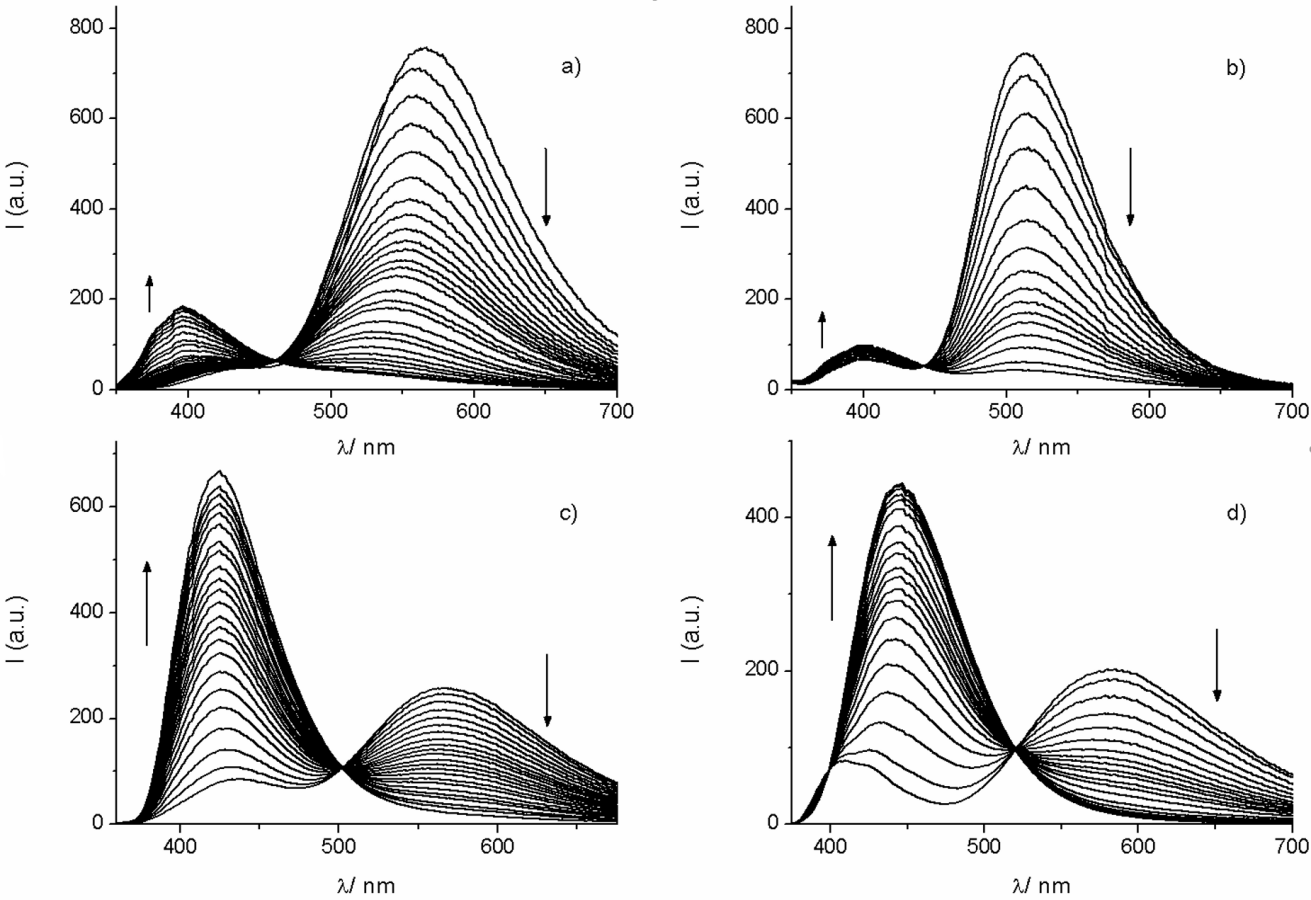
Fluorescence titrations of the dyes (ca. 4–11 μM) with Bu_4_NF in acetonitrile. a) **16** (up to 156 equiv F^−^), b) **17** (up to 40 equiv F^−^), c) **18** (up to 152 equiv F^−^), d) **19** (up to 100 equiv F^−^).

The presence of the boronic acid ester moiety does not only contribute to significant changes in the fluorescence properties but constitutes also a potential binding motif for Lewis bases. In this context it is well established that the electron-deficient trivalent boron can bind anions, such as fluoride or cyanide, through interaction with the vacant 2p_π_ orbital [30]. In Figure 4 the fluorescence responses of the dyes **16**–**19** on the addition of tetra-*n*-butylammonium fluoride (Bu_4_NF) in acetonitrile are depicted. The dyes **16** and **17** show a strong fluorescence quenching of their LW bands, while the SW bands experience a slight increase. However, the situation for the dyes **18** and **19** is dramatically different. Here the LW band is substituted by a strong blue-shifted emission. This leads to a clear ratiometric behavior and a large dynamic response. The blue-shifted emission for the fluoroboronate Lewis adduct is in accordance with the observations made for donor solvents such as tetrahydrofuran (see above). As for the dyes **16** and **17**, also for **18** and **19** isoemissive points were noted. These observations corroborate the uniformity of the reaction with fluoride anions. The UV–vis absorption spectra show much smaller changes as compared to the fluorescence (not shown). However, also here isosbestic points were observed. The formation of the fluoroboronate complexes was corroborated by the detection of the corresponding mass peaks (see Supporting Information File 1). In addition, ^11^B NMR spectra, for the example of dye **17**, reveal that the boron changes from sp^2^ to sp^3^ hybridization on addition of 1 equiv F^−^; i.e., the ^11^B NMR signal shifts from 31.5 ppm to 7.0 ppm (see Supporting Information File 1). This is in line with the formation of the fluoroboronate complex, instead of unwanted processes such as protodeboronation which could be potentially caused by acid traces in Bu_4_NF. Noteworthy, the addition of other anions, such as bromide, iodide, or cyanide did not result in significant changes of the optical spectra of the dyes.

Fluorescence titrations yielded the formation constants for the respective 1:1 fluoroboronate complexes. The values are in the order of 10^4^ M^−1^ (1.6 × 10^4^ M^−1^ (**16**); 4.8 × 10^4^ M^−1^ (**17**); 2.6 × 10^4^ M^−1^ (**18**); 2.0 × 10^4^ M^−1^ (**19**)), which are very comparable to the constants that were obtained for related borylated arylisoquinoline dyes [37].

## Conclusion

The family of borylated arylisoquinoline dyes was extended by members that contain additional aryl substituents, leading to compounds with two stereogenic axes. The dyes show pronounced dual emission patterns with long-wavelength maxima close to 600 nm in polar solvents such as acetonitrile or dimethyl sulfoxide. The emission maxima of the long-wavelength band vary systematically with the electron-donor strength of the additional aryl residue (naphthyl, 4-methoxynaphthyl, pyrenyl, anthryl). This provides some hint that intramolecular charge-transfer phenomena are likely involved. Laser-flash photolysis studies provided insights into the existence of excited triplet states. The addition of fluoride anions led to pronounced fluorescence quenching effects, as the result of the formation of fluoroboronate complexes. In the case of the pyrenyl- and anthryl-substituted dyes a clear ratiometric behavior was noted. No quenching was seen for the addition of cyanide ions or bromide and chloride. This makes the new dyes selective fluorescent receptors for fluoride anions.

## Experimental

### General methods and materials

^1^H NMR spectra were recorded at 400 MHz or 500 MHz and ^13^C NMR spectra were recorded at 100 MHz or 125 MHz. Chloroform-*d* (CDCl_3_), acetone-*d*_6_ ((CD_3_)_2_CO) and benzene-*d*_6_ (C_6_D_6_) were used as solvents and the solvent peak was employed as reference. ^11^B NMR spectra were recorded with complete proton decoupling at 160 MHz, using BF_3_·Et_2_O (0.00 ppm for ^11^B NMR) as standard.

All chemical reactions were carried out in oven-dried Schlenk tubes under an argon atmosphere. Toluene, 1,4-dioxane, and methanol were purchased from Carlo Erba and were used as received. Anhydrous THF was obtained using Grubbs-type solvent drying columns. [Pd(PPh_3_)_4_], Pd_2_(dba)_3_, SPhos ligand, 1-chloroisoquinoline, and pinacolborane (HBpin) were supplied by Aldrich, [Ir(µ-OMe)(cod)]_2_ was from Strem Chemicals, and bis(pinacolate)diboron (B_2_pin_2_) was purchased from Frontier Scientific. All reagents were used as received. 1-Bromo-4-methoxynaphthalene (**1**) [47], 1-chloroanthracene (**4**) [48], and 1‐(tetrahydropyran‐2’‐yloxy)‐4‐bromonaphthalene (**6**) [49] were synthesized according to literature procedures. The solvents for the photophysical measurements were purchased from Aldrich (acetonitrile) or Scharlau (dichloromethane, dimethyl sulfoxide) and were of spectroscopic quality.

UV–vis absorption and corrected fluorescence spectra were measured with standard equipment (Shimadzu UV-1603 and Varian Cary Eclipse), using quartz cuvettes of 1 cm optical path length. The fluorescence quantum yields were determined with quinine sulfate as standard reference (Φ_fluo_ = 0.55 in 0.05 M H_2_SO_4_) [50–51]. The lifetimes were measured by time-correlated single-photon counting (Edinburgh instruments FLS 920).

Laser-flash photolysis experiments were performed using a XeCl excimer laser (λ_exc_ = 308 nm; 17 ns fwhm; 20 mJ/pulse). Alternatively, a Q-switched Nd:YAG laser (Quantel Brilliant, 355 nm, 5 ns fwhm, 15 mJ/pulse) was coupled to a mLFP-111 Luzchem miniaturized equipment. The concentration of **16**–**19** was kept in the range of 20–30 μM in acetonitrile. The solutions were air-equilibrated or bubbled for 30 min with N_2_ or O_2_ before acquisition. All the experiments were carried out at room temperature.

The detailed procedures for the synthesis of the precursors can be found in Supporting Information File 1. Below the borylation of the precursors **12**–**15** to yield the dyes **16**–**19** is described and the NMR characterization data of the dyes are given.

### General procedure for the Ir-catalyzed borylation – synthesis of the dyes **16**–**19**

Following the described procedure [42], a dried Schlenk tube was loaded with the substrate (**12**–**15**) and B_2_Pin_2_ (1 equiv). After three vacuum–argon cycles, 1 mL catalyst stock solution per 0.5 mmol substrate and pinacolborane (HBPin, 5 mol %) was added. The reaction mixture was stirred at 55 °C until quantitative consumption of the starting material. The mixture was cooled to room temperature, concentrated to dryness, and the crude product was purified by column chromatography (*n*-hexane/EtOAc mixtures).

Note: The catalyst stock solution (25 mL) was prepared by dissolving 2-pyridinecarboxaldehyde *N*,*N*-dibenzylhydrazone (37.6 mg, 0.125 mmol) and [Ir(µ-OMe)(cod)]_2_ (41 mg, 0.063 mmol) in dry THF. Sonication for one hour was used to facilitate dissolution. The resulting red-brown solution was kept under argon.

#### 1-(4'-Methoxy-3-(4,4,5,5-tetramethyl-1,3,2-dioxaborolan-2-yl)-[1,1'-binaphthalen]-4-yl)isoquinoline (**16**)

Following the above described general procedure for the Ir-catalyzed borylation starting from **12** (85 mg, 0.21 mmol) and after flash chromatography on silica gel (toluene/EtOAc 7:1), **16** was obtained as a light-yellow foam (70 mg, 62% yield). NMR spectra recorded at 25 °C showed a ca. 0.45:0.55 diastereomeric mixture of atropisomers. To simplify the spectra the measurements were undertaken at 80 °C. ^1^H NMR (400 MHz, C_6_D_6_, 80 °C) δ 8.80 (d, *J* = 5.6 Hz, 0.5H), 8.78 (d, *J* = 5.6 Hz, 0.5H), 8.60 (d, *J* = 8.0 Hz, 0.5H), 8.58 (d, *J* = 8.0 Hz, 0.5H), 8.46 (s, 0.5H), 8.44 (s, 0.5H), 7.70–7.47 (m, 5H), 7.34–7.26 (m, 2H), 7.10–6.99 (m, 4H), 6.62 (d, *J* = 7.2 Hz, 0.5H), 6.61 (d, *J* = 7.6 Hz, 0.5H), 3.61 (s, 3H), 0.80 (s, 6H), 0.69 (s, 3H), 0.65 (s, 3H) ppm, two proton signals were hidden under the C_6_D_6_ peak; ^13^C NMR (100 MHz, C_6_D_6_, 80 °C) δ 162.8, 156.1, 145.7, 145.6, 143.1, 138.9, 136.6, 135.5, 134.9 (br s), 133.5, 133.4, 131.9, 130.6, 129.5, 128.6, 127.4, 127.1, 126.8, 126.6, 126.2, 125.5, 125.4, 122.8, 122.5, 120.1, 119.7, 119.6, 104.4, 104.1, 83.4, 55.4, 24.6 ppm, *C*–B not observed; ^11^B NMR (128 MHz, C_6_D_6_) δ 32.0 ppm (br s); HRESIMS *m*/*z*: [M + Na]^+^ calcd. for C_36_H_32_BNNaO_3_, 560.2367; found, 560.2370.

#### 1-(3-(4,4,5,5-Tetramethyl-1,3,2-dioxaborolan-2-yl)-[1,1'-binaphthalen]-4-yl)isoquinoline (**17**)

Following the above described general procedure for the Ir-catalyzed borylation starting from **13** (95 mg, 0.25 mmol) and after flash chromatography on silica gel (*n*-hexane/EtOAc 4:1), **17** was obtained as light-yellow foam (105 mg, 83% yield). NMR spectra recorded at 25 °C showed a ca. 0.45:0.55 diastereomeric mixture of atropisomers. To simplify the spectra the measurements were undertaken at 80 °C. ^1^H NMR (500 MHz, C_6_D_6_, 80 °C) δ 8.77 (br s, 1H), 8.38 (s, 0.55H) 8.36 (s, 0.45H), 7.81–7.77 (m, 3H), 7.68–7.53 (m, 4.55H), 7.49 (d, *J* = 5.5 Hz, 1H), 7.39 (br s, 1.45H), 7.30 (br s, 1H), 7.25 (br s, 1H), 7.11–7.02 (m, 4H), 0.81 (s, 6H), 0.70 (s, 3H), 0.68 (s, 3H) ppm; ^13^C NMR (100 MHz, C_6_D_6_, 80 °C) δ 162.6, 145.7, 143.0, 139.5, 138.6, 136.5, 135.0, 134.4, 133.9, 133.3, 133.0, 130.5, 129.5, 128.7, 128.5, 127.9, 127.5, 127.1, 127.1, 126.7, 126.6, 126.3, 126.2, 126.1, 126.0, 125.8, 125.5, 119.6, 83.4, 24.5 ppm, *C*–B not observed; ^11^B NMR (128 MHz, C_6_D_6_) δ 31.3 ppm (br s); HREIMS *m*/*z*: [M]^+^ calcd. for C_35_H_30_BNO_2_, 507.2370; found, 507.2375.

#### 1-(4-(Pyren-1-yl)-2-(4,4,5,5-tetramethyl-1,3,2-dioxaborolan-2-yl)naphthalen-1-yl)isoquinoline (**18**)

Following the above described general procedure for the Ir-catalyzed borylation starting from **14** (114 mg, 0.25 mmol) and after flash chromatography on silica gel (*n*-hexane/EtOAc 5:1), **18** was obtained as a light-yellow foam (74 mg, 51% yield). NMR spectra recorded at 25 °C showed a ca. 0.45:0.55 diastereomeric mixture of atropisomers. To simplify the spectra the measurements were undertaken at 80 °C. ^1^H NMR (500 MHz, C_6_D_6_, 80 °C) δ 8.87 (d, *J* = 5.5 Hz, 0.55H), 8.85 (d, *J* = 5.5 Hz, 0.55H), 8.62 (s, 0.55H), 8.59 (s, 0.45H), 8.12 (d, *J* = 7.6 Hz, 0.55H), 8.08 (d, *J* = 9.1 Hz, 0.45H), 8.01–7.98 (m, 2H), 7.94–7.93 (m, 2H), 7.91–7.82 (m, 2H), 7.80–7.71 (m, 3H), 7.68–7.58 (m, 3H), 7.47 (d, *J* = 5.5 Hz, 1H), 7.26 (d, *J* = 8.1 Hz, 0.45H), 7.29 (d, *J* = 8.3 Hz, 0.55H), 7.12 (d, *J* = 7.4 Hz, 0.55H), 7.09 (d, *J* = 6.9 Hz, 0.45H), 7.06–7.00 (m, 3H), 0.79 (s, 6H), 0.65 (s, 2.7H), 0.62 (s, 3.3H) ppm; ^13^C NMR (100 MHz, C_6_D_6_, 80 °C) δ 162.7, 145.9, 143.3, 139.0, 136.8, 136.8, 136.6, 135.3, 133.7, 133.4, 132.2, 131.9, 131.9, 131.6, 130.7, 130.6, 129.6, 129.3, 127.3, 126.8, 126.6, 126.5, 126.4, 126.4, 126.2, 125.8–125.7, 125.5–125.3, 124.8, 119.6, 83.5, 24.5 ppm, *C*–B not observed; ^11^B NMR (128 MHz, C_6_D_6_) δ 32.0 ppm (br s); HREIMS [M]^+^ calcd. for C_41_H_32_BNO_2_, 581.2526; found, 581.2530.

#### 1-(4-(Anthracen-1-yl)-2-(4,4,5,5-tetramethyl-1,3,2-dioxaborolan-2-yl)naphthalen-1-yl)isoquinoline (**19**)

Following the above described general procedure for the Ir-catalyzed borylation starting from **15** (84 mg, 0.21 mmol) and after flash chromatography on silica gel (toluene/EtOAc 20:1), **19** was obtained as a yellow foam (100 mg, 72% yield). NMR spectra recorded at 25 °C showed a ca. 0.44:0.56 diastereomeric mixture of atropisomers. To simplify the spectra the measurements were undertaken at 80 °C. ^1^H NMR (500 MHz, C_6_D_6_, 80 °C) δ 8.81 (d, *J* = 5.6 Hz, 0.55H), 8.79 (d, *J* = 5.6 Hz, 0.45H) 8.54 (s, 0.55H), 8.49 (s, 0.45H), 8.44 (s, 0.45H), 8.33 (s, 1H), 8.26 (s, 0.55H), 8.00–7.93 (m, 1.45H), 7.81 (t, *J* = 8.6 Hz, 1H), 7.71–7.59 (m, 4H), 7.49 (d, *J* = 5.6 Hz, 1H), 7.41–7.27 (m, 3H), 7.19–7.10 (m, 1.55H), 7.05–6.97 (m, 4H), 0.80 (s, 2.7H), 0.79 (s, 3.3H), 0.69 (s, 2.7H), 0.64 (s, 3.3H) ppm; ^13^C NMR (125 MHz, C_6_D_6_, 80 °C) δ 162.6, 145.9, 145.9, 143.1, 143.0, 139.6, 139.4, 138.8, 136.5, 135.2, 135.1, 133.3, 133.2, 132.6, 132.5, 132.5, 132.4, 132.3, 132.2, 130.5, 130.4, 129.5, 129.5, 129.3, 129.2, 128.6, 128.5, 128.5, 128.3, 128.1, 127.9, 127.5, 127.3, 127.2, 127.2, 127.1, 126.9, 126.8, 126.8, 126.7, 126.7, 126.5, 126.3, 126.3, 126.1, 125.8, 125.6, 125.4, 125.2, 125.2, 119.6, 83.4, 24.5, 24.4 ppm, *C*–B not observed; ^11^B NMR (160 MHz, C_6_D_6_) δ 31.5 ppm (br s); HREIMS *m*/*z*: [M]^+^ calcd. for C_39_H_32_BNO_2_, 557.2526; found, 557.2508.

## Supporting Information

File 1 Additional synthetic procedures for **2**, **3**, **5**, and **7**–**15**, ^1^H and ^13^C NMR spectra of the dyes **16**–**19** and their precursors, ESIMS spectra and ^11^B NMR spectroscopy of fluoroboronate complexes, HPLC traces for the dyes **16**–**19**.

## References

[1] Frath D, Massue J, Ulrich G, Ziessel R (2014). Angew Chem.

[2] Ji L, Griesbeck S, Marder T B (2017). Chem Sci.

[3] Loudet A, Burgess K (2007). Chem Rev.

[4] Ulrich G, Ziessel R, Harriman A (2008). Angew Chem.

[5] Amarne H, Baik C, Murphy S K, Wang S (2010). Chem – Eur J.

[6] Pais V F, Alcaide M M, López-Rodríguez R, Collado D, Nájera F, Pérez-Inestrosa E, Álvarez E, Lassaletta J M, Fernández R, Ros A (2015). Chem – Eur J.

[7] Shaikh A C, Ranade D S, Thorat S, Maity A, Kulkarni P P, Gonnade R G, Munshi P, Patil N T (2015). Chem Commun.

[8] Liu K, Lalancette R A, Jäkle F (2017). J Am Chem Soc.

[9] Vanga M, Lalancette R A, Jäkle F (2019). Chem – Eur J.

[10] Santos F M F, Rosa J N, Candeias N R, Parente Carvalho C, Matos A I, Ventura A E, Florindo H F, Silva L C, Pischel U, Gois P M P (2016). Chem – Eur J.

[11] Alcaide M M, Santos F M F, Pais V F, Carvalho J I, Collado D, Pérez-Inestrosa E, Arteaga J F, Boscá F, Gois P M P, Pischel U (2017). J Org Chem.

[12] Frath D, Azizi S, Ulrich G, Retailleau P, Ziessel R (2011). Org Lett.

[13] Urban M, Durka K, Jankowski P, Serwatowski J, Luliński S (2017). J Org Chem.

[14] Wakamiya A, Taniguchi T, Yamaguchi S (2006). Angew Chem.

[15] Rao Y-L, Wang S (2011). Inorg Chem.

[16] Li D, Zhang H, Wang Y (2013). Chem Soc Rev.

[17] Coskun A, Akkaya E U (2006). J Am Chem Soc.

[18] Bozdemir O A, Guliyev R, Buyukcakir O, Selcuk S, Kolemen S, Gulseren G, Nalbantoglu T, Boyaci H, Akkaya E U (2010). J Am Chem Soc.

[19] Niu L-Y, Guan Y-S, Chen Y-Z, Wu L-Z, Tung C-H, Yang Q-Z (2012). J Am Chem Soc.

[20] Zhang X, Xiao Y, Qi J, Qu J, Kim B, Yue X, Belfield K D (2013). J Org Chem.

[21] Zheng Q, Xu G, Prasad P N (2008). Chem – Eur J.

[22] Han J, Loudet A, Barhoumi R, Burghardt R C, Burgess K (2009). J Am Chem Soc.

[23] Kowada T, Maeda H, Kikuchi K (2015). Chem Soc Rev.

[24] Kolemen S, Işık M, Kim G M, Kim D, Geng H, Buyuktemiz M, Karatas T, Zhang X-F, Dede Y, Yoon J (2015). Angew Chem.

[25] Bachollet S P J T, Volz D, Fiser B, Münch S, Rönicke F, Carrillo J, Adams H, Schepers U, Gómez-Bengoa E, Bräse S (2016). Chem – Eur J.

[26] Frath D, Didier P, Mély Y, Massue J, Ulrich G (2017). ChemPhotoChem.

[27] Kubo Y, Yamamoto M, Ikeda M, Takeuchi M, Shinkai S, Yamaguchi S, Tamao K (2003). Angew Chem.

[28] Melaimi M, Gabbaï F P (2005). J Am Chem Soc.

[29] Hudnall T W, Kim Y-M, Bebbington M W P, Bourissou D, Gabbaï F P (2008). J Am Chem Soc.

[30] Wade C R, Broomsgrove A E J, Aldridge S, Gabbaï F P (2010). Chem Rev.

[31] Hudson Z M, Liu X-Y, Wang S (2011). Org Lett.

[32] Bai D-R, Liu X-Y, Wang S (2007). Chem – Eur J.

[33] Proń A, Zhou G, Norouzi-Arasi H, Baumgarten M, Müllen K (2009). Org Lett.

[34] Pan H, Fu G-L, Zhao Y-H, Zhao C-H (2011). Org Lett.

[35] Bonn A G, Wenger O S (2015). J Org Chem.

[36] Griesbeck S, Zhang Z, Gutmann M, Lühmann T, Edkins R M, Clermont G, Lazar A N, Haehnel M, Edkins K, Eichhorn A (2016). Chem – Eur J.

[37] Pais V F, El-Sheshtawy H S, Fernández R, Lassaletta J M, Ros A, Pischel U (2013). Chem – Eur J.

[38] Pais V F, Lineros M, López-Rodríguez R, El-Sheshtawy H S, Fernández R, Lassaletta J M, Ros A, Pischel U (2013). J Org Chem.

[39] Pais V F, Lassaletta J M, Fernández R, El-Sheshtawy H S, Ros A, Pischel U (2014). Chem – Eur J.

[40] Domínguez Z, López-Rodríguez R, Álvarez E, Abbate S, Longhi G, Pischel U, Ros A (2018). Chem – Eur J.

[41] Billingsley K L, Barder T E, Buchwald S L (2007). Angew Chem.

[42] Ros A, Estepa B, López-Rodríguez R, Álvarez E, Fernández R, Lassaletta J M (2011). Angew Chem.

[43] Zhu L, Shabbir S H, Gray M, Lynch V M, Sorey S, Anslyn E V (2006). J Am Chem Soc.

[44] Montalti M, Credi A, Prodi L (2006). Handbook of Photochemistry.

[45] Boscá F, Cuquerella M C, Pais V F, Ros A, Pischel U (2018). ChemPhotoChem.

[46] Hara M, Tojo S, Kawai K, Majima T (2004). Phys Chem Chem Phys.

[47] Carreño M C, García-Ruano J L, Sanz G, Toledo M A, Urbano A (1995). J Org Chem.

[48] Moursounidis J, Wege D (1988). Aust J Chem.

[49] Weimar M, Dürner G, Bats J W, Göbel M W (2010). J Org Chem.

[50] Melhuish W H (1960). J Phys Chem.

[51] Melhuish W H (1961). J Phys Chem.

