# Supplementary material for: Arylisoquinoline-derived organoboron dyes with a triaryl skeleton show dual fluorescence
Source: Beilstein J Org Chem. 2019 Nov 4;15:2612–22. doi: 10.3762/bjoc.15.254 (PMC6880824; doi:10.3762/bjoc.15.254)
Supplement: File 1 — Additional synthetic procedures for 2, 3, 5, and 7–15, 1H and 13C NMR spectra of the dyes 16–19 and their precursors, ESIMS spectra and 11B NMR spectroscopy of fluoroboronate complexes, HPLC traces for the dyes 16–19. [file Beilstein_J_Org_Chem-15-2612-s001.pdf]

BEILSTEIN JOURNAL OF ORGANIC CHEMISTRY

Supporting Information

for

Arylisoquinoline-derived organoboron dyes with a triaryl skeleton show dual fluorescence

Vânia F. Pais, Tristan Neumann, Ignacio Vayá, M. Consuelo Jiménez, Abel Ros and Uwe Pischel

Beilstein J. Org. Chem. **2019,** *15,* 2612–2622. doi:10.3762/bjoc.15.254

Additional synthetic procedures for 2, 3, 5, and 7–15, ^{1}H and ^{13}C NMR spectra of the dyes 16–19 and their precursors, ESIMS spectra and ^{11}B NMR spectroscopy of fluoroboronate complexes, HPLC traces for the dyes 16–19

License and Terms: This is a supporting information file under the terms of the Creative Commons Attribution License (http://creativecommons.org/licenses/by/4.0). Please note that the reuse, redistribution and reproduction in particular requires that the authors and source are credited.
The license is subject to the *Beilstein Journal of Organic Chemistry* terms and conditions: (https://www.beilstein-journals.org/bjoc)

Table of Contents

1. Synthesis S3
General procedure for the synthesis of **2** and **3** S3
4-Methoxy-1,1'-binaphthalene (**2**) S3
1-(4-Methoxynaphthalen-1-yl)pyrene (**3**) S3
1-(4-Methoxynaphthalen-1-yl)anthracene (**5**) S4
4'-Methoxy-[1,1'-binaphthalen]-4-ol (**7**) S5
4'-Methoxy-[1,1'-binaphthalen]-4-yl trifluoromethanesulfonate (**8**) S6
General procedure for the synthesis of triflates **9-11** S7
[1,1'-Binaphthalen]-4-yl trifluoromethanesulfonate (**9**) S7
4-(Pyren-1-yl)naphthalen-1-yl trifluoromethanesulfonate (**10**) S8
4-(Anthracen-1-yl)naphthalen-1-yl trifluoromethanesulfonate (**11**) S8
General procedure for the synthesis of substrates **12-15** S9
1-(4'-Methoxy-[1,1'-binaphthalen]-4-yl)isoquinoline (**12**) S9
1-([1,1'-Binaphthalen]-4-yl)isoquinoline (**13**) S10
1-(4-(Pyren-1-yl)naphthalen-1-yl)isoquinoline (**14**) S11
1-(4-(Anthracen-1-yl)naphthalen-1-yl)isoquinoline (**15**) S11
2. ^{1}H and ^{13}C NMR spectra (Figures S1–S40) S13–S36
3. HPLC traces for the dyes 16-19 (Figures S41–S44) S37–S38
4. Formation of fluoroboronate complexes – ESIMS and ^{11}B NMR (Figures S45–S49) S39–S41

1. Synthesis

General procedure for the synthesis of 2 and 3

A dried Schlenk tube flask was charged with $[Pd(PPh_3)_4]$ (3 mol %), Na_2CO_3 (2 equiv/mmol), 1-bromo-4-methoxynaphthalene (1–2 mmol), and the corresponding boronic acid (1.2 equiv). After three vacuum–argon cycles, toluene (1 mL/mmol substrate), methanol (0.2 mL/mmol substrate), and water (0.25 mL/mmol substrate) were added with the aid of a syringe. The reaction mixture was heated to 110 °C for 4 hours, then cooled to room temperature, diluted with CH_2Cl_2 and finally filtered through celite. The organic phase was concentrated to dryness and the crude products were purified by column chromatography (*n*-hexane/EtOAc mixtures).

4-Methoxy-1,1'-binaphthalene (2)

Following the general procedure (see above) and starting from 2 mmol 1-bromo-4-methoxynaphthalene (**1**), flash chromatography on silica gel (*n*-hexane/EtOAc 50:1) gave **2** (441 mg, 78% yield) as an amorphous white solid. ^{1}H NMR (400 MHz, $CDCl_3$): δ 8.39 (d, 1H, J = 8.4 Hz), 7.95 (d, 1H, J = 8.0 Hz), 7.59 (t, 1H, J = 7.6 Hz), 7.50-7.28 (m, 9H), 6.95 (d, 1H, J = 7.6 Hz), 4.10 (s, 3H) ppm. ^{13}C NMR (100 MHz, $CDCl_3$): δ 155.2, 138.6, 133.7, 133.5, 133.2, 130.6, 128.1, 128.1, 127.7, 127.7, 126.7, 126.4, 126.3, 125.8, 125.7, 125.4, 125.4, 125.1, 122.0, 103.3, 55.6 ppm. HRMS(EI) calcd. for $C_{21}H_{16}O$ (M^+) 284.1201. Found 284.1209.

1-(4-Methoxynaphthalen-1-yl)pyrene (3)

Following the general procedure (see above) starting from 1 mmol 1-bromo-4-methoxynaphthalene (**1**), flash chromatography on silica gel (*n*-hexane/EtOAc

50:1) gave **3** (310 mg, 87% yield) as an amorphous yellow solid. ^{1}H NMR (400 MHz, $CDCl_3$): δ 8.43 (d, 1H, *J* = 8.4 Hz), 8.28 (d, 1H, *J* = 7.6 Hz), 8.22 (d, 1H, *J* = 7.6 Hz), 8.18-8.12 (m, 3H), 8.03-7.99 (m, 2H), 7.89 (d, 1H, *J* = 9.2 Hz), 7.71 (d, 1H, *J* = 9.2 Hz), 7.52 (d, 1H, *J* = 8.0 Hz), 7.50 (td, 1H, *J* = 6.8, 1.6 Hz), 7.36 (d, 1H, *J* = 8.0 Hz), 7.30 (td, 1H, *J* = 6.8, 1.2 Hz), 7.01 (d, 1H, *J* = 8.0 Hz), 4.13 (s, 3H) ppm. ^{13}C NMR (100 MHz, $CDCl_3$): δ 155.3, 136.1, 133.9, 131.4, 131.0, 130.7, 130.2, 128.8, 128.3, 127.4, 127.3, 127.2, 126.6, 126.4, 126.0, 125.9, 125.5, 125.2, 125.0, 124.9, 124.8, 124.8, 124.5, 122.1, 103.4, 55.6 ppm. HRMS(EI) calcd. for $C_{27}H_{18}O$ (M^+) 358.1358. Found 358.1360.

1-(4-Methoxynaphthalen-1-yl)anthracene (5)

A dried Schlenk tube flask was charged with Pd_2dba_3 (1 mol %, 23 mg), SPhos (8 mol %, 76 mg), bis(pinacolato)diboron (2.4 mmol, 610 mg), 1-chloroanthracene (**4**, 2.4 mmol, 509 mg), and potassium acetate (4.1 mmol, 392 mg). After three vacuum-argon cycles, 1,4-dioxane (8 mL) was added with the aid of a syringe. The reaction mixture was heated to 110 °C for 5 hours. At this point the 1-bromo-4-methoxynaphthalene (**1**, 2 mmol, 470 mg) in 1,4-dioxane (2.4 mL) and 5 M K_3PO_4 (aq.) (2 mL) were added. The reaction mixture was heated to 110 °C overnight, then cooled to room temperature, and diluted with water (10 mL) and EtOAc (10 mL). The organic phase was separated and the aqueous phase was extracted with EtOAc (3 × 10 mL). The combined organic phase was dried over anhydrous $MgSO_4$ and the solvent was removed by rotary evaporation. The crude product was purified by column chromatography (*n*-hexane/EtOAc 100:1) to afford **5** (547 mg, 82% yield) as a light-yellow foam. ^{1}H NMR (400 MHz, $CDCl_3$): δ 8.53 (s, 1H), 8.43 (d, 1H, *J* = 8.8 Hz), 8.11 (d, 1H, *J* =

8.4 Hz), 8.02 (s, 1H), 8.01 (d, 1H, J = 6.0 Hz), 7.68 (d, 1H, J = 8.8 Hz), 7.58 (dd, 1H, J = 8.4, 6.8 Hz), 7.51-7.39 (m, 5H), 7.35-7.28 (m, 2H), 6.99 (d, 1H, J = 7.6 Hz), 4.14 (s, 3H) ppm. ^{13}C NMR (100 MHz, $CDCl_3$): δ 155.3, 138.7, 133.9, 131.9, 131.8, 131.6, 131.5, 130.8, 128.6, 128.0, 127.8, 127.8, 127.5, 126.5, 126.4, 126.4, 125.6, 125.5, 125.5, 125.2, 125.1, 125.0, 122.0, 103.5, 55.6 ppm. HRMS(EI) calcd. for $C_{25}H_{18}O$ (M^+) 334.1358. Found 334.1362.

4'-Methoxy-[1,1'-binaphthalen]-4-ol (7)

A dried Schlenk tube was charged with Pd_2dba_3 (1 mol%, 9.6 mg), SPhos (8 mol %, 32 mg), bis(pinacolato)diboron (1.1 mmol, 280 mg), 1-bromo-4-methoxynaphthalene (**1**, 1.1 mmol, 260 mg), and potassium acetate (1.7 mmol, 163 mg). After three vacuum–argon cycles, 1,4-dioxane (3 mL) was added with the aid of a syringe. The reaction mixture was heated to 110 °C for 5 hours. At this point 1-tetrahydropyran-2’-yloxy-4-bromonaphthalene (**6**, 1 mmol, 306 mg) in 1,4-dioxane (1 mL) and 5 M K_3PO_4 (aq.) (1 mL) were added. The reaction mixture was heated to 110 °C overnight, then cooled to room temperature, and diluted with H_2O (10 mL) and EtOAc (10 mL). The organic phase was separated and the aqueous phase was extracted with EtOAc (3 $\times$ 10 mL). The combined organic phase was dried over anhydrous $MgSO_4$ and the solvent was removed by rotary evaporation. The crude was dissolved in MeOH/CH_2Cl_2 10:1 (13 mL), treated with TsOH·H_2O (0.055 equiv, 11 mg) and after 90 min reaction time the reaction was quenched with $NaHCO_3$ (aq. sat., 10 mL). The organic phase was separated and the aqueous phase was extracted with CH_2Cl_2 (3 $\times$ 10 mL). The combined organic phase was dried over anhydrous $MgSO_4$ and the crude product was purified by flash chromatography on silica gel (n-hexane/EtOAc 5:1$\rightarrow$1:1). This procedure

gave **7** (210 mg, 70% yield) as an amorphous white solid. ^{1}H NMR (400 MHz, $(CD_3)_2CO$): δ 9.24 (br s, 1H), 8.41 (d, 1H, J = 8.4 Hz), 8.37 (d, 1H, J = 8.4 Hz), 7.52-7.47 (m, 2H), 7.40 (d, 1H, J = 8.0 Hz), 7.38-7.29 (m, 5H), 7.10 (d, 1H, J = 8.0 Hz), 7.09 (d, 1H, J = 8.0 Hz), 4.13 (s, 3H) ppm. ^{13}C NMR (100 MHz, $(CD_3)_2CO$): δ 155.8, 153.7, 135.2, 135.0, 131.7, 130.4, 129.2, 129.0, 127.1, 127.1, 127.0, 126.9, 126.4, 125.8, 125.8, 125.3, 123.2, 122.8, 108.5, 104.5, 56.0 ppm. HRMS(EI) calcd. for $C_{21}H_{16}O_2$ (M^+) 300.1150. Found 300.1145.

4'-Methoxy-[1,1'-binaphthalen]-4-yl trifluoromethanesulfonate (8)

A solution of **7** (300 mg, 1 mmol) in dry CH_2Cl_2 (20 mL), containing DMAP as catalyst, was cooled to 0 °C and dry pyridine (1.2 equiv) and Tf_2O (1.2 equiv) were carefully added. The reaction mixture was stirred overnight at room temperature and then quenched with water (10 mL). The organic phase was separated and the aqueous solution was extracted with CH_2Cl_2 (3 × 15 mL). The combined organic phase was dried over anhydrous $MgSO_4$, filtered, and concentrated to dryness. The crude product was purified by flash chromatography on silica gel (*n*-hexane/EtOAc 15:1). This procedure gave **8** (372 mg, 86% yield) as a light-brown foam. ^{1}H NMR (400 MHz, $CDCl_3$): δ 8.39 (d, 1H, J = 8.4 Hz), 8.18 (d, 1H, J = 8.4 Hz), 7.64 (t, 1H, J = 8.0 Hz), 7.57 (d, 1H, J = 8.0 Hz), 7.51-7.48 (m, 3H), 7.42-7.32 (m, 3H), 7.26 (d, 1H, J = 8.4 Hz), 6.95 (d, 1H, J = 8.0 Hz), 4.10 (s, 3H) ppm. ^{13}C NMR (100 MHz, $CDCl_3$): δ 155.7, 145.2, 139.6, 134.6, 133.4, 128.9, 128.0, 127.7, 127.3, 127.3, 127.2, 126.8, 126.4, 125.9, 125.5, 125.3, 122.2, 120.9, 118.8 (q, $J_{C,F}$ = 318 Hz), 117.3, 103.3, 55.6 ppm. HRMS(EI) calcd. for $C_{22}H_{15}F_3O_4S$ (M^+) 432.0643. Found 432.0645.

General procedure for the synthesis of triflates 9–11

A dried Schlenk tube was charged with **2**, **3** or **5** (1 equiv) and after three vacuum–argon cycles, dry CH_2Cl_2 (3 mL/mmol substrate) was added. The reaction mixture was cooled to 0 °C and BBr_3 (1.1 equiv) was added dropwise. Then the mixture was allowed to warm to room temperature and stirred for 4 hours. After completion of the reaction the mixture was diluted with CH_2Cl_2 (20 mL) and washed with a saturated aqueous $NaHCO_3$ solution. The organic phase was dried over anhydrous $MgSO_4$, filtered, and concentrated to dryness. The crude product was used in the next reaction step without further purification.

A solution in dry CH_2Cl_2 (20 mL/mmol substrate), containing the reaction crude from the previous reaction and DMAP (cat.), was cooled to 0 °C and dry pyridine (1.2 equiv) and Tf_2O (1.2 equiv) were carefully added. The reaction mixture was stirred overnight at room temperature and then quenched with water (10 mL). The organic phase was separated and the aqueous solution was extracted with CH_2Cl_2 (3 × 15 mL). The combined organic phase was dried over anhydrous $MgSO_4$, filtered, and concentrated to dryness. The crude was purified by column chromatography on silica gel (*n*-hexane/EtOAc mixtures).

[1,1'-Binaphthalen]-4-yl trifluoromethanesulfonate (9)

Following the above described general procedure starting from **2** (440 mg, 1.55 mmol) and after flash chromatography on silica gel (*n*-hexane/EtOAc 10:1), **9** was obtained as a white foam (490 mg, 79% yield over two steps). ^1H NMR (400 MHz, $CDCl_3$): δ 8.20 (d, 1H, *J* = 8.8 Hz), 7.99 (d, 1H, *J* = 9.2 Hz), 7.97 (d, 1H, *J* = 9.6 Hz), 7.67-7.59 (m, 3H), 7.52-7.39 (m, 5H), 7.32 (d, 2H, *J* = 8.0 Hz) ppm. ^{13}C NMR (100 MHz, $CDCl_3$): δ 145.3, 139.4, 136.8, 134.3, 133.5, 132.5, 128.5, 128.3,

127.9, 127.7, 127.4, 127.1, 127.0, 126.4, 126.3, 126.1, 126.0, 125.3, 120.9, 118.8 (q, $J_{C,F}$ = 317 Hz), 117.3 ppm. HRMS(EI) calcd. for $C_{21}H_{13}F_3O_3S$ (M^+) 402.0537. Found 402.0540.

4-(Pyren-1-yl)naphthalen-1-yl trifluoromethanesulfonate (10)

Following the above described general procedure starting from **3** (310 mg, 0.87 mmol) and after flash chromatography on silica gel (*n*-hexane/EtOAc 20:1), **10** was obtained as a light-brown foam (300 mg, 72% yield over two steps). ^{1}H NMR (400 MHz, $CDCl_3$): δ 8.30 (d, 1H, *J* = 7.6 Hz), 8.26-8.24 (m, 2H), 8.17-8.16 (m, 3H), 8.04 (t, 1H, *J* = 7.6 Hz), 7.99 (d, 1H, *J* = 7.6 Hz), 7.92 (d, 1H, *J* = 9.2 Hz), 7.69-7.62 (m, 3H), 7.58 (d, 1H, *J* = 9.2 Hz), 7.45 (d, 1H, *J* = 8.0 Hz), 7.38 (td, 1H, *J* = 6.8, 0.8 Hz) Ppm. ^{13}C NMR (100 MHz, $CDCl_3$): δ 145.4, 139.8, 134.4, 134.0, 131.4, 131.2, 130.9, 129.8, 128.2, 127.9, 127.8, 127.6, 127.5, 127.4, 127.2, 126.5, 126.2, 125.5, 125.3, 125.1, 124.7, 124.6, 124.5, 121.0, 118.8 (q, $J_{C,F}$ = 318 Hz), 117.3 ppm. HRMS(EI) calcd. for $C_{27}H_{15}F_3O_3S$ (M^+) 476.0694. Found 476.0691.

4-(Anthracen-1-yl)naphthalen-1-yl trifluoromethanesulfonate (11)

Following the above described general procedure starting from **5** (1.2 mmol, 400 mg) and after flash chromatography on silica gel (*n*-hexane/EtOAc 20:1), **11** was obtained as a light-yellow foam (320 mg, 59% yield over two steps). ^{1}H NMR (400 MHz, $CDCl_3$): δ 8.55 (s, 1H), 8.24 (d, 1H, *J* = 8.4 Hz), 8.15 (d, 1H, *J* = 8.6 Hz), 8.03 (d, 1H, *J* = 8.4 Hz), 7.89 (s, 1H), 7.71 (d, 1H, *J* = 8.6 Hz), 7.65 (d, 2H, *J* = 8.2 Hz), 7.61-7.58 (m, 2H), 7.51-7.45 (m, 3H), 7.39-7.36 (m, 2H) ppm. ^{13}C NMR (100 MHz, $CDCl_3$): δ 145.4, 139.5, 136.9, 134.4, 131.8, 131.7, 131.6, 131.1,

128.9, 128.4, 127.9, 127.8, 127.5, 127.5, 127.1, 127.1, 126.7, 126.5, 125.8, 125.5, 125.0, 124.7, 121.0, 118.9 (q, $J_{C,F}$ = 321 Hz), 117.4 ppm. HRMS(EI) calcd. for $C_{25}H_{15}F_3O_3S$ (M^+) 452.0694. Found 452.0681.

General procedure for the synthesis of substrates 12–15

The synthesis was performed in a similar manner as described in [38]. A dried Schlenk tube was charged with Pd_2dba_3 (1 mol %), SPhos (8 mol %), bis(pinacolato)diboron (1 equiv), **8-11** (1 equiv), and potassium acetate (1.7 equiv). After three vacuum-argon cycles, 1,4-dioxane (3.5 mL/mmol substrate) was added with the aid of a syringe. Then the reaction mixture was heated to 110 °C for 4–6 hours. At this point 1-chloroisoquinoline (0.83 equiv) in 1,4-dioxane (1 mL/mmol substrate) and 5 M K_3PO_4 (aq) (0.4 mL/mmol substrate) were added with the aid of a syringe. The reaction mixture was heated to 110 °C overnight, then cooled to room temperature, and diluted with water (10 mL) and EtOAc (10 mL). The organic phase was separated and the aqueous phase was extracted with EtOAc (3 × 10 mL). The combined organic phase was dried over anhydrous $MgSO_4$ and the crude product was purified by column chromatography (*n*-hexane/EtOAc mixtures).

1-(4'-Methoxy-[1,1'-binaphthalen]-4-yl)isoquinoline (12)

Following the general procedure starting from **8** (285 mg, 0.66 mmol) and after flash chromatography on silica gel (*n*-hexane/EtOAc 3:1), **12** was obtained as a light-brown foam (100 mg, 44% yield). NMR spectra recorded at 25 °C showed a ca*.* 0.45:0.55 diastereomeric mixture of atropisomers. To simplify the spectra the measurements were undertaken at 80 °C. ^1H NMR (400 MHz, C_6D_6, 80 °C): δ

8.74 (d, 1H, *J* = 5.6 Hz), 8.59 (d, 1H, *J* = 8.0 Hz), 7.86-7.70 (m, 4H), 7.63 (d, 1H, *J* = 6.8 Hz), 7.58-7.47 (m, 3H), 7.39 (d, 1H, *J* = 5.6 Hz), 7.34 (t, 1H, *J* = 7.6 Hz), 7.29 (t, 1H, *J* = 7.6 Hz), 7.08-7.06 (m, 4H), 6.66 (d, 1H, *J* = 8.0 Hz), 3.64 (s, 3H) ppm. ^{13}C NMR (100 MHz, C_6D_6, 80 °C): δ 161.4, 156.1, 143.3, 140.1, 138.0, 137.0, 137.0, 134.7, 134.3, 133.5, 131.6, 129.9, 129.1, 128.6, 127.9, 127.5, 127.3, 127.1, 127.1, 126.6, 126.4, 126.2, 125.5, 122.7, 120.1, 104.1, 55.4 ppm, some carbons peaks were hidden under the C_6D_6 peak. HRMS(ESI) calcd. for $C_{30}H_{22}NO$ (M + H^+) 412.1696. Found 412.1684.

1-([1,1'-Binaphthalen]-4-yl)isoquinoline (13)

Following the general procedure starting from **9** (241 mg, 0.6 mmol) and after flash chromatography on silica gel (*n*-hexane/EtOAc 7:1→3:1), **13** was obtained as a light-yellow foam (130 mg, 68% yield). NMR spectra recorded at 25 °C showed a ca*.* 0.45:0.55 diastereomeric mixture of atropisomers. To simplify the spectra the measurements were undertaken at 80 °C. ^{1}H NMR (400 MHz, C_6D_6, 80 °C): δ 8.73 (d, 1H, *J* = 6.0 Hz), 7.83-7.70 (m, 3H), 7.69 (d, 1H, *J* = 8.4 Hz), 7.61-7.51 (m, 4H), 7.46 (d, 1H, *J* = 7.2 Hz), 7.42-7.38 (m, 3H), 7.29 (t, 1H, *J* = 7.2 Hz), 7.27 (t, 1H, *J* = 8.0 Hz), 7.13-7.01 (m, 4H) ppm. ^{13}C NMR (100 MHz, C_6D_6, 80 °C): δ 161.2, 143.3, 139.8, 139.3, 138.2, 137.0, 134.5, 133.9, 133.8, 133.4, 129.9, 129.1, 128.6, 128.5, 128.4, 128.2, 127.9, 127.6, 127.4, 127.2, 127.1, 127.1, 127.1, 126.5, 126.4, 126.3, 126.2, 125.6, 120.1 ppm. HRMS(EI) calcd. for $C_{29}H_{19}N$ (M^+) 381.1517. Found 381.1506.

1-(4-(Pyren-1-yl)naphthalen-1-yl)isoquinoline (14)

Following the general procedure starting from **10** (300 mg, 0.63 mmol) and after flash chromatography on silica gel (*n*-hexane/EtOAc 5:1), **14** was obtained as a light-brown foam (175 mg, 70% yield). NMR spectra recorded at 25 °C showed a ca. 0.4:0.6 diastereomeric mixture of atropisomers. To simplify the spectra the measurements were undertaken at 80 °C. ^{1}H NMR (400 MHz, C_6D_6, 80 °C): δ 8.75 (d, 1H, *J* = 4.8 Hz), 8.04-7.88 (m, 7H), 7.78-7.66 (m, 6H), 7.59 (br, 2H), 7.41 (d, 1H, *J* = 4.8 Hz), 7.30 (t, 1H, *J* = 6.2 Hz), 7.09-7.05 (m, 3H) ppm. ^{13}C NMR (100 MHz, C_6D_6, 80 °C): δ 161.3, 143.4, 140.3, 138.4, 137.1, 136.6, 132.2, 131.9, 131.7, 130.0, 129.2, 127.6, 127.3, 127.2, 126.5, 126.4, 126.3, 125.8, 125.5, 124.9, 120.2 ppm, some carbons peaks were hidden under the C_6D_6 peak. HRMS(EI) calcd. for $C_{35}H_{21}N$ (M^+) 455.1674. Found 455.1669.

1-(4-(Anthracen-1-yl)naphthalen-1-yl)isoquinoline (15)

Following the general procedure starting from **11** (300 mg, 0.66 mmol) and after flash chromatography on silica gel (*n*-hexane/EtOAc 5:1), **15** was obtained as a light-yellow foam (142 mg, 60% yield). NMR spectra recorded at 25 °C showed a ca. 0.4:0.6 diastereomeric mixture of atropisomers. To simplify the spectra the measurements were undertaken at 80 °C. ^{1}H NMR (500 MHz, C_6D_6, 80 °C): δ 8.73 (d, 1H, *J* = 5.6 Hz), 8.33 (s, 1H), 8.23 (br s, 1H), 7.96 (d, 1H, *J* = 8.0 Hz), 7.81 (d, 1H, *J* = 7.6 Hz), 7.71 (d, 1H, *J* = 8.8 Hz), 7.69 (d, 1H, *J* = 7.4 Hz), 7.65 (d, 1H, *J* = 5.9 Hz), 7.58 (d, 2H, *J* = 7.5 Hz), 7.41-7.38 (m, 3H), 7.30 (t, 2H, *J* = 7.4 Hz), 7.20 (t, 1H, *J* = 7.4 Hz), 7.11-6.98 (m, 5H) ppm. ^{13}C NMR (125 MHz, C_6D_6, 80 °C): δ 160.8, 142.9, 142.8, 139.6, 139.0, 138.0, 136.7, 133.6, 133.2, 132.3, 132.0, 131.9, 129.6, 128.9, 128.7, 128.4, 128.2, 127.3, 127.0, 126.8,

126.8, 126.7, 126.6, 126.1, 126.0, 125.5, 125.0, 124.8, 124.7, 119.8 ppm. HRMS(EI) calcd. for $C_{33}H_{21}N$ (M^+) 431.1674. Found 431.1681.

2. ^{1}H and ^{13}C NMR spectra

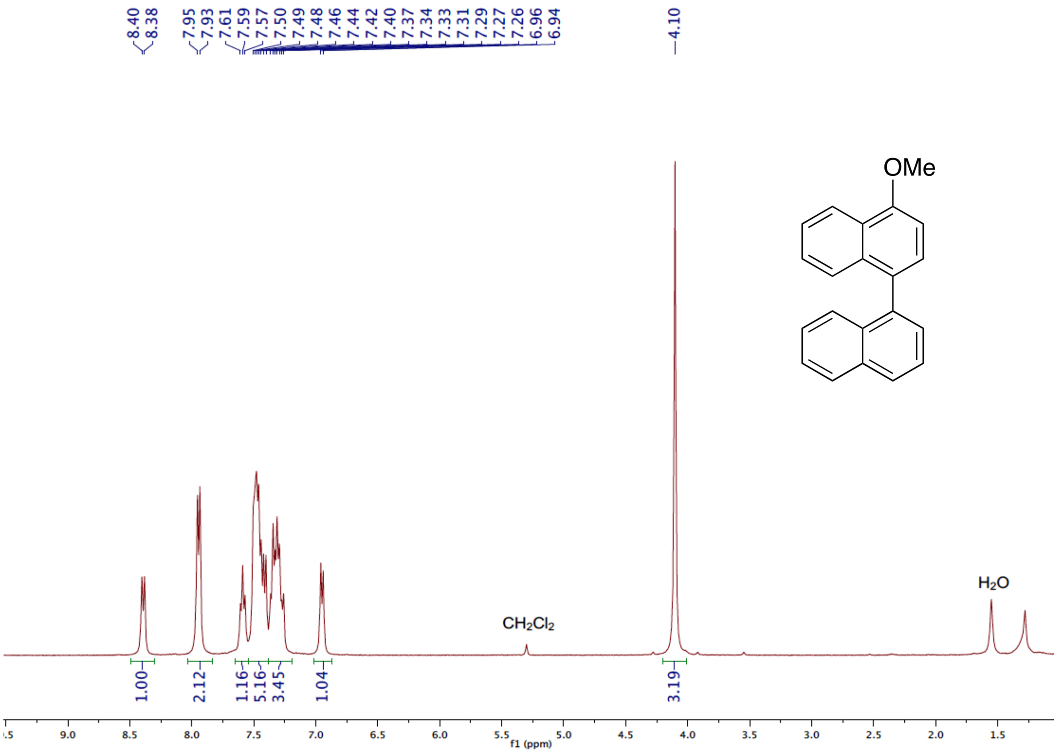

Figure S1. ^{1}H NMR spectrum ($CDCl_3$, 400 MHz, 25 °C) of **2**.

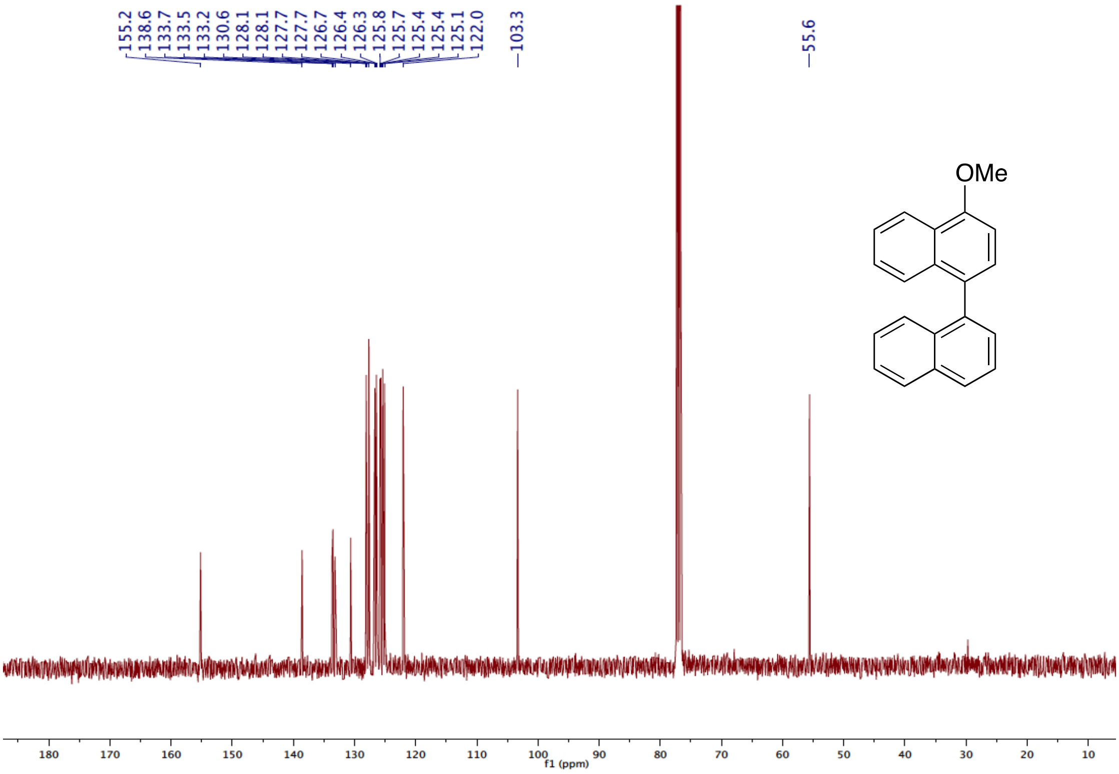

Figure S2. ^{13}C NMR spectrum ($CDCl_3$, 100 MHz, 25 °C) of **2**.

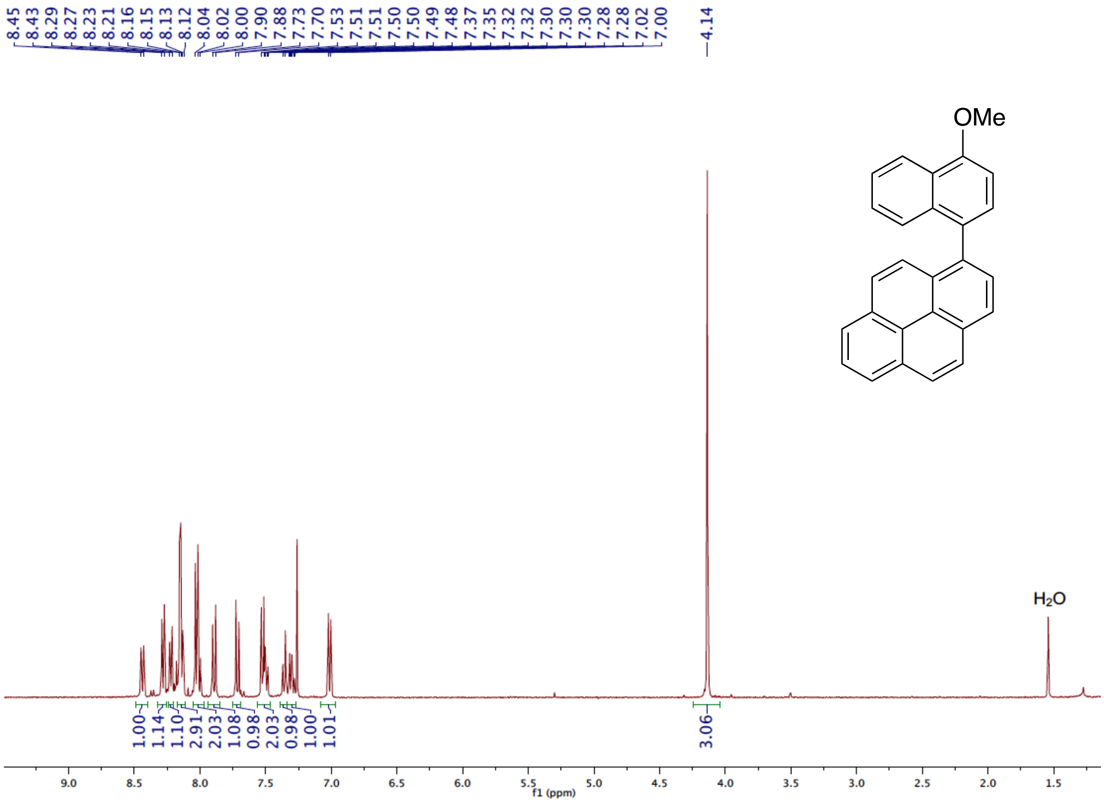

Figure S3. ^{1}H NMR spectrum ($CDCl_3$, 400 MHz, 25 °C) of **3**.

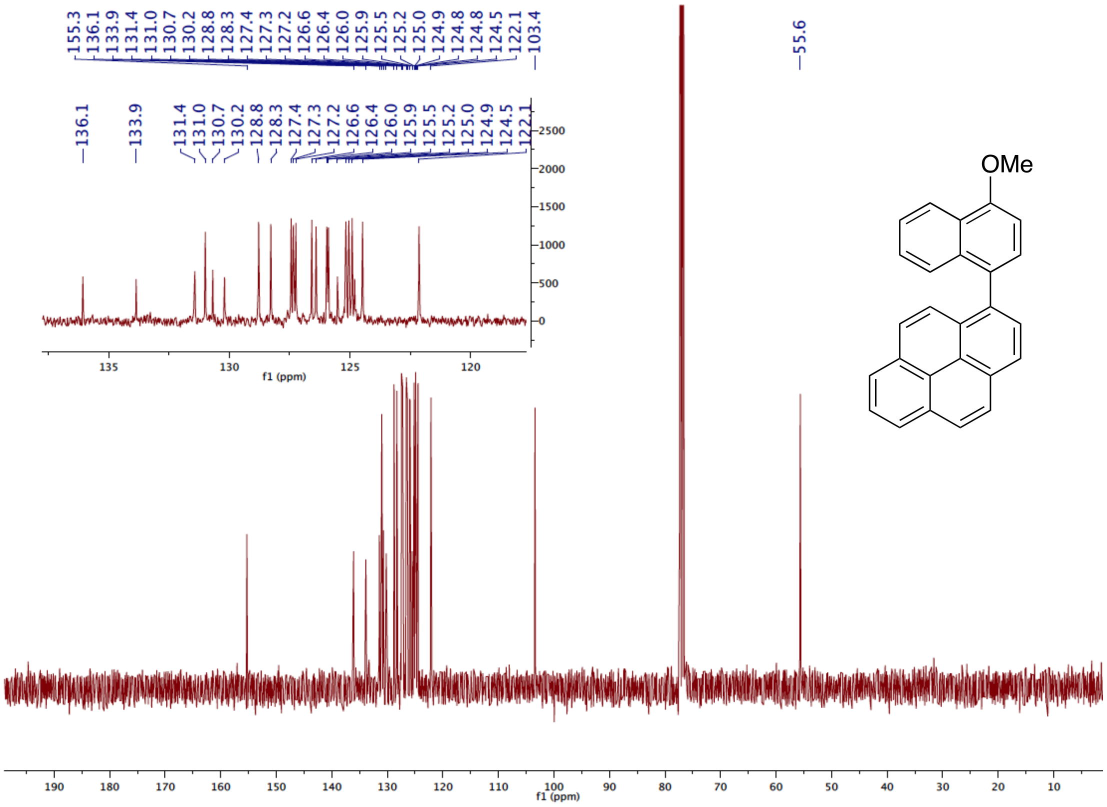

Figure S4. ^{13}C NMR spectrum ($CDCl_3$, 100 MHz, 25 °C) of **3**.

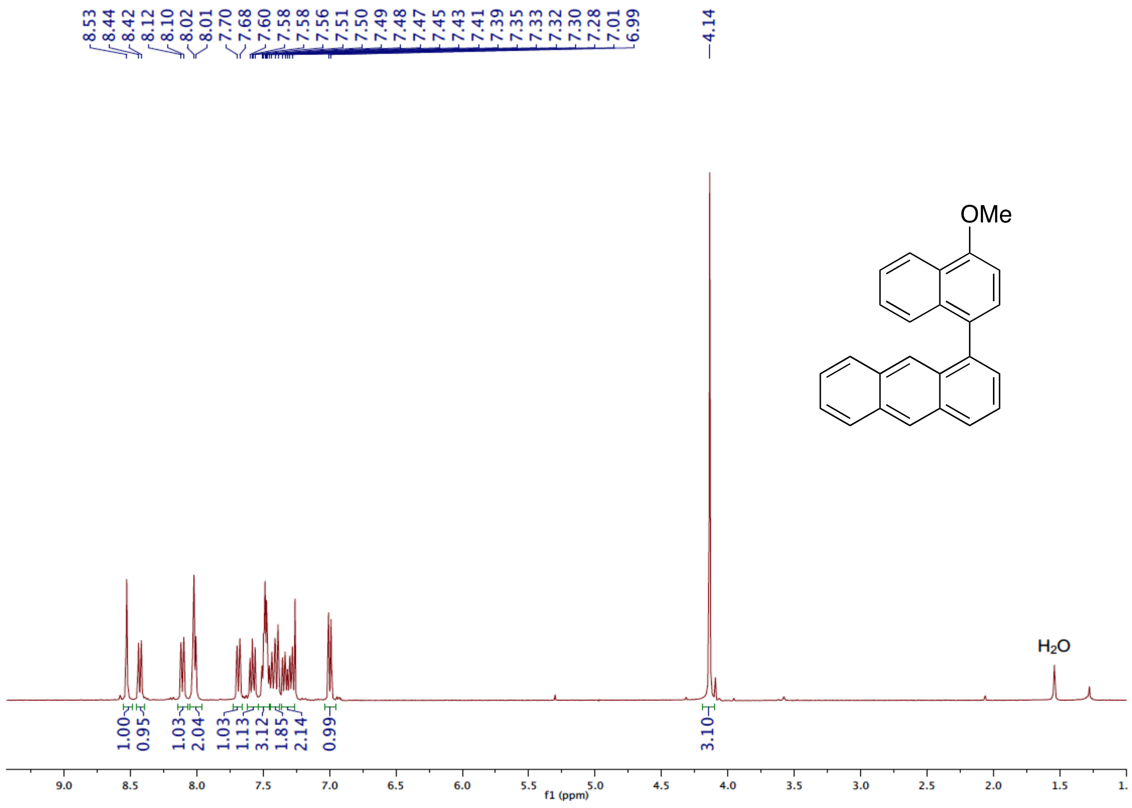

Figure S5. ^{1}H NMR spectrum ($CDCl_3$, 400 MHz, 25 °C) of **5**.

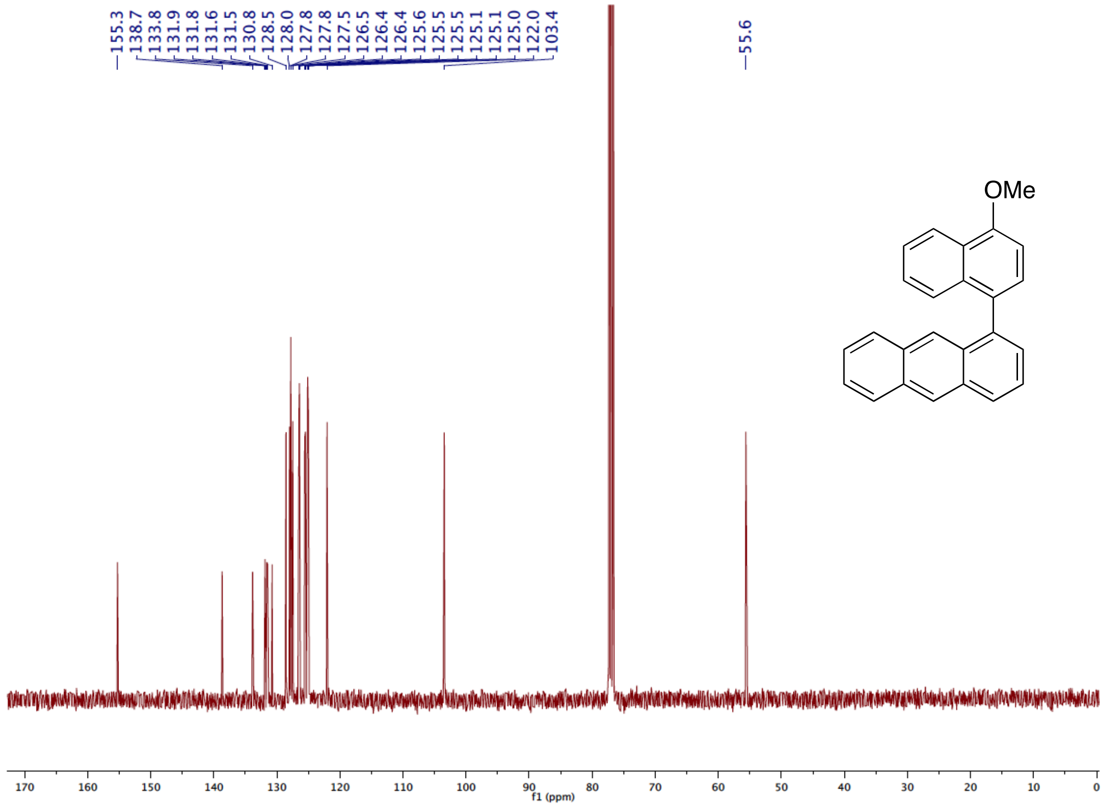

Figure S6. ^{13}C NMR spectrum ($CDCl_3$, 100 MHz, 25 °C) of **5**.

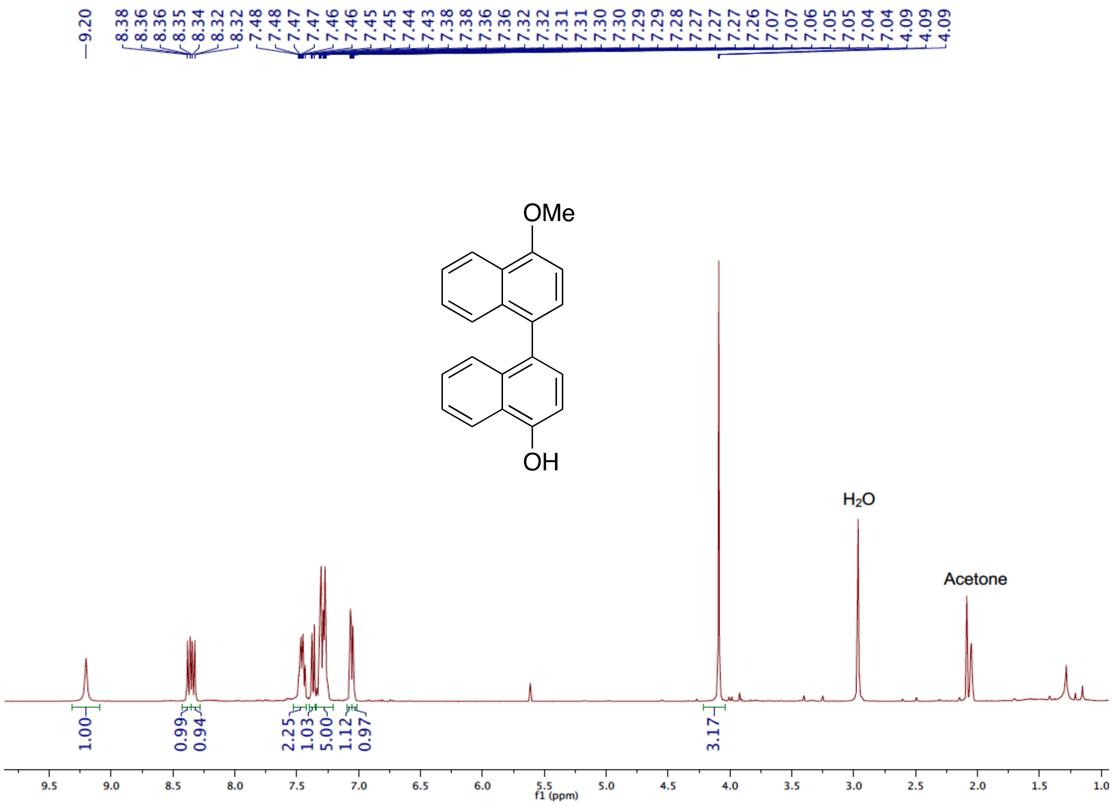

Figure S7. ^{1}H NMR spectrum (acetone-d6, 400 MHz, 25 °C) of **7**.

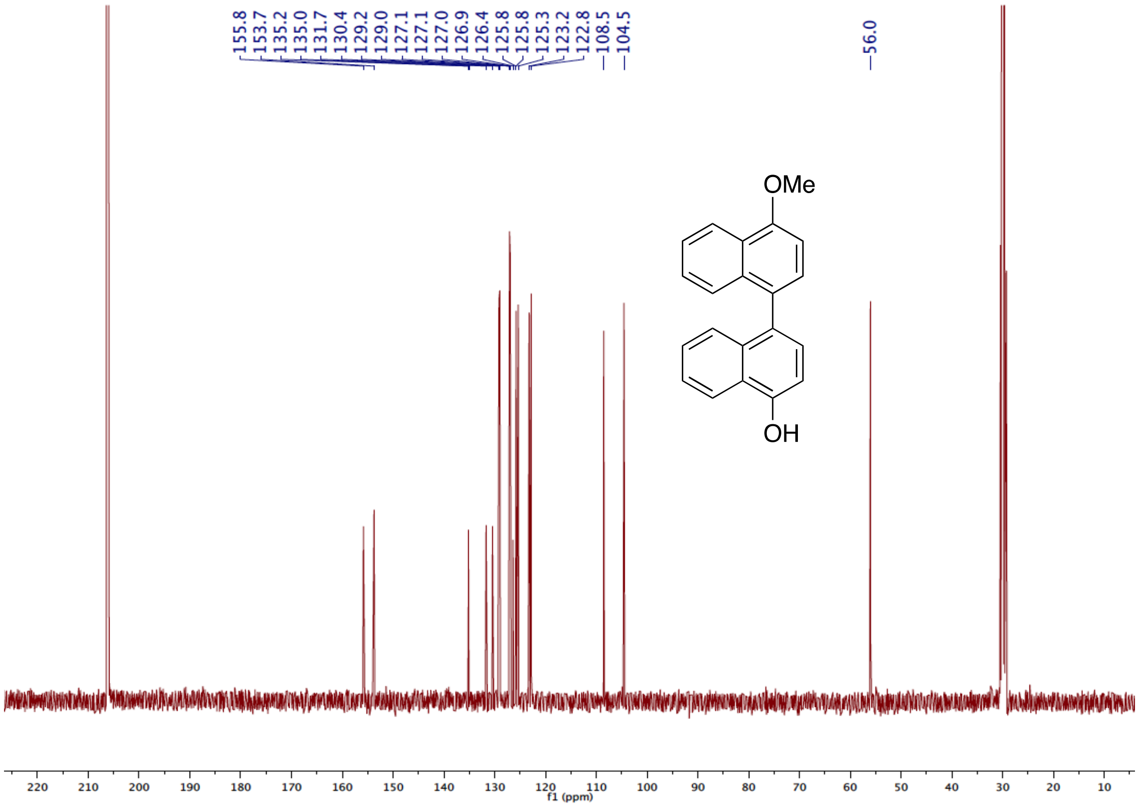

Figure S8. ^{13}C NMR spectrum (acetone-d6, 400 MHz, 25 °C) of **7**.

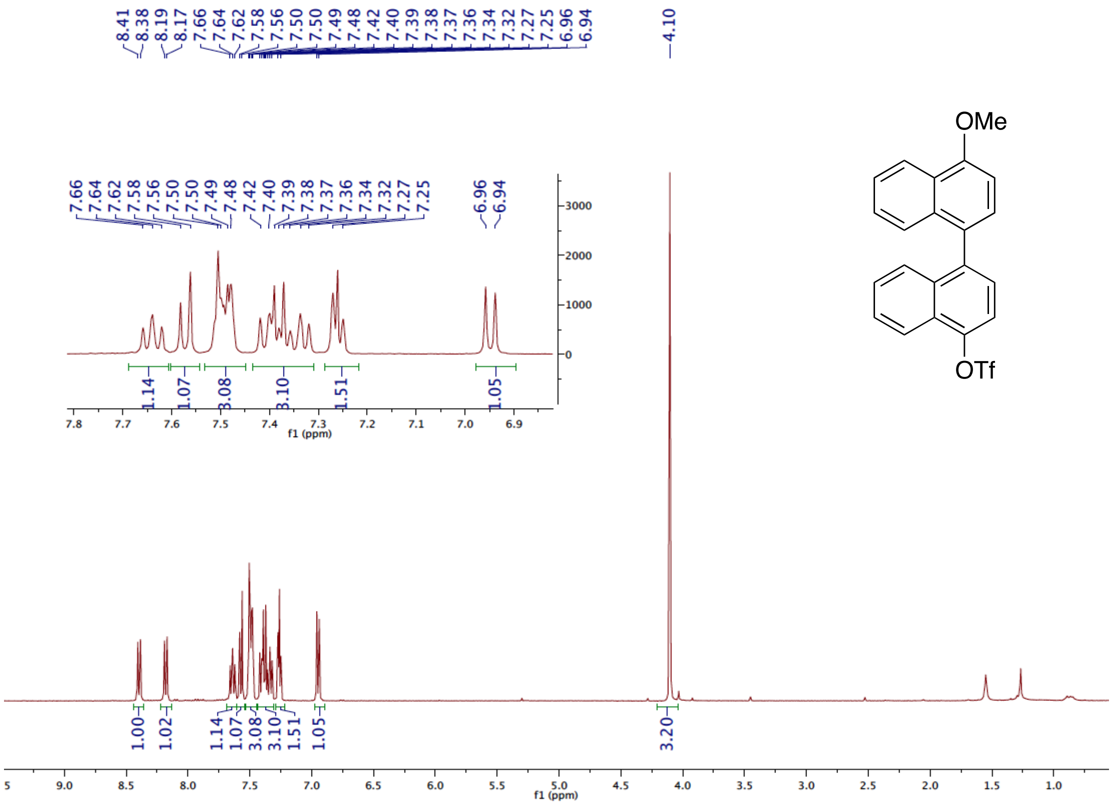

Figure S9. ^{1}H NMR spectrum ($CDCl_3$, 400 MHz, 25 °C) of **8**.

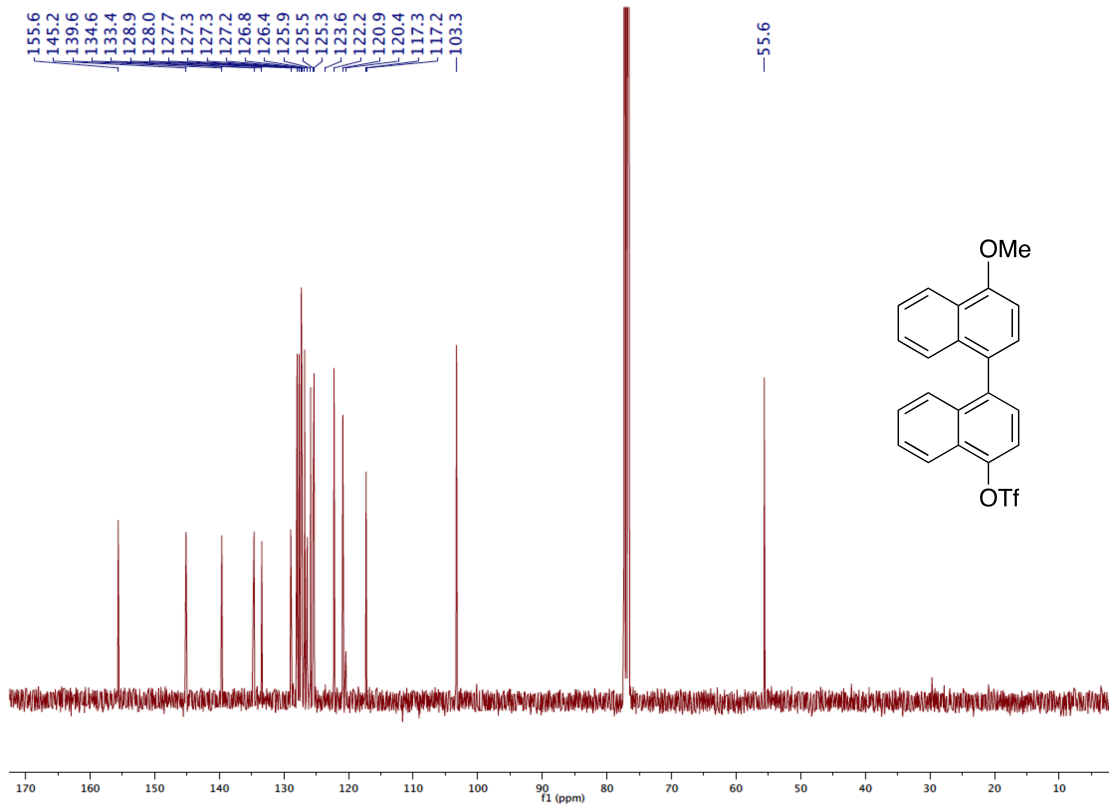

Figure S10. ^{13}C NMR spectrum ($CDCl_3$, 100 MHz, 25 °C) of **8**.

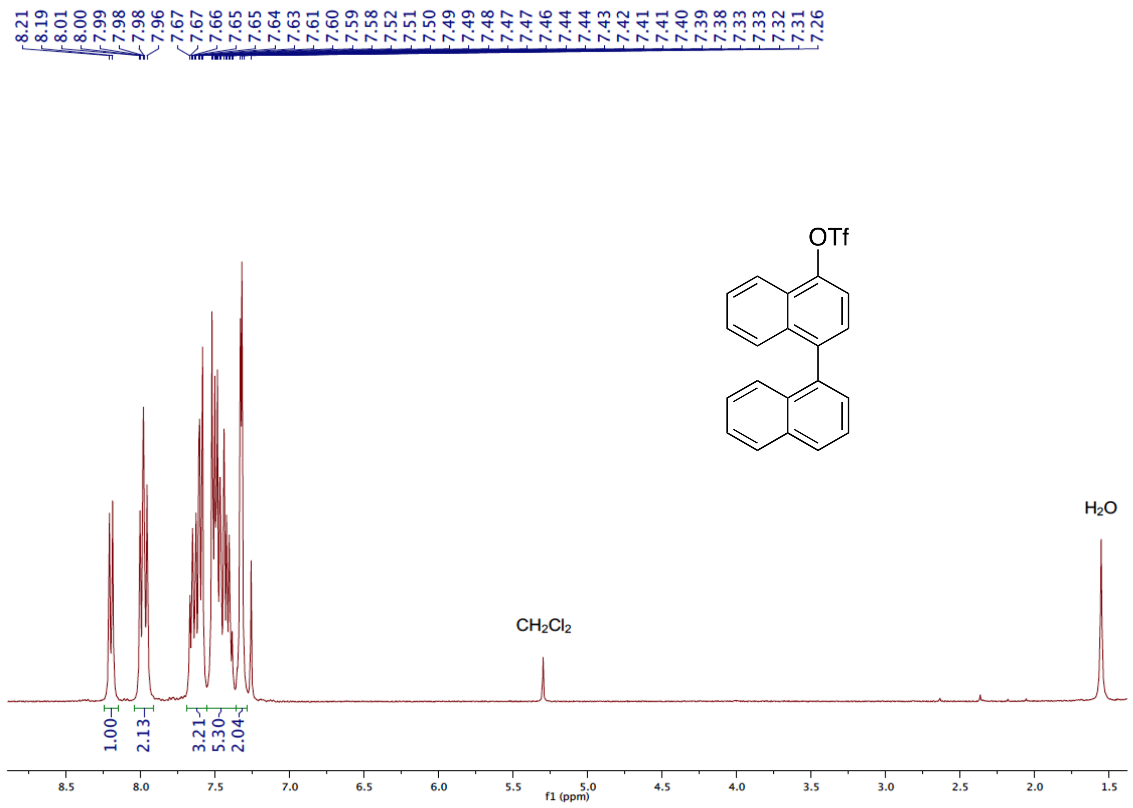

Figure S11. ^{1}H NMR spectrum ($CDCl_3$, 400 MHz, 25 °C) of **9**.

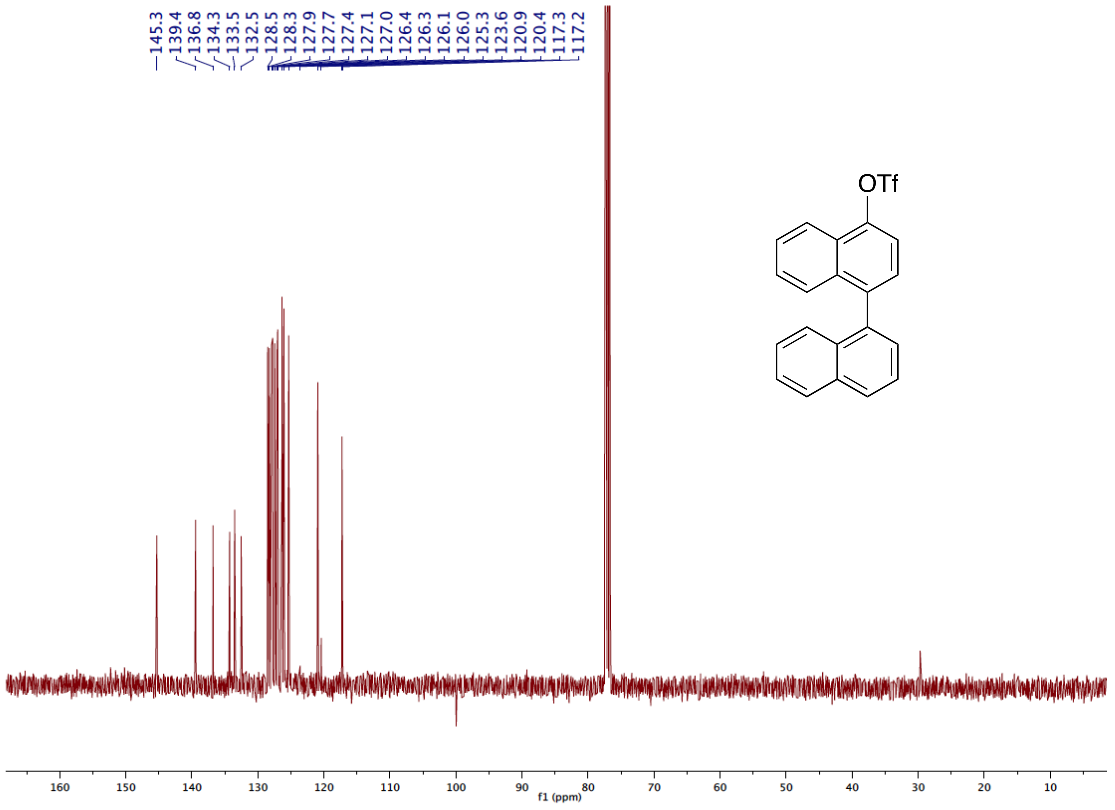

Figure S12. ^{13}C NMR spectrum ($CDCl_3$, 100 MHz, 25 °C) of **9**.

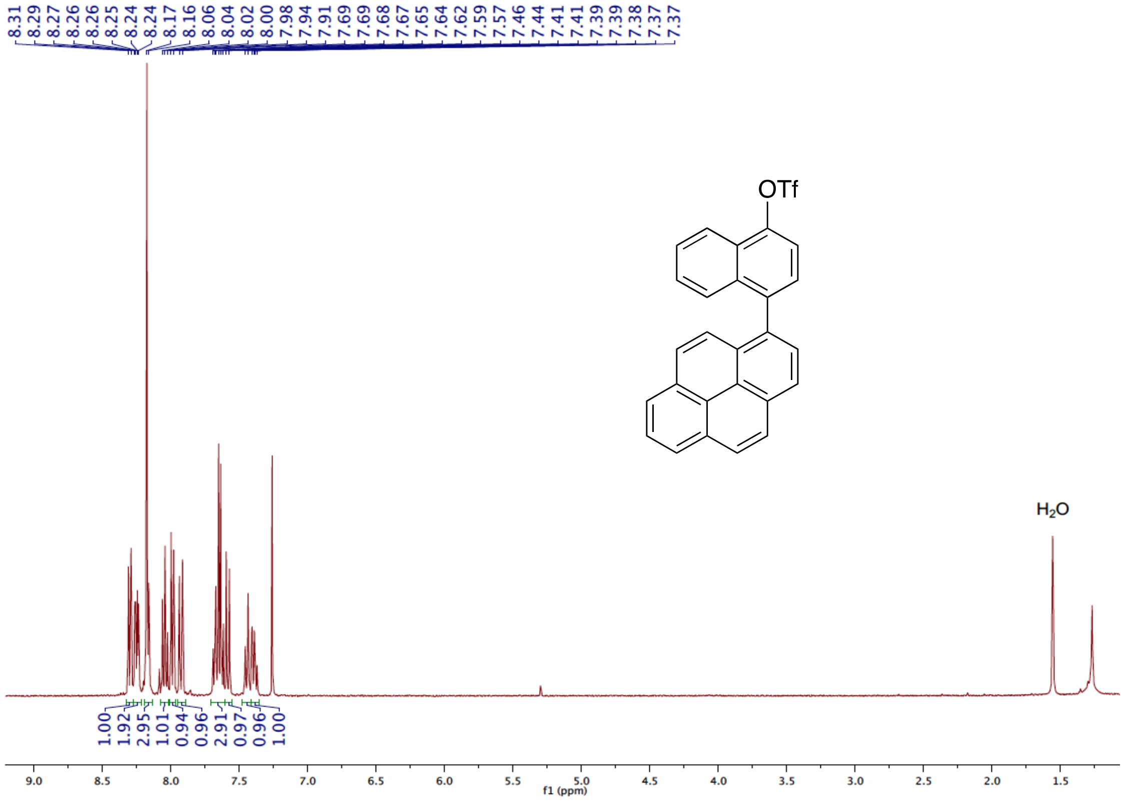

Figure S13. ^{1}H NMR spectrum ($CDCl_3$, 400 MHz, 25 °C) of **10**.

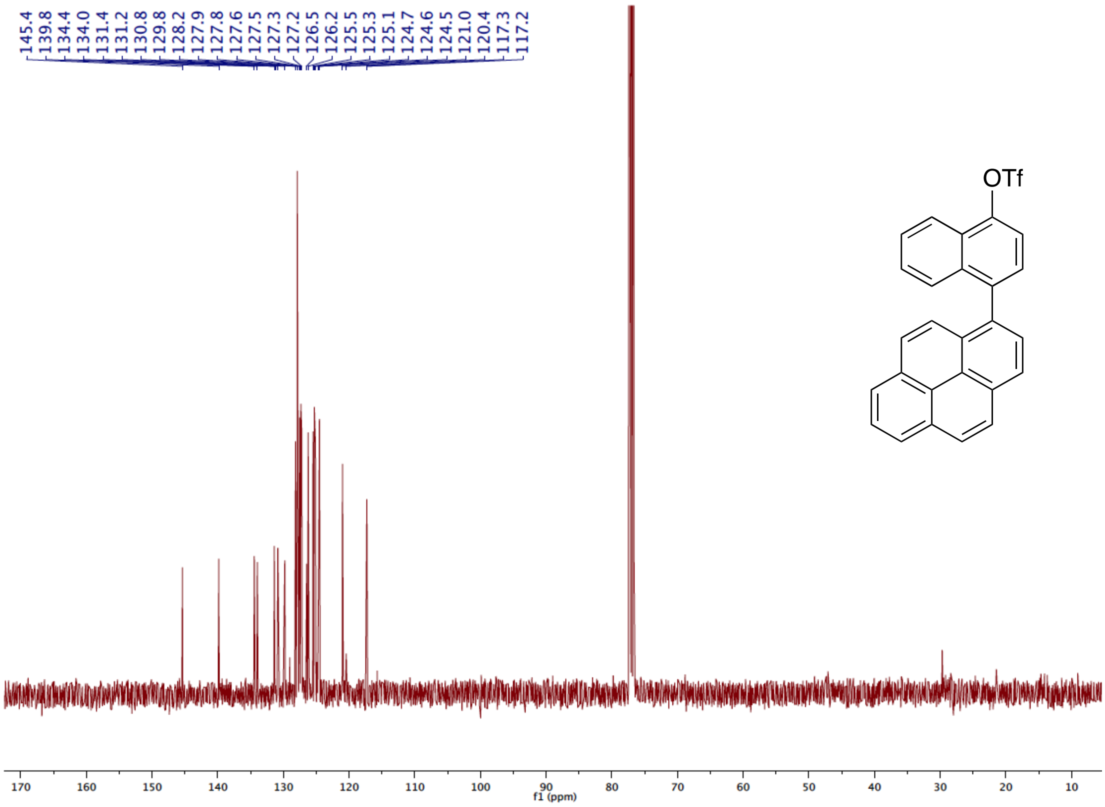

Figure S14. ^{13}C NMR spectrum ($CDCl_3$, 100 MHz, 25 °C) of **10**.

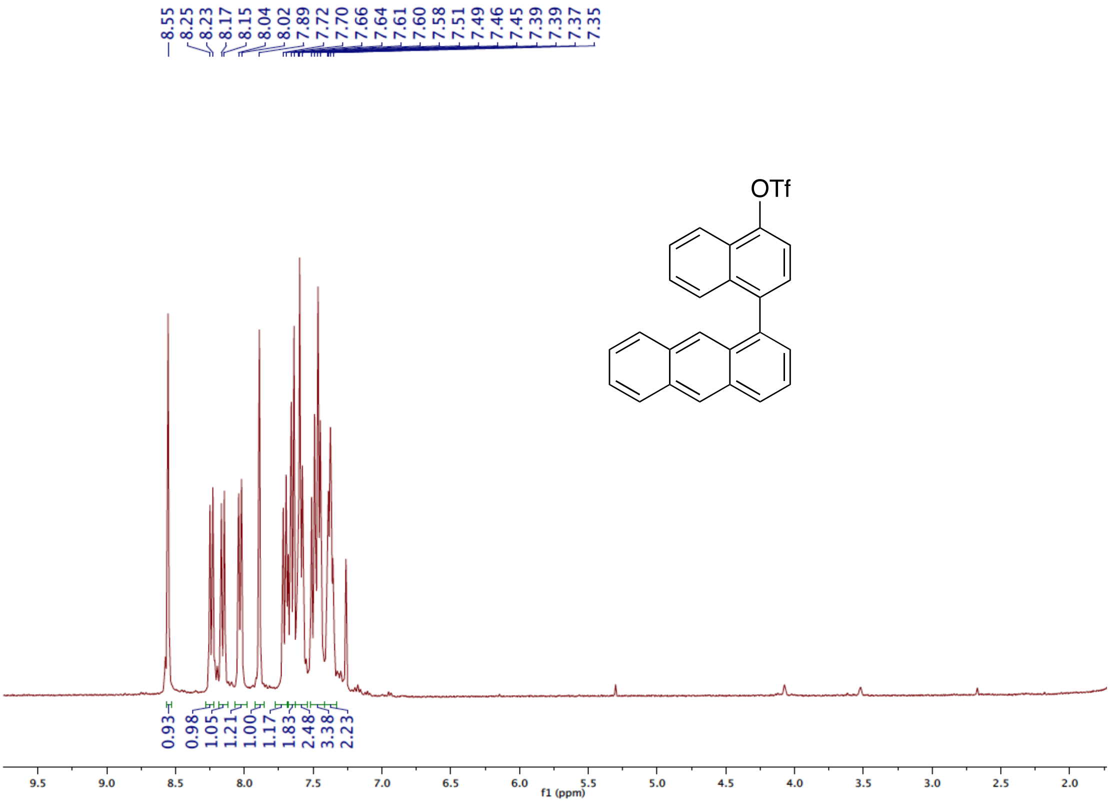

Figure S15. ^{1}H NMR spectrum ($CDCl_3$, 400 MHz, 25 °C) of **11**.

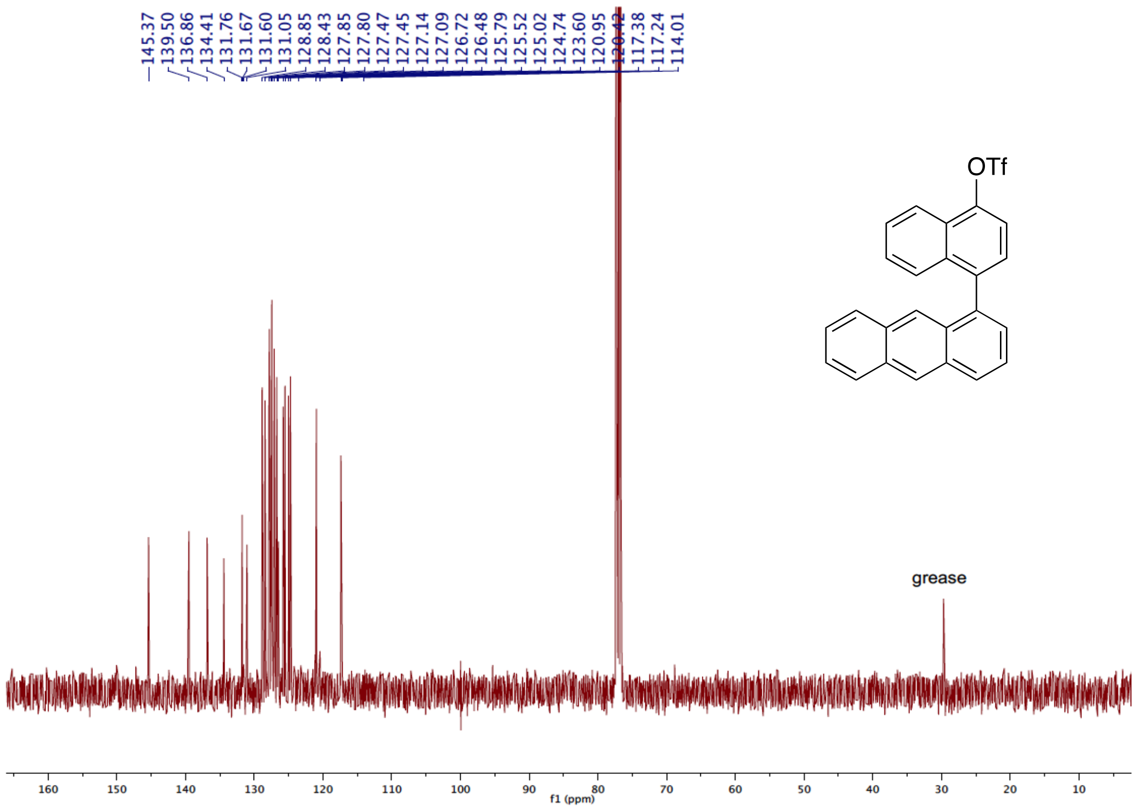

Figure S16. ^{13}C NMR spectrum ($CDCl_3$, 100 MHz, 25 °C) of **11**.

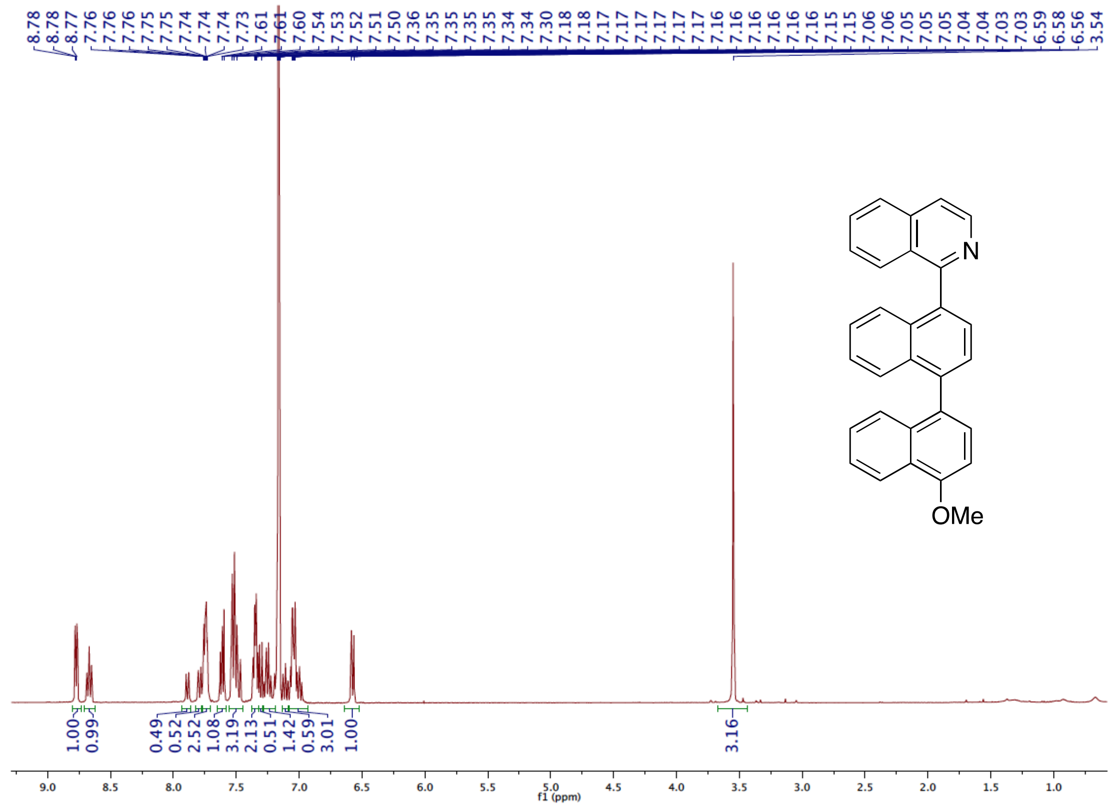

Figure S17. ^{1}H NMR spectrum (C_6D_6, 400 MHz, 25 °C) of **12**.

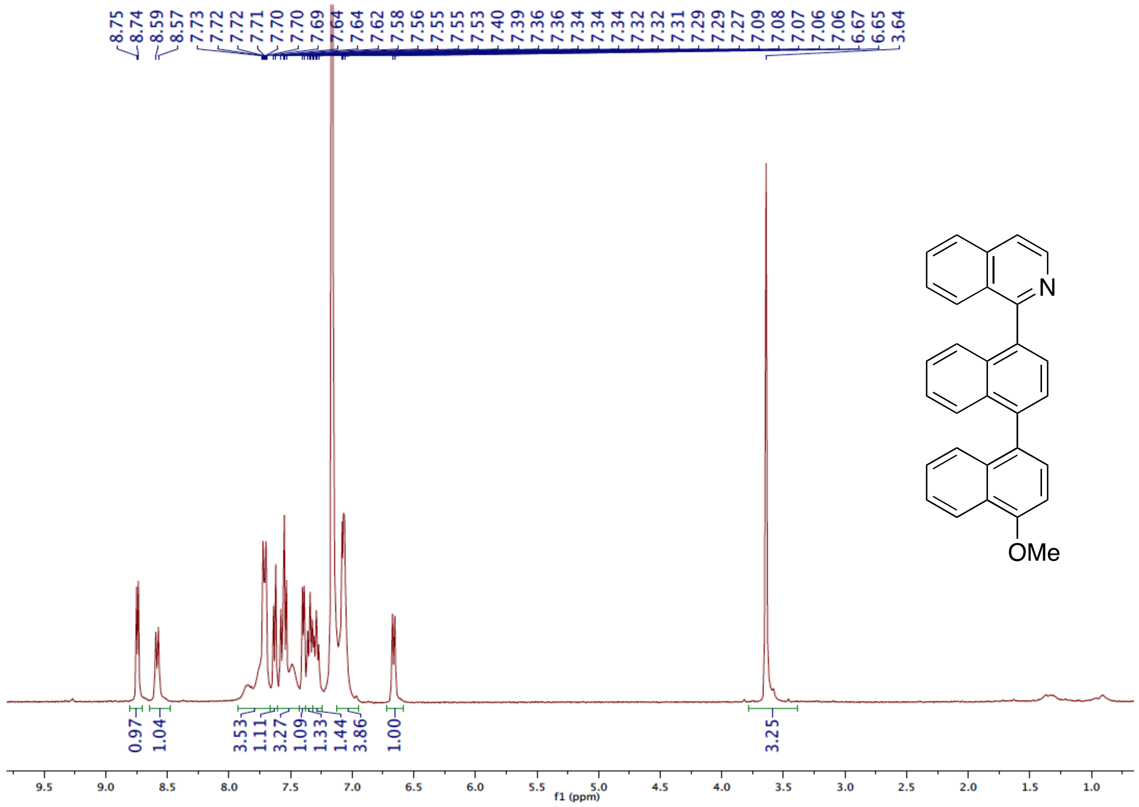

Figure S18. ^{1}H NMR spectrum (C_6D_6, 400 MHz, 80 °C) of **12**.

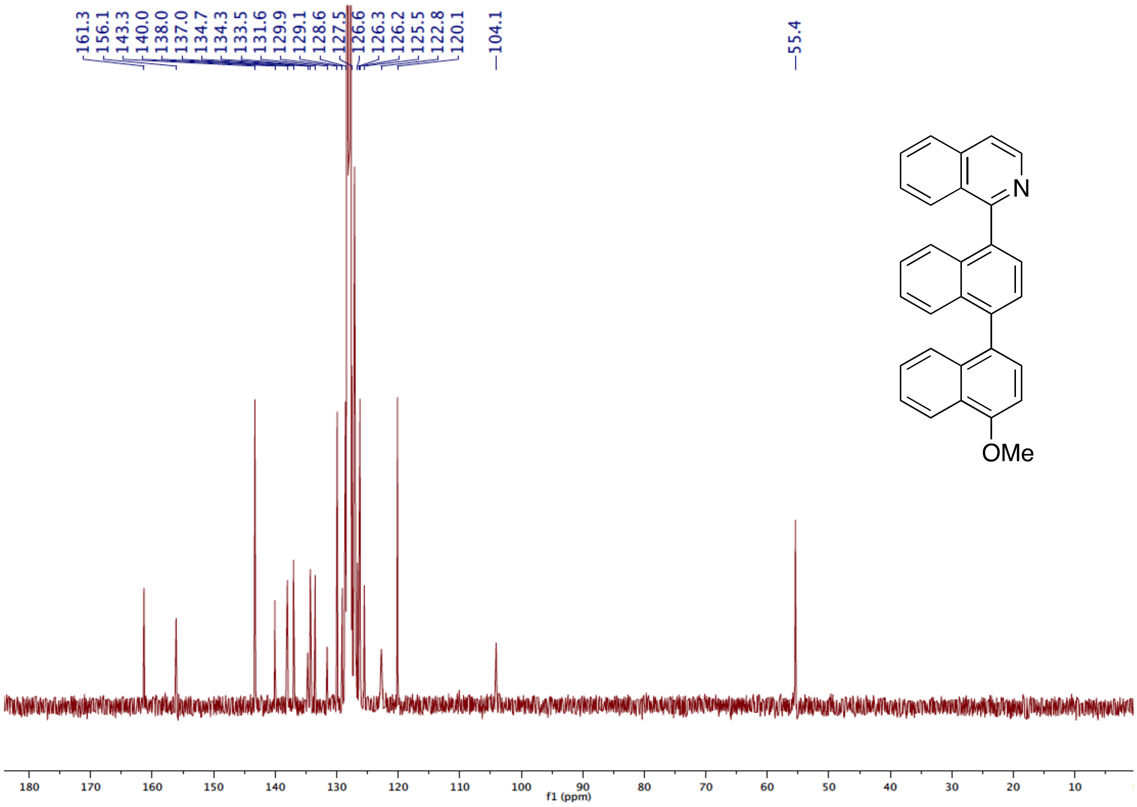

Figure S19. ^{13}C NMR spectrum (C_6D_6, 100 MHz, 80 °C) of **12**.

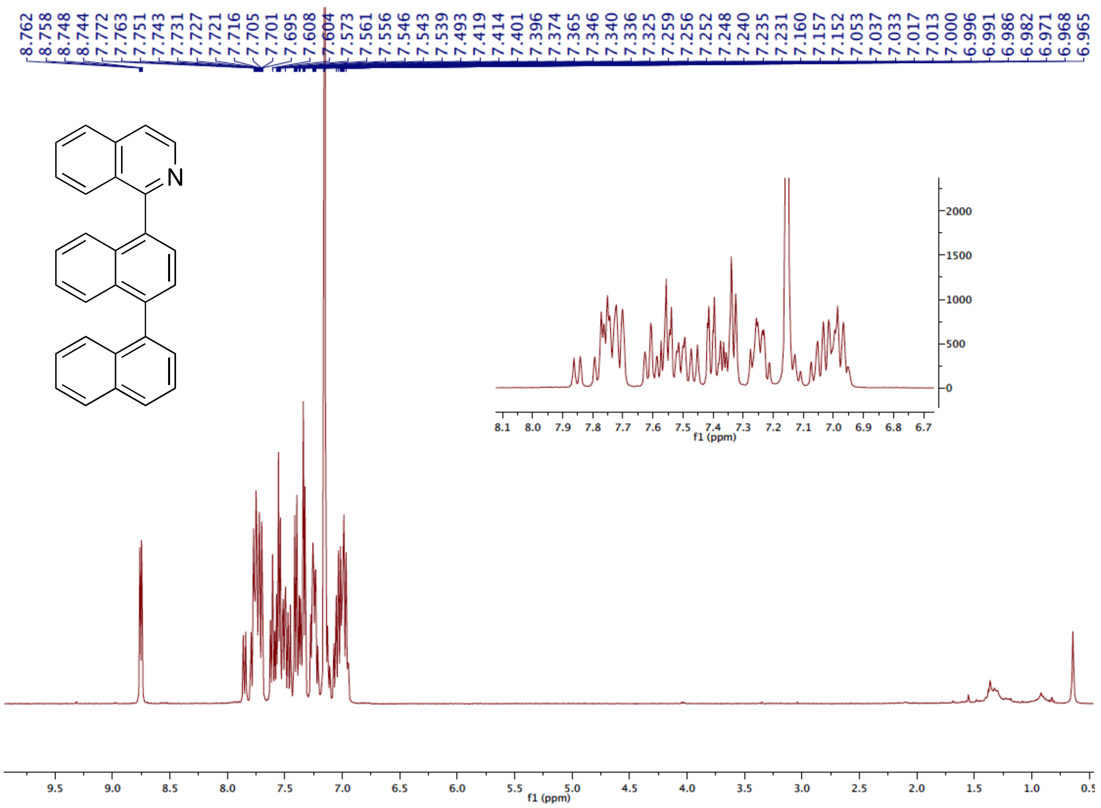

Figure S20. ^{1}H NMR spectrum (C_6D_6, 400 MHz, 25 °C) of **13**.

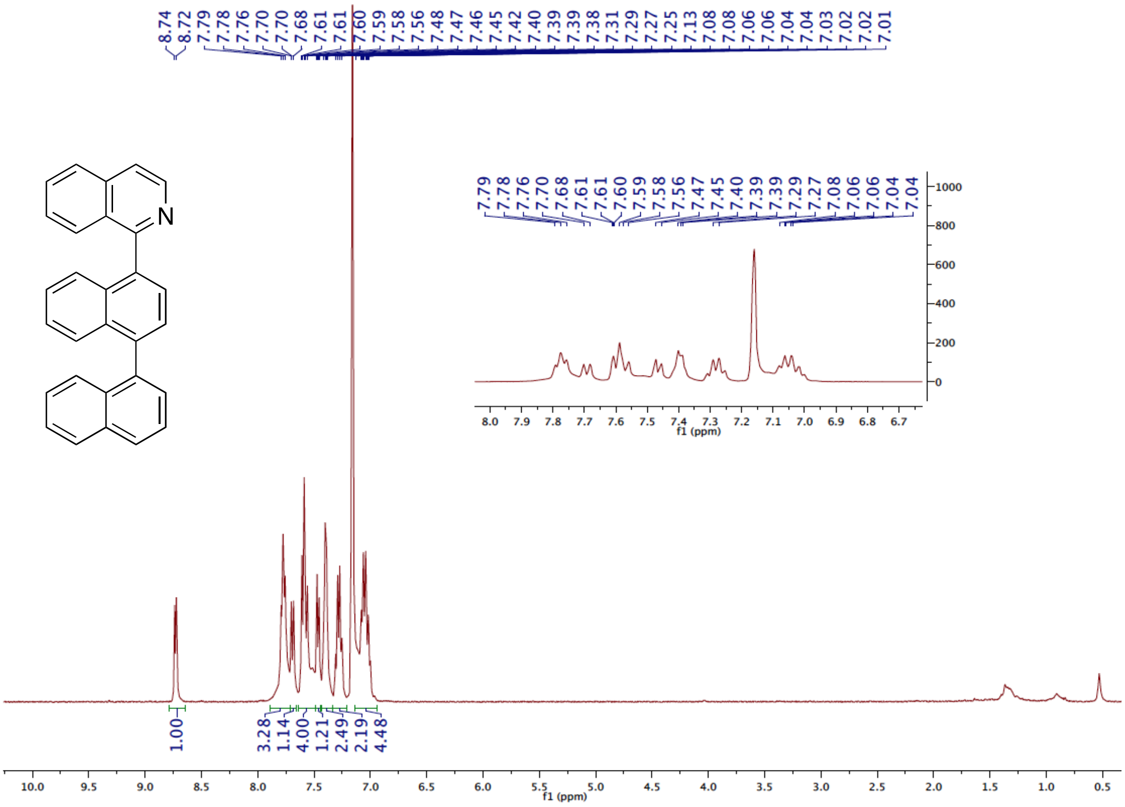

Figure S21. ^{1}H NMR spectrum (C_6D_6, 400 MHz, 80 °C) of **13**.

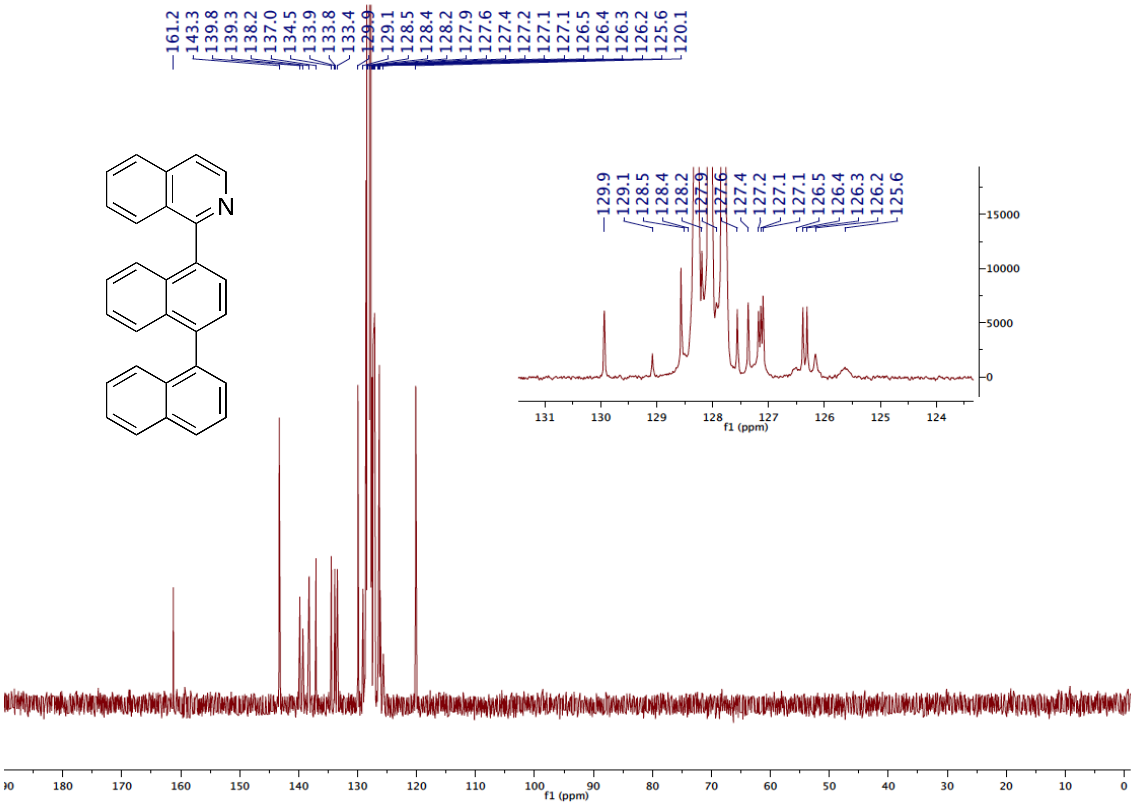

Figure S22. ^{13}C NMR spectrum (C_6D_6, 100 MHz, 80 °C) of **13**.

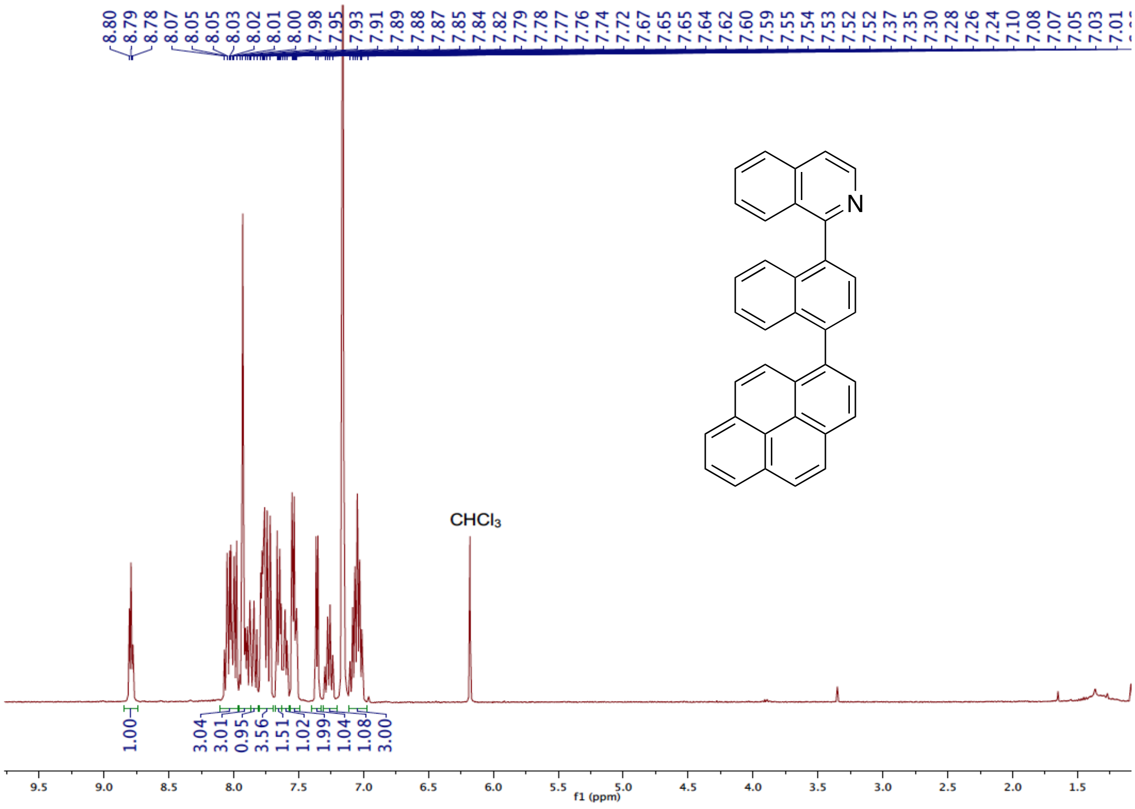

Figure S23. ^{1}H NMR spectrum (C_6D_6, 400 MHz, 25 °C) of **14**.

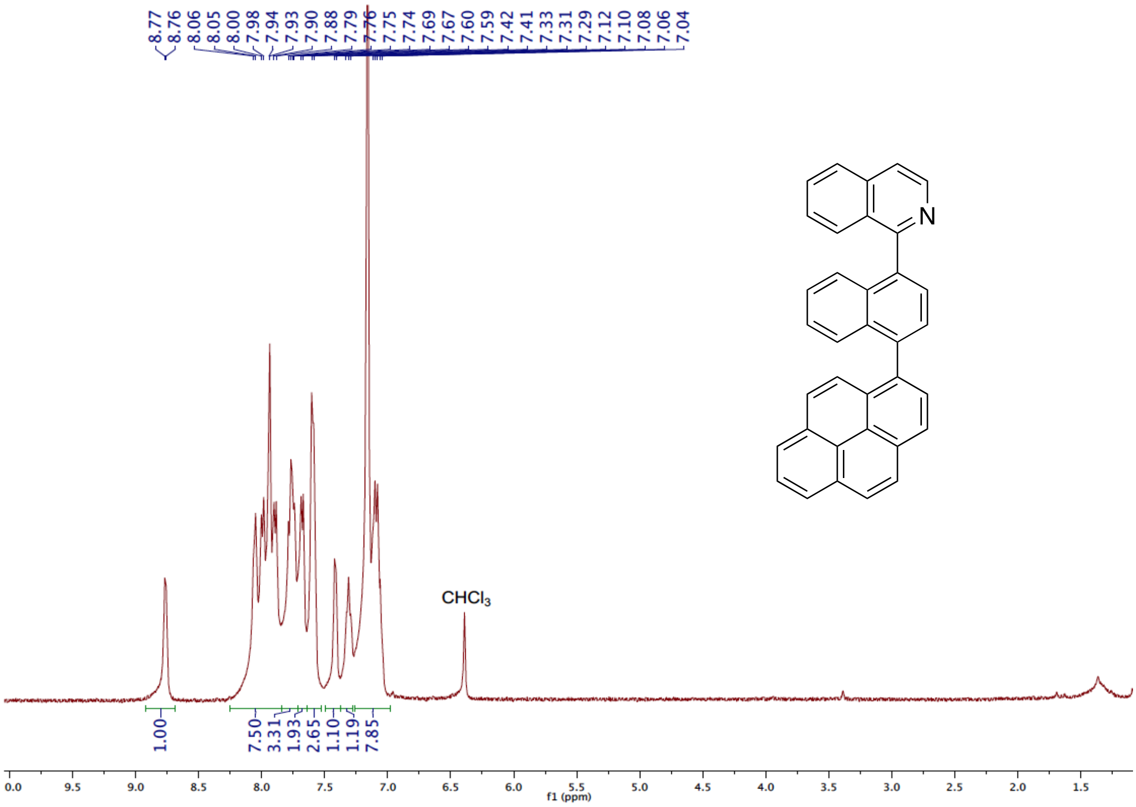

Figure S24. ^{1}H NMR spectrum (C_6D_6, 500 MHz, 80 °C) of **14**.

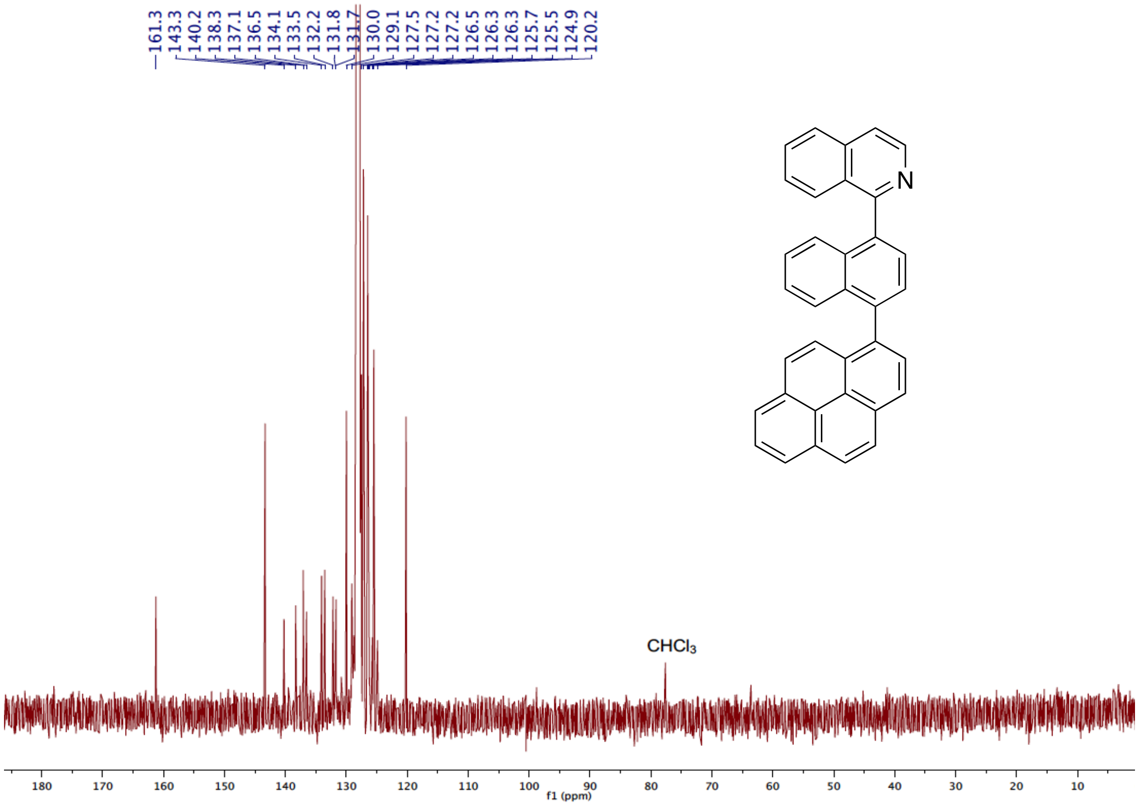

Figure S25. ^{13}C NMR spectrum (C_6D_6, 100 MHz, 80 °C) of **14**.

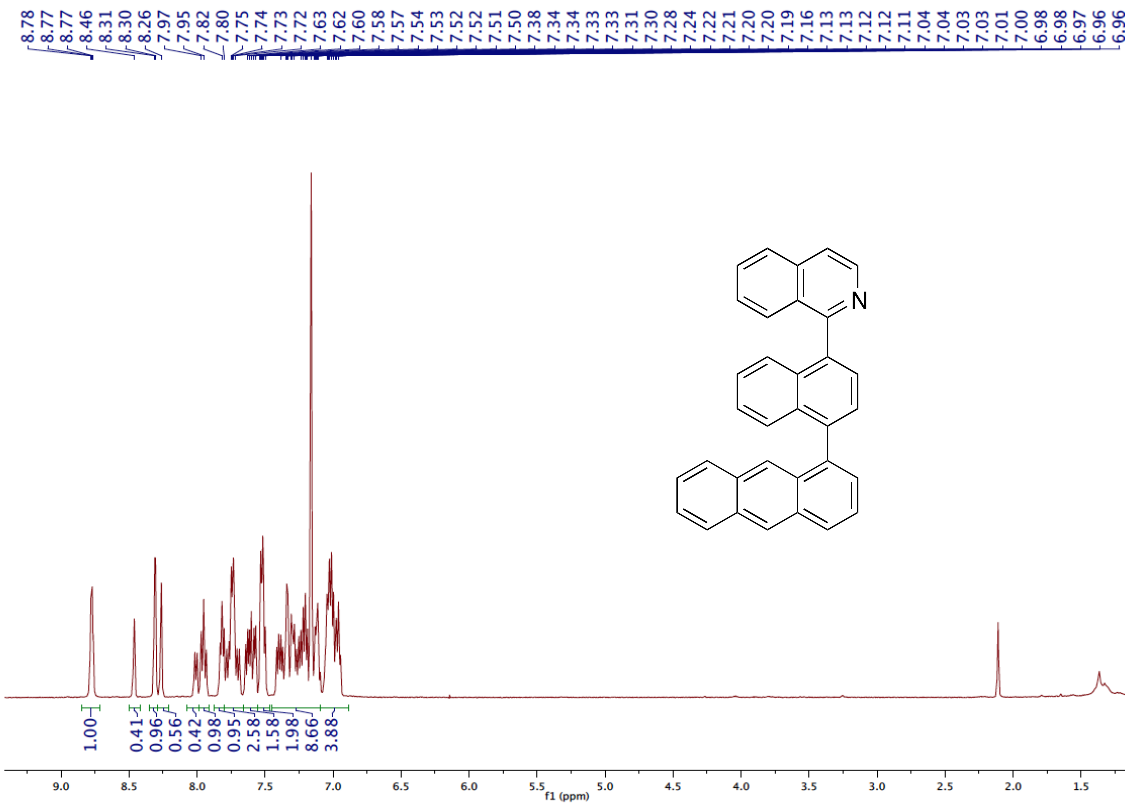

Figure S26. ^{1}H NMR spectrum (C_6D_6, 500 MHz, 25 °C) of **15**.

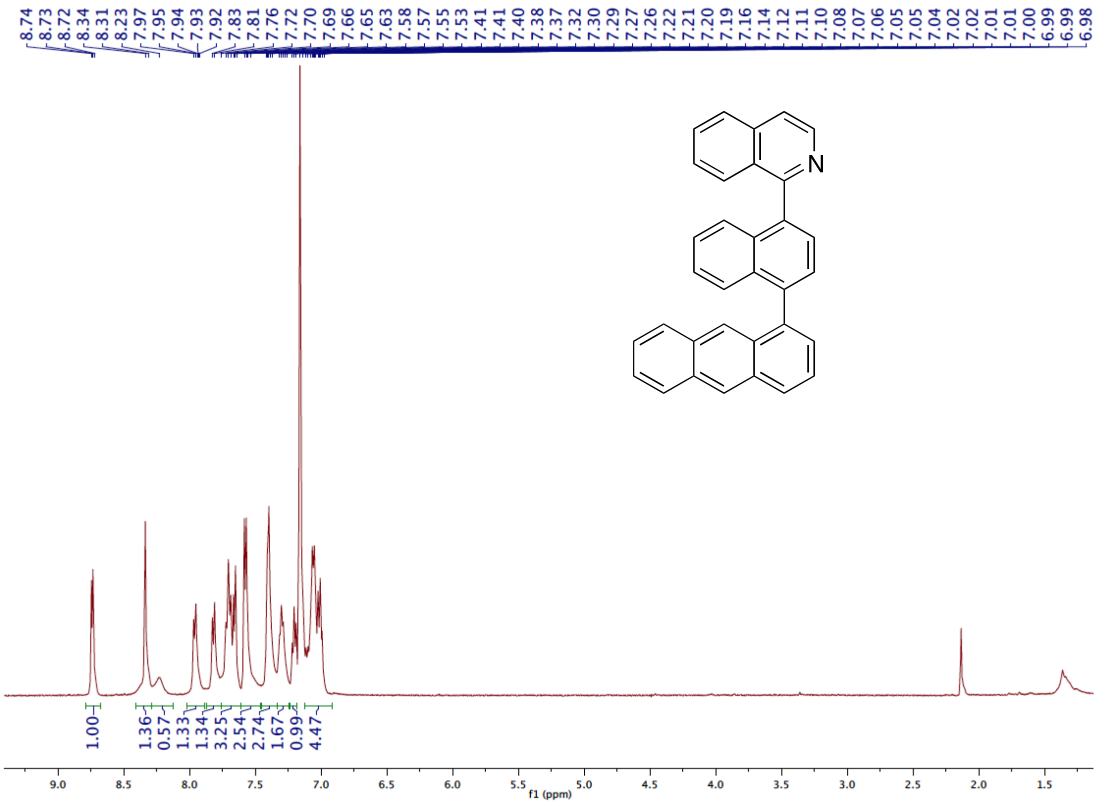

Figure S27. ^{1}H NMR spectrum (C_6D_6, 500 MHz, 80 °C) of **15**.

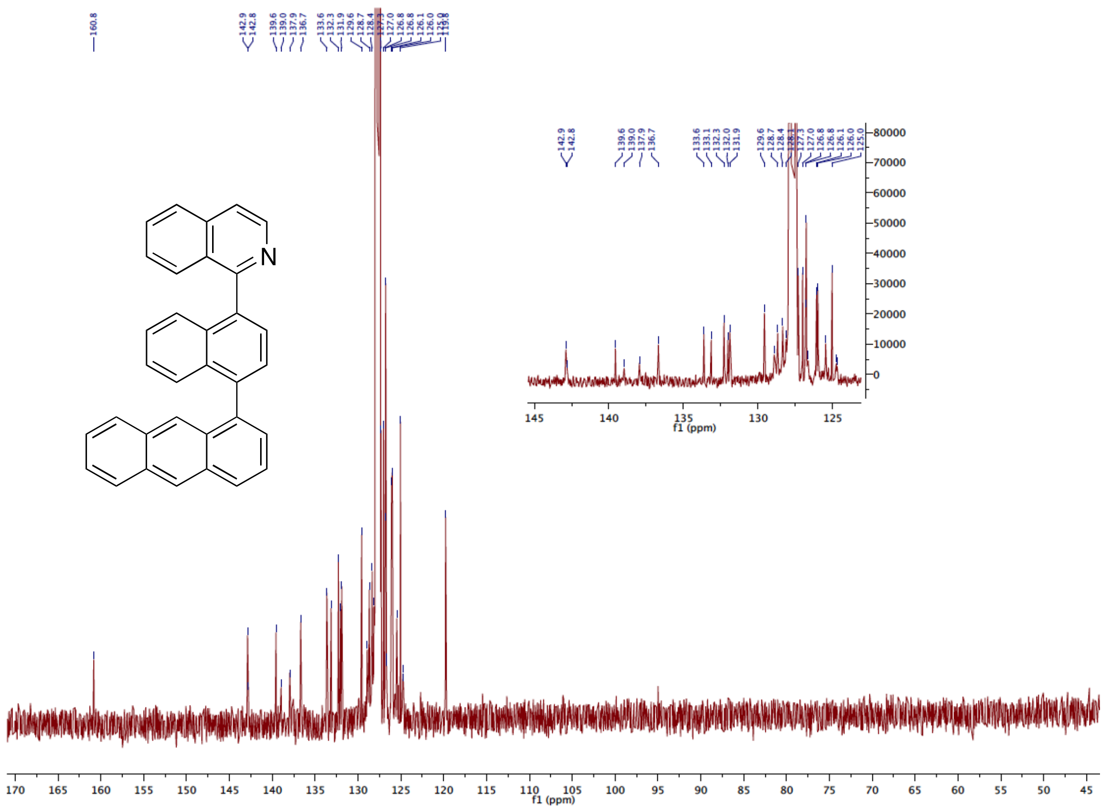

Figure S28. ^{13}C NMR spectrum (C_6D_6, 125 MHz, 80 °C) of **15**.

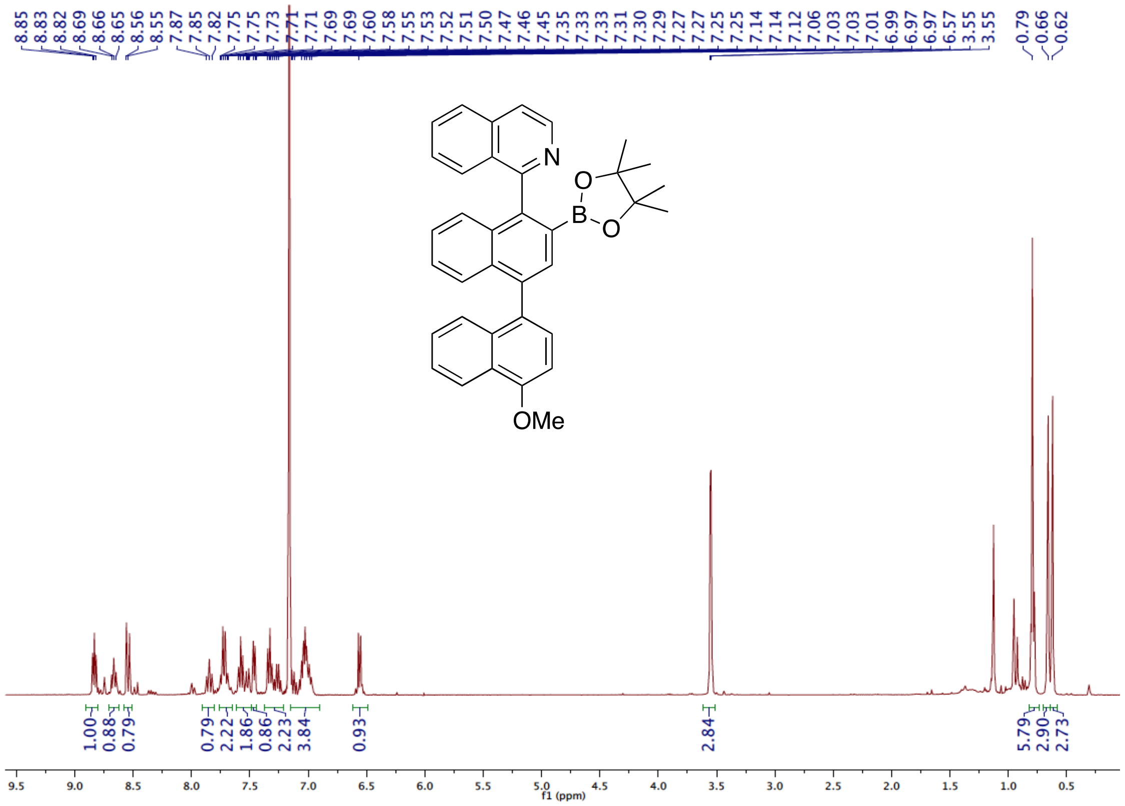

Figure S29. ^{1}H NMR spectrum (C_6D_6, 400 MHz, 25 °C) of **16**.

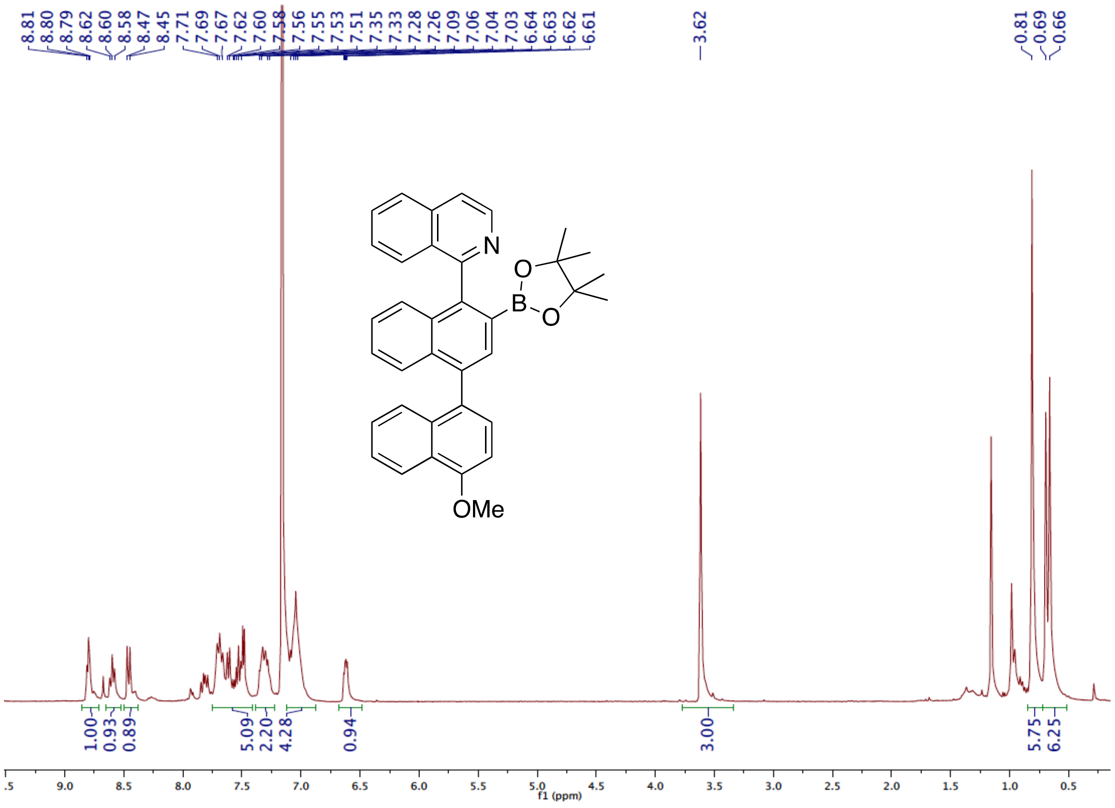

Figure S30. ^{1}H NMR spectrum (C_6D_6, 400 MHz, 80 °C) of **16**.

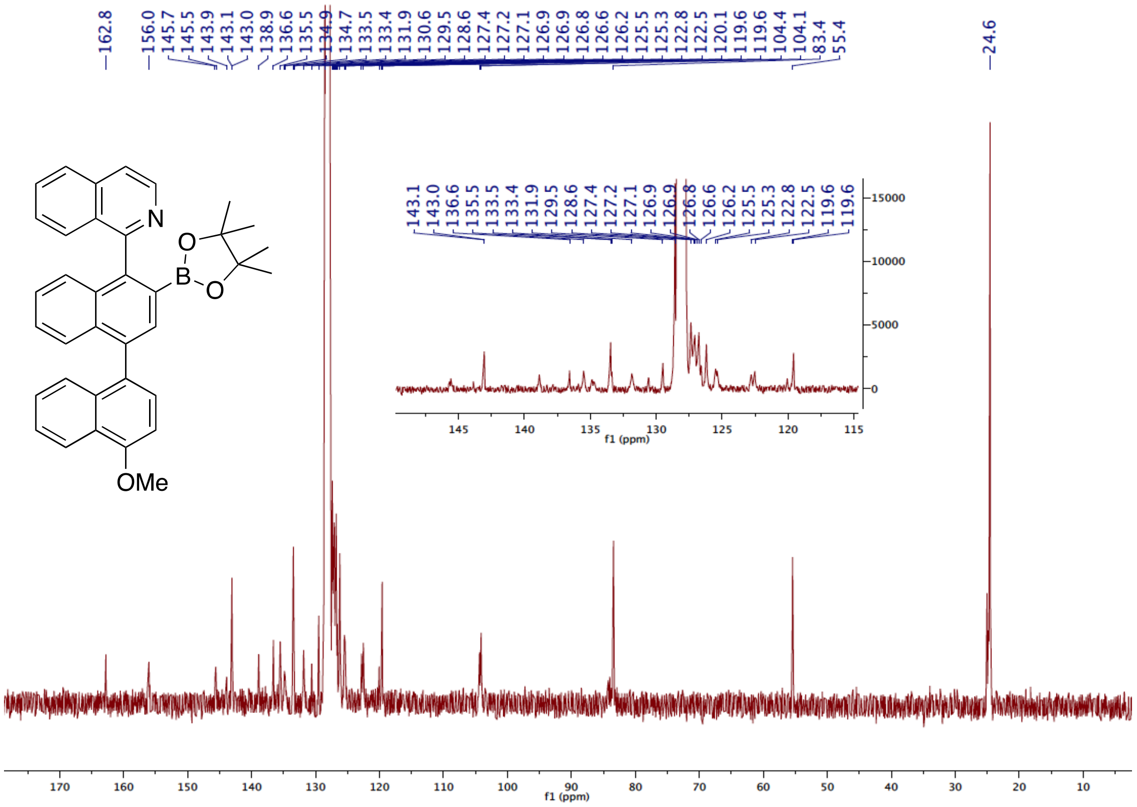

Figure S31. ^{13}C NMR spectrum (C_6D_6, 100 MHz, 80 °C) of **16**.

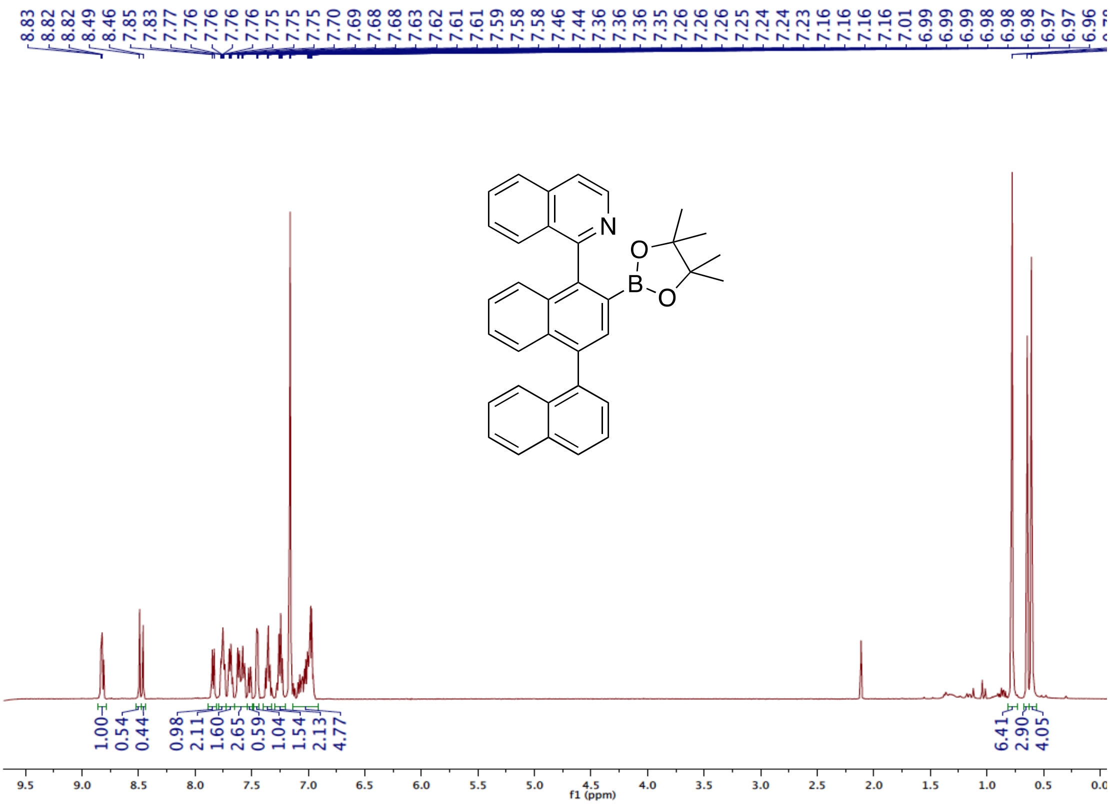

Figure S32. ^{1}H NMR spectrum (C_6D_6, 500 MHz, 25 °C) of **17**.

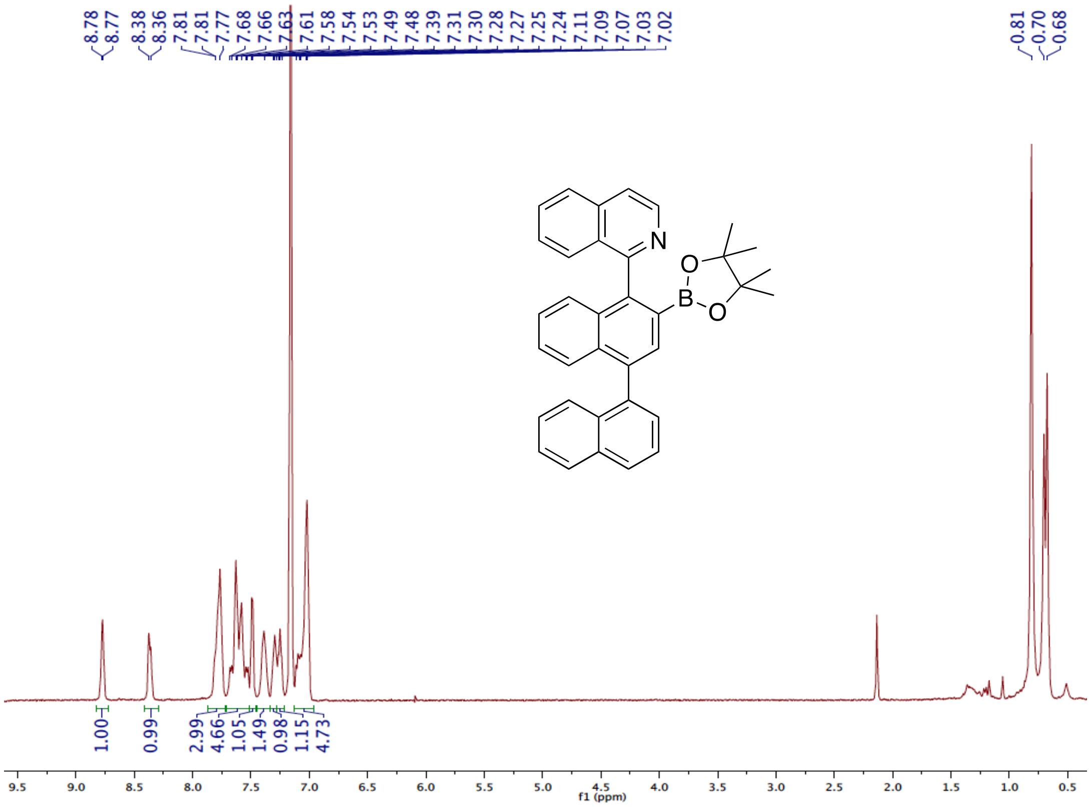

Figure S33. ^{1}H NMR spectrum (C_6D_6, 500 MHz, 80 °C) of **17**.

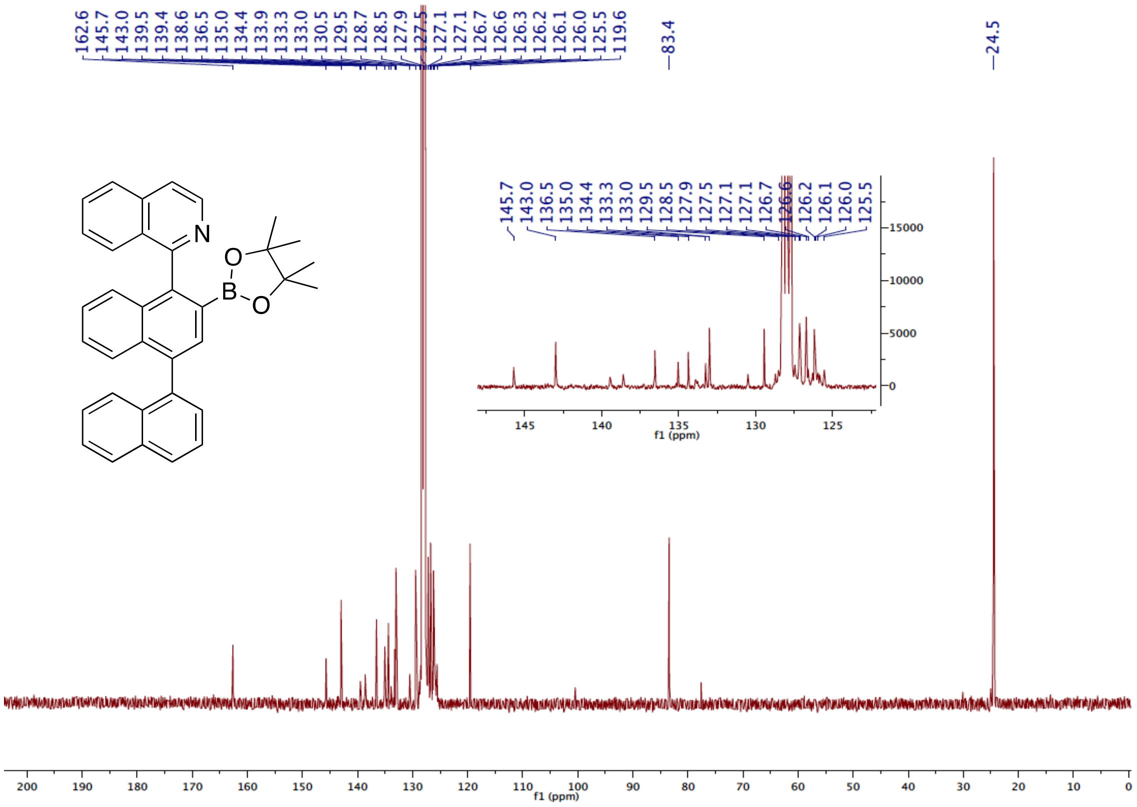

Figure S34. ^{13}C NMR spectrum (C_6D_6, 100 MHz, 80 °C) of **17**.

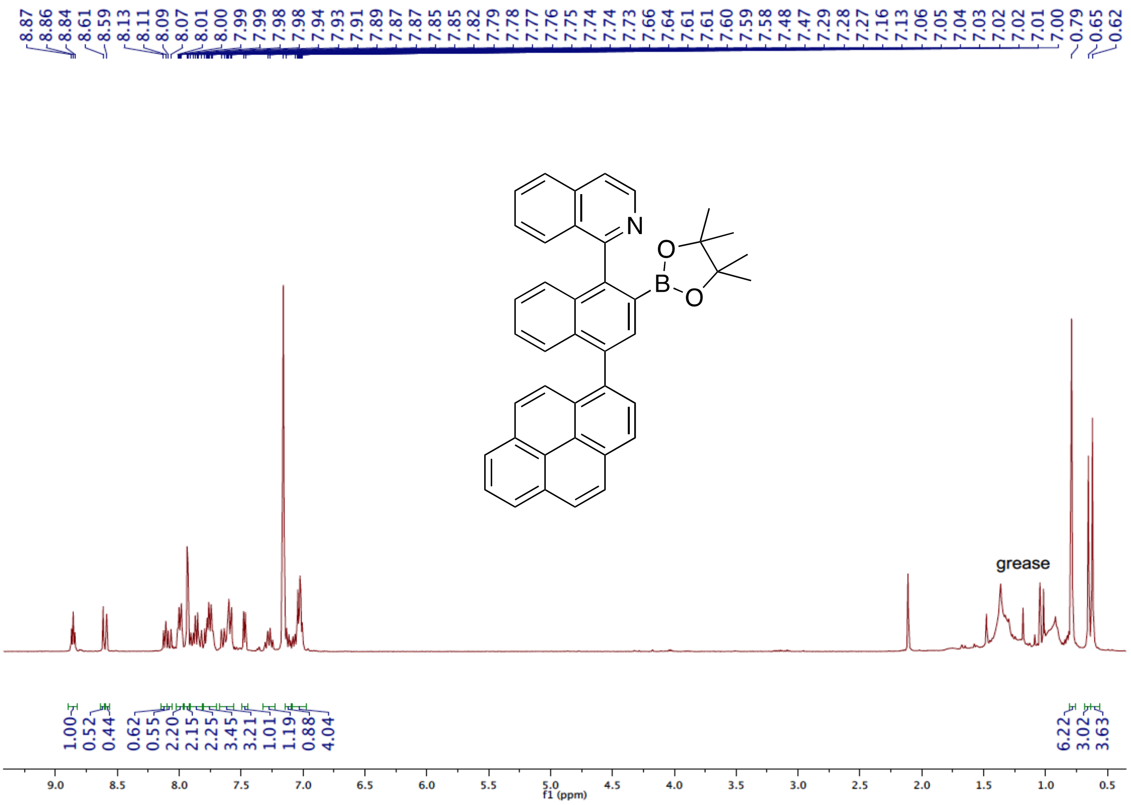

Figure S35. ^{1}H NMR spectrum (C_6D_6, 500 MHz, 25 °C) of **18**.

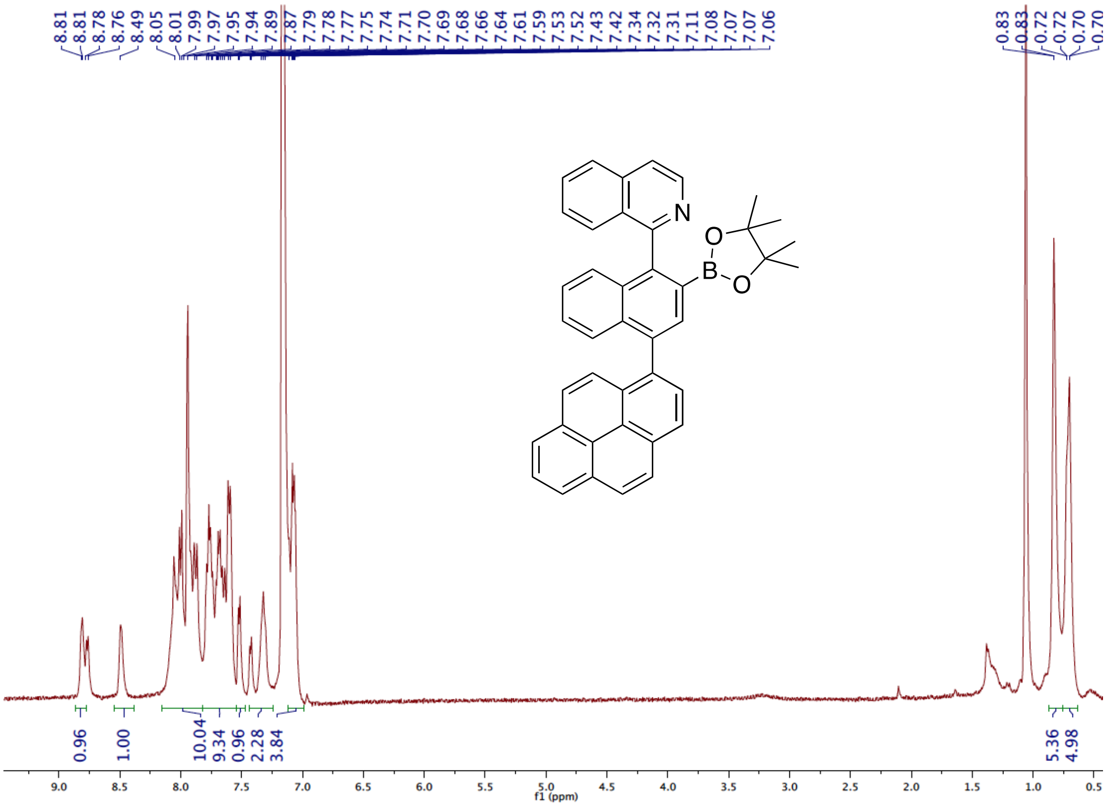

Figure S36. ^{1}H NMR spectrum (C_6D_6, 500 MHz, 80 °C) of **18**.

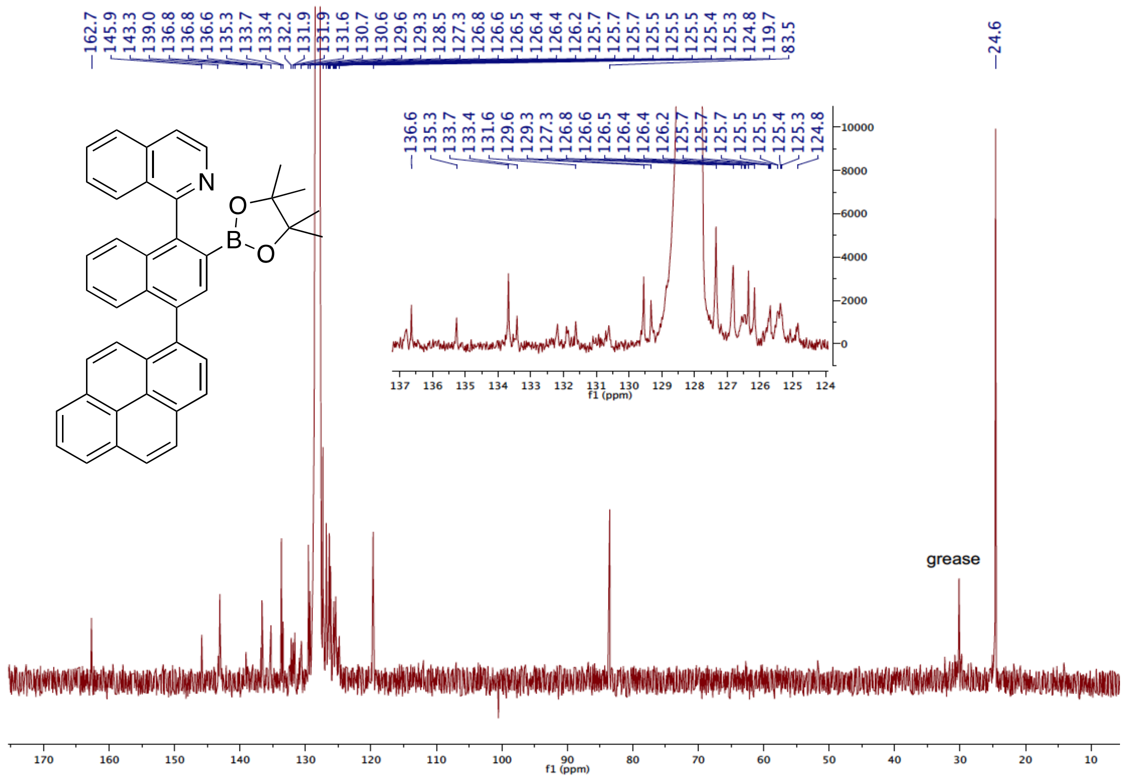

Figure S37. ^{13}C NMR spectrum (C_6D_6, 125 MHz, 80 °C) of **18**.

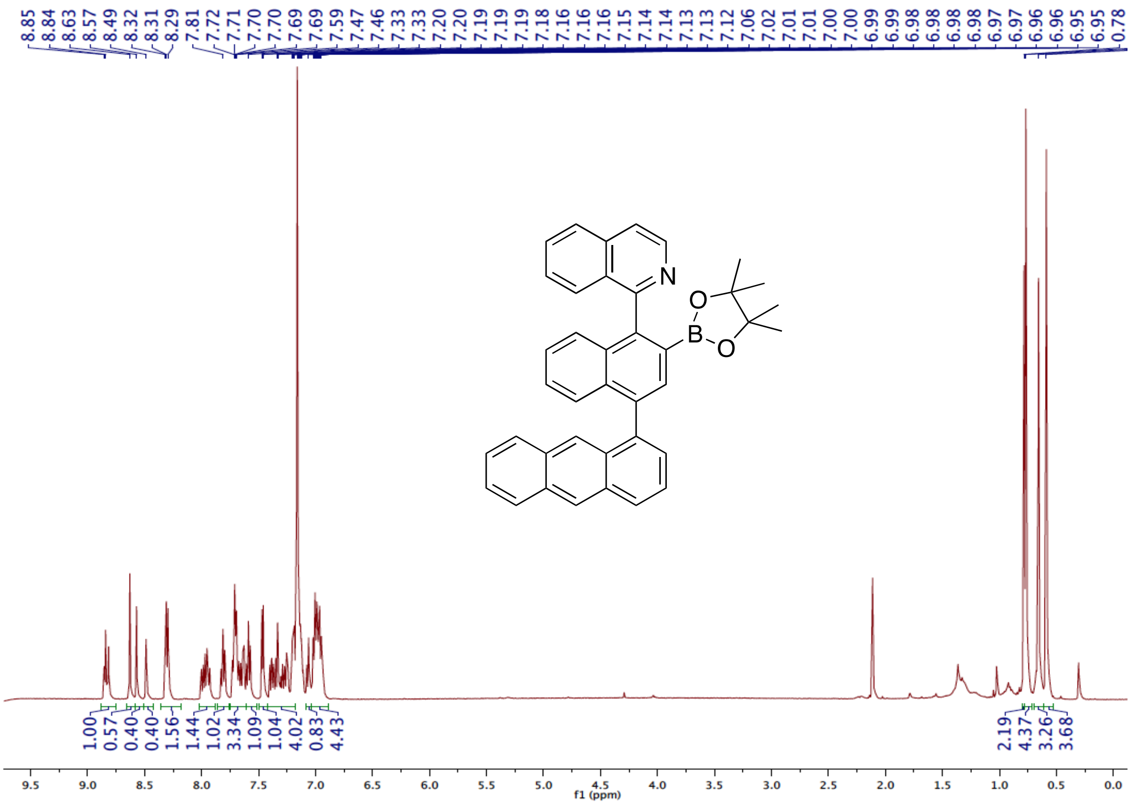

Figure S38. ^{1}H NMR spectrum (C_6D_6, 500 MHz, 25 °C) of **19**.

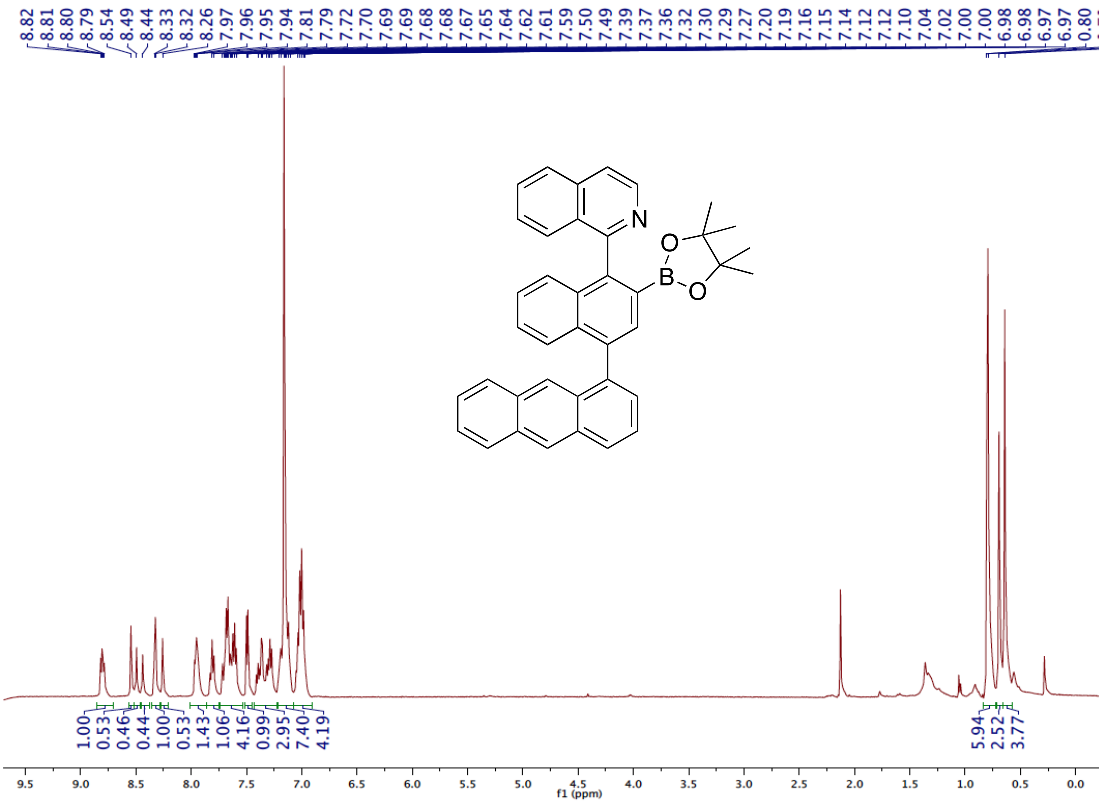

Figure S39. ^{1}H NMR spectrum (C_6D_6, 500 MHz, 80 °C) of **19**.

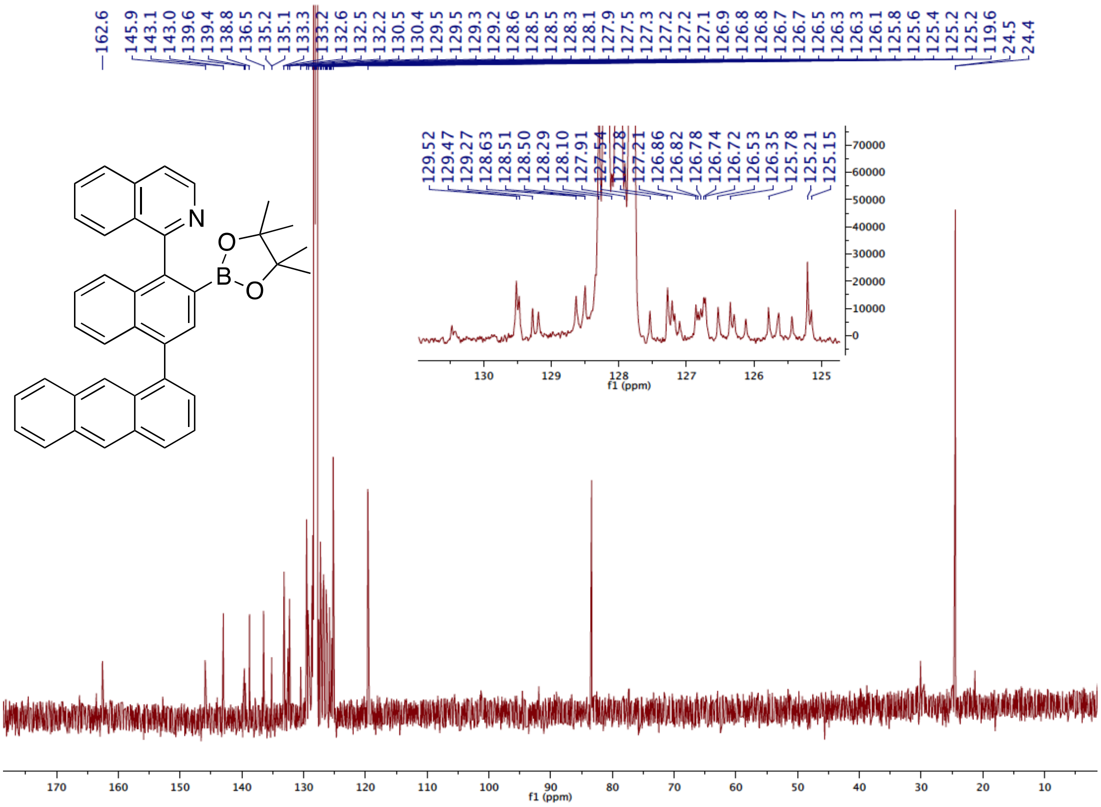

Figure S40. ^{13}C NMR spectrum (C_6D_6, 125 MHz, 80 °C) of **19**.

3. HPLC traces for the dyes 16–19

The chromatographic analysis was performed on a chiral column (IA, *n*-hexane/isopropanol 98:2, 1 mL/min, 30 ºC).

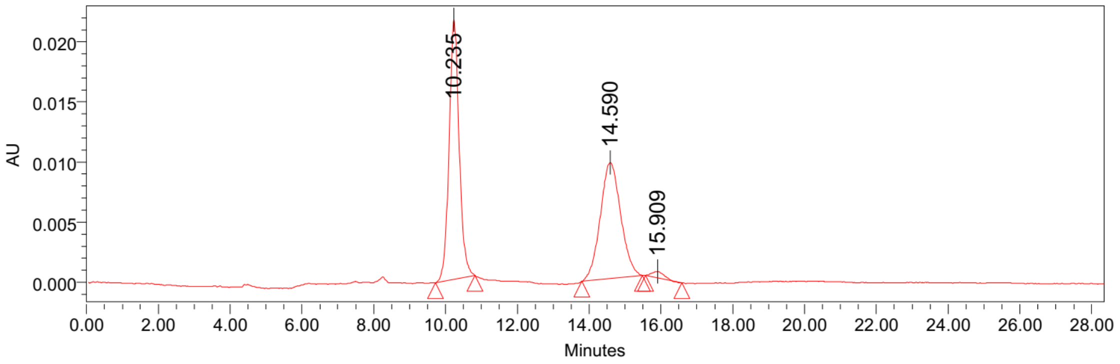

Figure S41. HPLC trace for dye **16**.

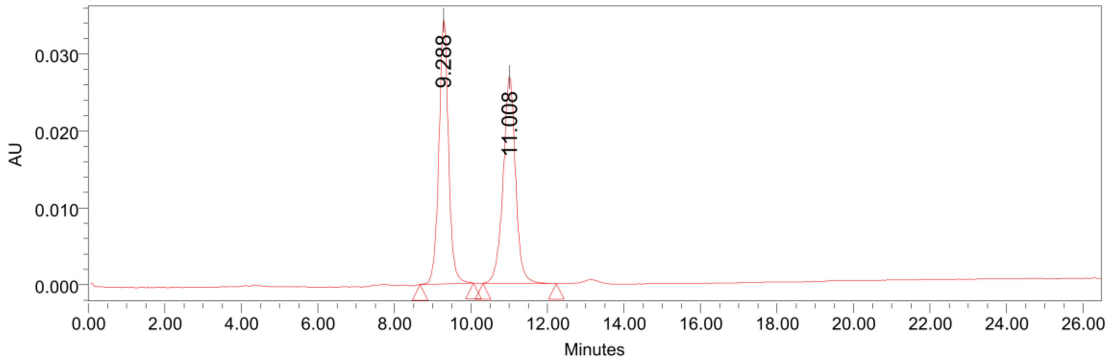

Figure S42. HPLC trace for dye **17**.

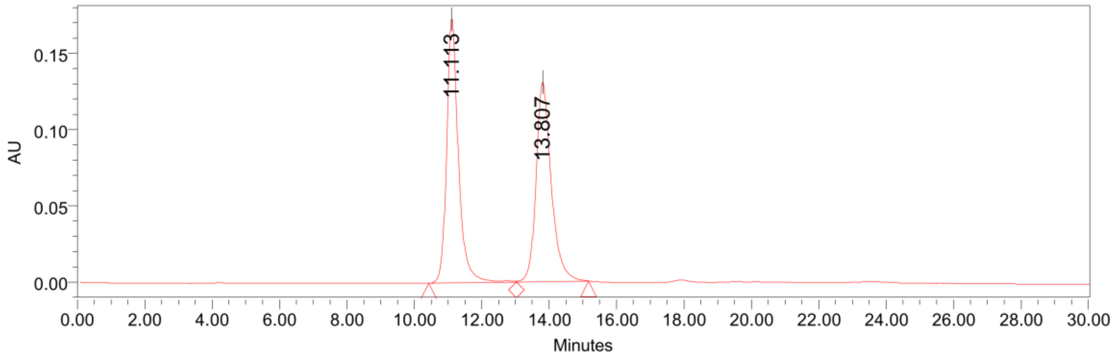

Figure S43. HPLC trace for dye **18**.

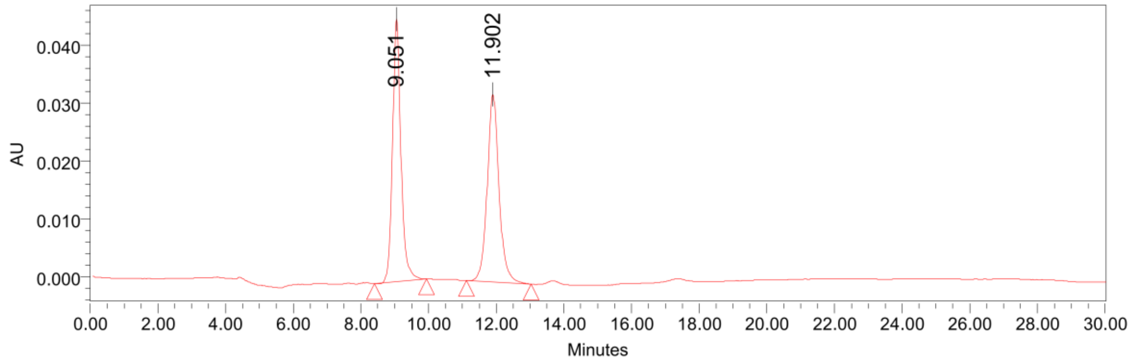

Figure S44. HPLC trace for dye **19**.

4. Formation of fluoroboronate complexes – ESIMS and ^{11}B NMR

0.5 mL of a solution of the boronic ester in acetonitrile (ca. 0.01 M) was treated with 10 μL of a solution of TBAF (ca. 0.5 M in CH_3CN). After 5 min of stirring the crude was analyzed by ESIMS.

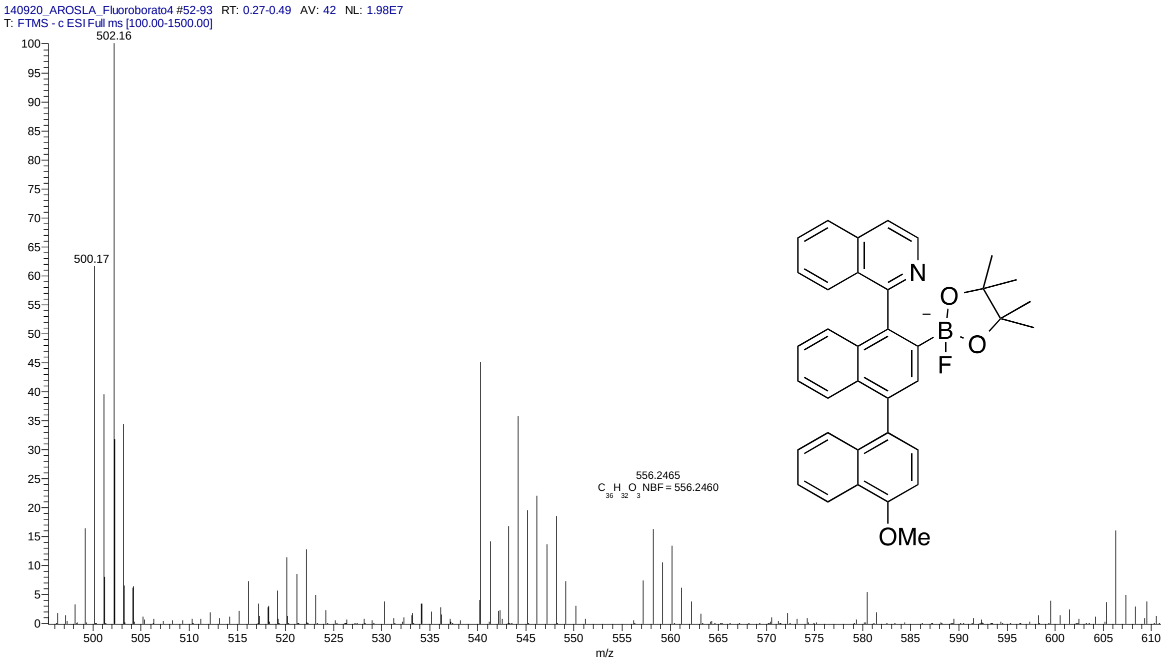

Figure S45. ESIMS of the fluoroboronate complex of **16**.

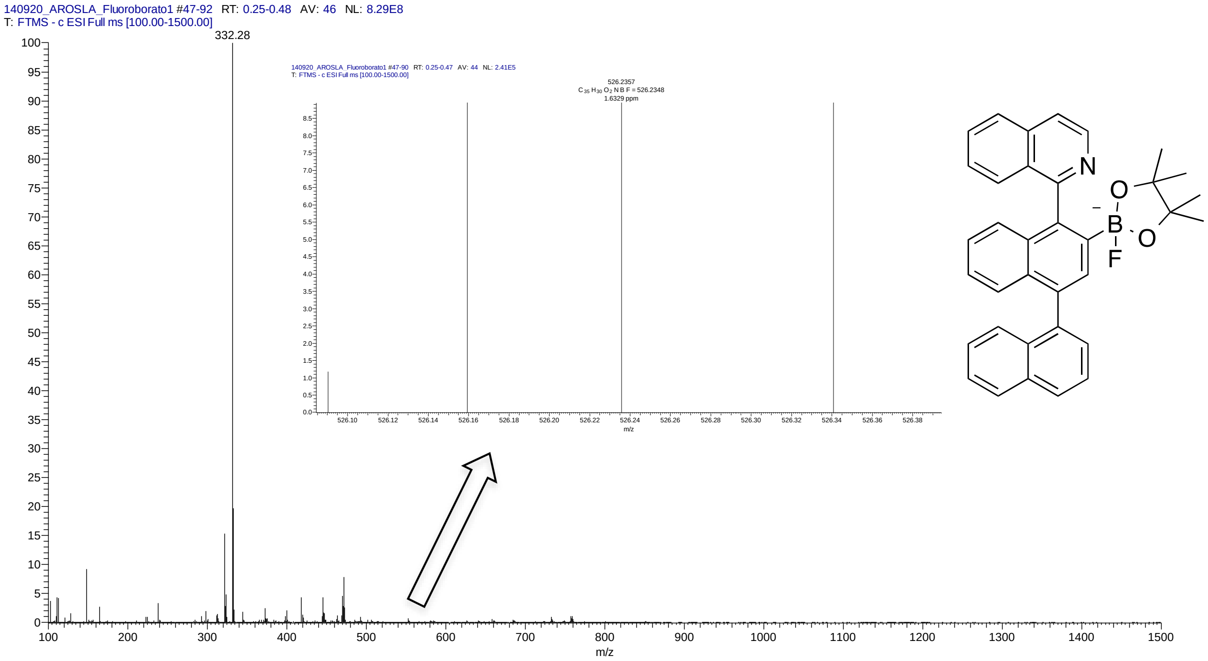

Figure S46. ESIMS of the fluoroboronate complex of **17**.

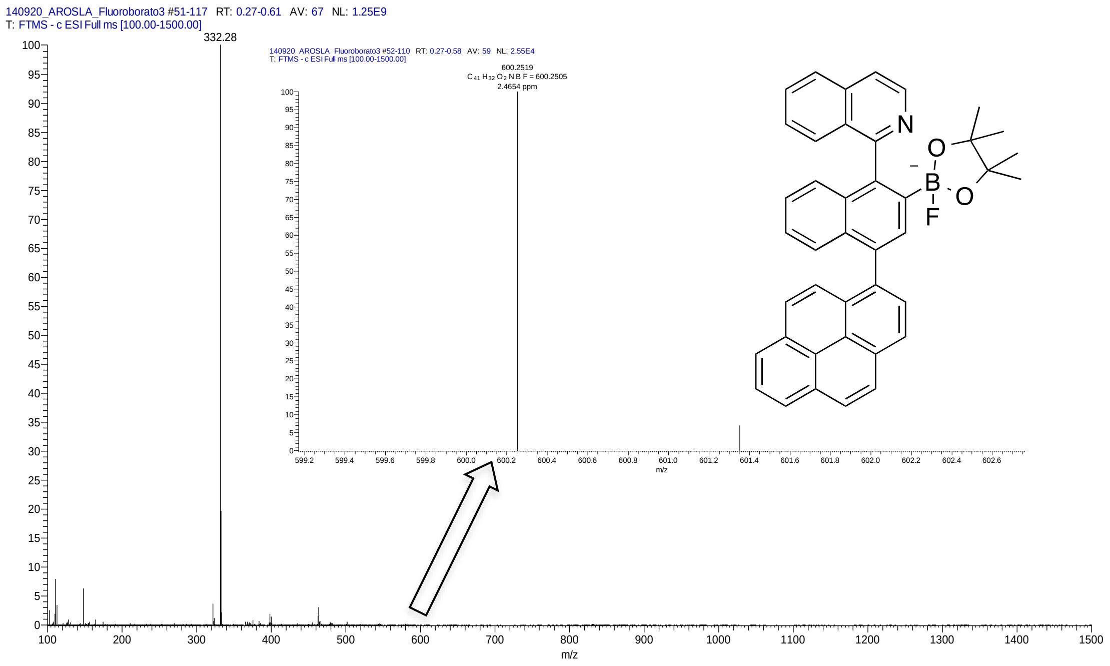

Figure S47. ESIMS of the fluoroboronate complex of **18**.

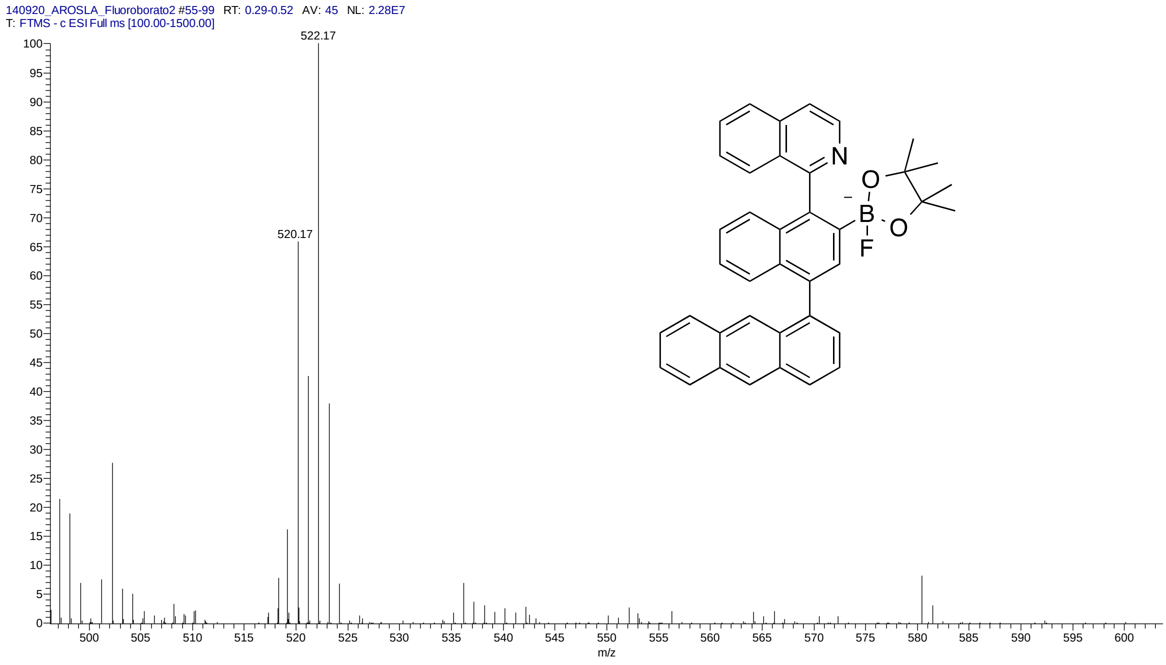

Figure S48. ESIMS of the fluoroboronate complex of **19**.

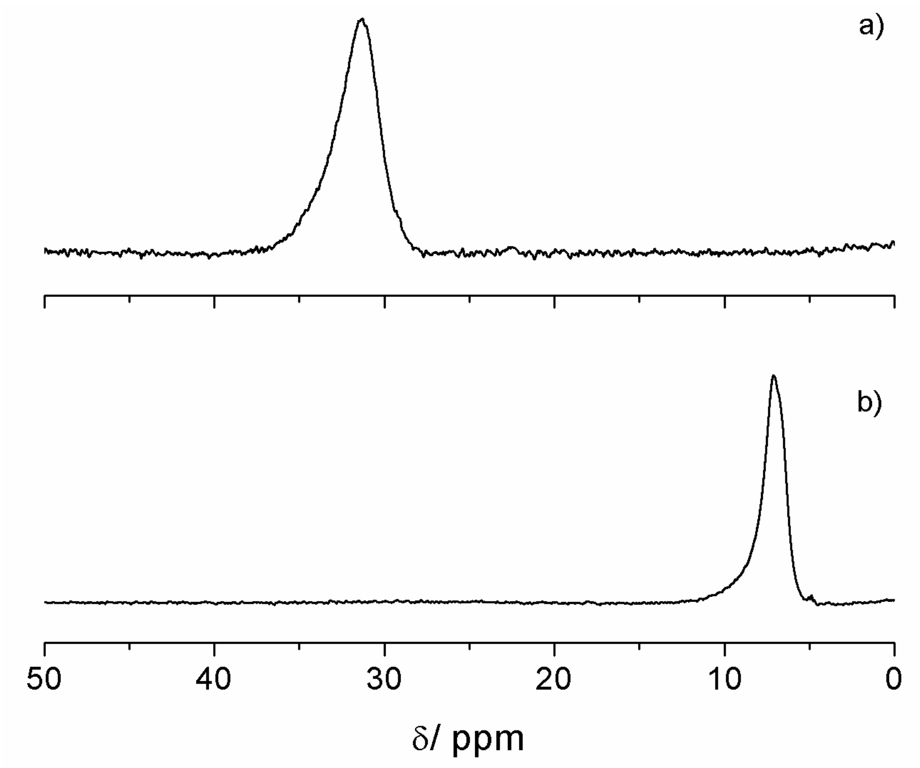

Figure S49. ^{11}B NMR spectra of dye **17** before (a) and after addition (b) of 1 equiv TBAF.
